# Synthesis of the A−F Fragment of the Pacific Ciguatoxin CTX3C by Iterative Ring‐Closing Metathesis and Tsuji–Trost Allylation

**DOI:** 10.1002/chem.202303121

**Published:** 2023-11-02

**Authors:** Myron Triantafyllakis, Sam Alexander, Sophie Woolford, Claire Wilson, J. Stephen Clark

**Affiliations:** ^1^ School of Chemistry Joseph Black Building University of Glasgow University Avenue Glasgow G12 8QQ UK

**Keywords:** ciguatoxin, iterative ring construction, polyether, ring-closing metathesis, Tsuji–Trost allylation

## Abstract

The fully functionalized A−F fragment of the Pacific ciguatoxin CTX3C has been synthesized from a derivative of D‐glucal, which serves as the B‐ring. Rings A and C were introduced to either side of ring B by ring‐closing diene and enyne metathesis (RCM). The seven‐membered D‐ring and eight‐membered E‐ring were assembled by iterative use of a six‐step reaction sequence in which RCM was used for ring construction and Tsuji–Trost allylation was employed for subsequent stereoselective functionalization. The nine‐membered F‐ring was formed by use of an RCM reaction and bears the functionality required for attachment of the I−M fragment and subsequent closure of rings G and H.

## Graphical abstract

The fully functionalized A−F ring system of the Pacific ciguatoxin CTX3C suitable for coupling to the I−M fragment of the natural product, has been synthesized from a simple glucal derivative. A six‐step sequence that features ring‐closing metathesis and stereoselective palladium‐mediated allylation has been deployed in an iterative manner to construct the medium‐sized D and E rings.

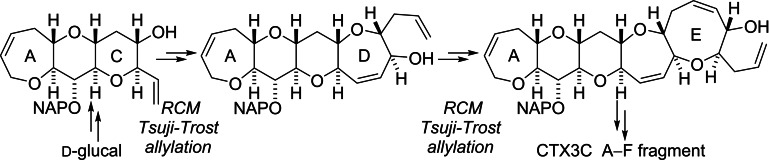

## Introduction

**Figure 1:**
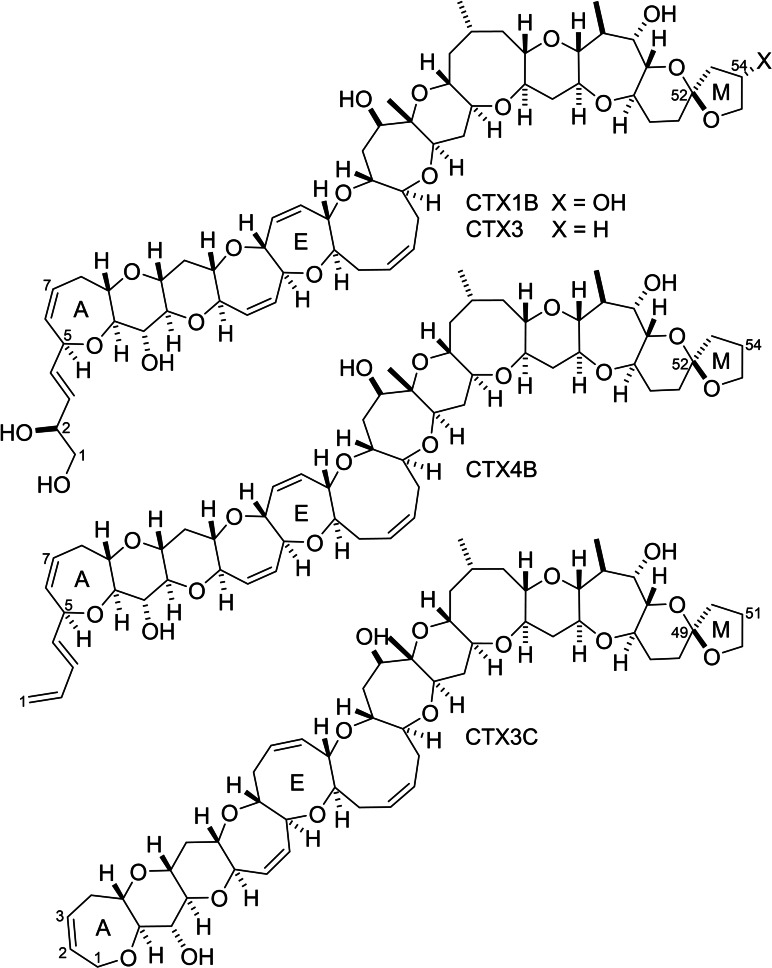
Representative Pacific ciguatoxins.

The ciguatoxins are fused polyether natural products of marine origin and are members of a burgeoning sub‐class within the polyether natural product family.^[1]^ These compounds are potent neurotoxins and are responsible for ciguatera poisoning in humans, a serious condition that is characterized by harmful neurological, gastrointestinal, and cardiovascular effects.^[2]^ To date, more than 30 ciguatoxins have been isolated from various marine dinoflagellates or from higher organisms in which the toxins accumulate through the food chain.^[3]^ Ciguatoxins have been isolated from organisms collected in the Pacific Ocean, the Indian Ocean and the Caribbean Sea; there are subtle but distinct differences in structure between ciguatoxins isolated from each region.^[4]^ Most of the ciguatoxins that have been fully characterized have been isolated from samples collected in the Pacific Ocean (Figure 1). The Pacific ciguatoxin family comprises two distinct structural sub‐classes. In the first group, the E‐ring is seven‐membered, and the A‐ring possesses a four‐carbon side chain (at position 5) that can be either 1,3‐butadienyl (as in CTX4B) or dihydroxybutenyl (as in CTX1B and CTX3).[^1b^, ^1d^] In the second group, the A‐ring does not bear an alkyl substituent and the E‐ring is eight‐membered (as in CTX3C).[^1d^, ^5^] Both Pacific ciguatoxin sub‐classes have members that possess additional hydroxyl groups and/or an oxo group; oxygen substituents are usually located in rings A, L or M, or in the four‐carbon A‐ring side chain when present. The structures of the B−D and F−K polyether arrays are conserved throughout the entire Pacific ciguatoxin family.

The size and structural complexity of the ciguatoxins combined with the large number of stereogenic centers they possess makes them extremely challenging targets for total synthesis. The prevalence of cyclic ethers with ring sizes 7–9 bestows additional complexity and renders the synthetic task even more daunting. The fascinating structures of the ciguatoxins combined with the synthetic challenges they present makes them attractive targets for total synthesis and numerous research groups have investigated the construction of these natural products over the past three decades.^[6]^ Although many innovative new synthetic methods have been developed and significant portions of the ciguatoxins have been constructed in the course of this work, there has been a dearth of completed total syntheses. To date, the only total syntheses to have been accomplished are the syntheses of CTX3C, 51‐hydroxy‐CTX3C and CTX1B reported by Hirama and co‐workers in 2001 and 2006,[^7^, ^8^] and the synthesis of CTX1B reported by Isobe and Hamajima in 2009 (Figure 1).^[9]^ Although they are lengthy, these very impressive syntheses have set an extremely high benchmark for the field of marine polyether synthesis.

Over the past 25 years we have invented several strategies for the efficient synthesis of fused polycyclic ether arrays of the type found in marine polyether natural products. In the course of this work, we have used iterative and bidirectional strategies to assemble major fragments found in members of the brevetoxin, gambieric acid and ciguatoxin classes.[^10^, ^11^, ^12^] In our previous studies concerning the synthesis of CTX3C, we have prepared both the A−E, and I−L fragments from simple monocyclic precursors but without the functionality required for further elaboration.[^11^, ^12^] We now describe a synthesis of the entire A−F array, suitable for fragment coupling, by iterative application of a reaction sequence that comprises stereoselective Tsuji–Trost allylation and ring‐closing metathesis (RCM) reactions.^[13]^

**Figure 2:**
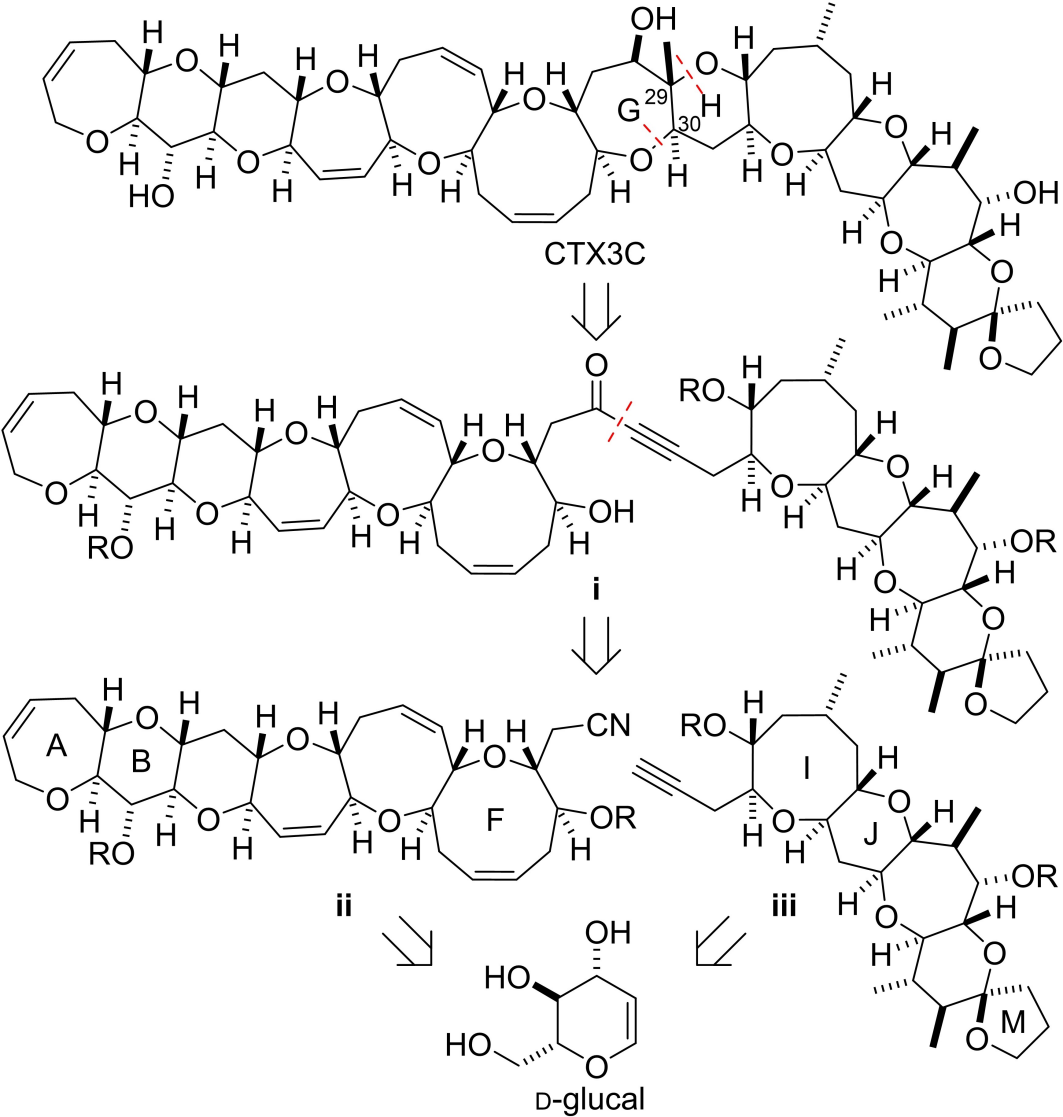
Disconnection of CTX3 C into the A−F fragment **ii** and the I−M fragment **iii**.

Our overall strategy and main disconnection of CTX3C is shown in Figure 2. Conversion of the G‐ring hydroxyl group into a carbonyl group and disconnection of the ether bonds in rings G and H (at positions 29 and 30) affords propargylic ketone **i**. This disconnection implies construction of ring G in the forward synthesis by nucleophilic addition of F‐ring hydroxyl group to the propargylic ketone. Further disconnection between the alkyne and carbonyl group in **i** reveals the A−F array **ii** and the I−M array **iii** as coupling partners. The syntheses of both fragments will commence from D‐glucal, which is readily available from the chiral pool.

## Results and Discussion

**Scheme 1:**
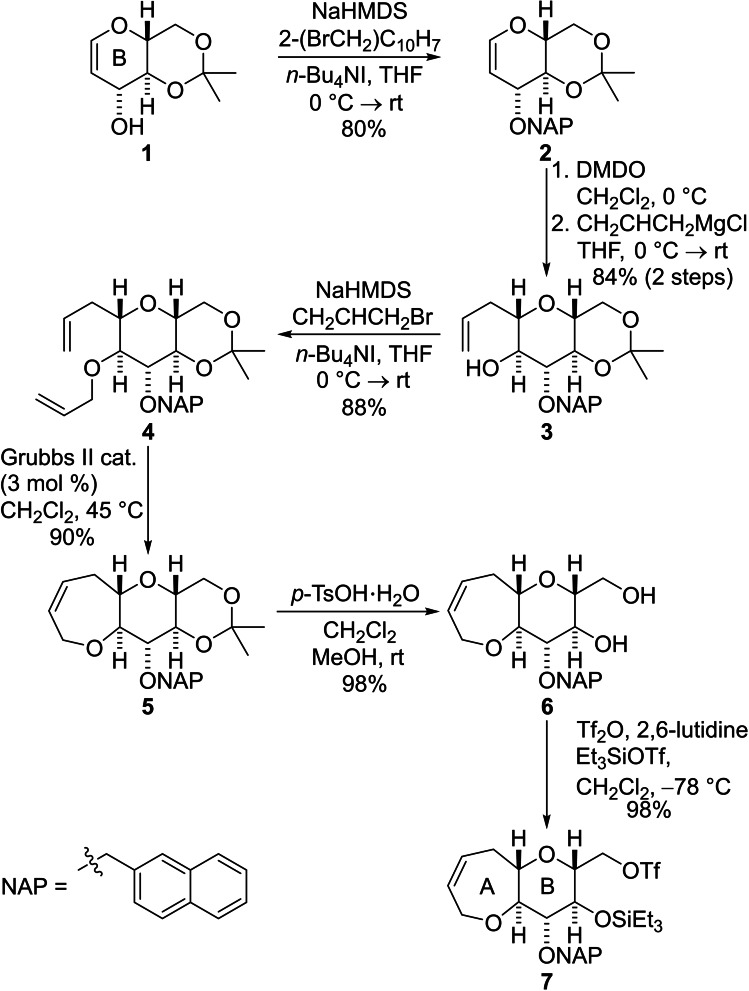
Synthesis of the AB fragment from protected D‐glucal (**1**).

The starting material for our synthesis of the A−F fragment is the commercially available alcohol **1**, which was prepared on multigram scale from D‐glucal.^[14]^ Protection of the hydroxyl group as a 2‐naphthylmethyl (NAP) ether afforded the enol ether **2** in good yield (Scheme 1). The NAP protecting group was selected for hydroxyl group protection because Hirama and co‐workers had shown that it could be removed efficiently in the final step of their synthesis of CTX3C.^[7b]^ The enol ether **2** was then subjected to highly diastereoselective epoxidation by treatment with dimethyldioxirane (DMDO) and the resulting epoxyacetal was ring‐opened at the anomeric position by treatment with allylmagnesium chloride to produce the alcohol **3** in excellent yield.^[15]^ Conversion of the alcohol **3** into the allyl ether **4** was followed by a ring‐closing metathesis (RCM) reaction, which delivered the bicyclic ether **5** in excellent yield.^[11]^ Cleavage of the acetonide under standard conditions produced the diol **6** and subsequent formation of the primary triflate and triethylsilyl (TES) protection of the secondary hydroxyl group was performed in one pot to give the triflate **7** in excellent yield.

**Scheme 2:**
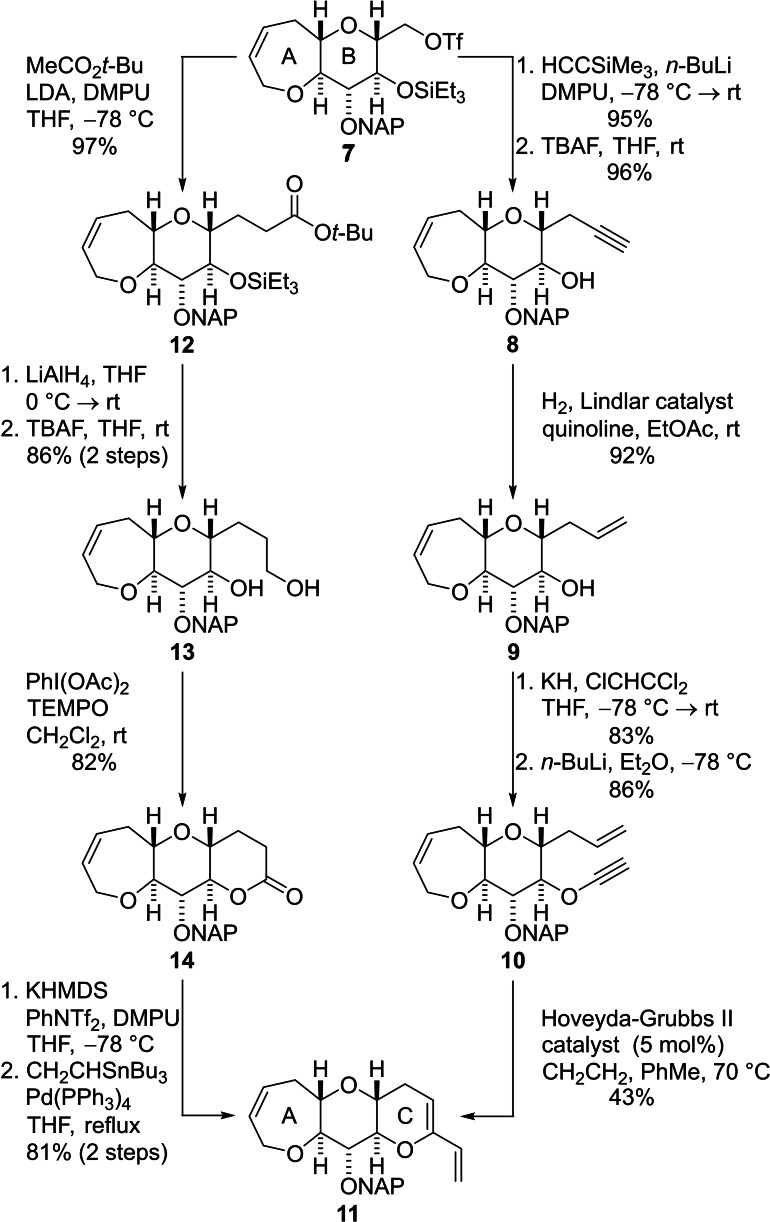
Two routes for elaboration of the triflate **7** to give the ABC fragment **11**.

Conversion of the bicyclic fragment **7** into the A−C fragment was accomplished by use of the two routes presented in Scheme 2. In the first route, reaction of the triflate **7** with lithium (trimethylsilyl)acetylide in the presence of *N*,*N’*‐dimethylpropyleneurea (DMPU) and subsequent cleavage of the TES ether afforded the alcohol **8**. Partial hydrogenation of the alkyne, promoted by Lindlar catalyst, delivered the alcohol **9** and the well‐established procedure developed by Greene and co‐workers was used to convert this compound into the alkynyl ether **10** with high yield.^[16]^ An enyne RCM reaction mediated by the Grubbs‐Hoveyda II catalyst was then used to transform the bicyclic enyne into the required tricyclic enol ether **11**.^[17]^ In the second route (Scheme 2), the triflate **7** was reacted with the lithium enolate generated by deprotonation of *t*‐butyl acetate with LDA. The resulting ester **12** was reduced with lithium aluminium hydride and the TES ether was cleaved by treatment with tetra‐*n*‐butylammonium fluoride (TBAF) to produce the diol **13**. Lactonization to form ring C was then accomplished by oxidation of the diol **13** with a mixture of TEMPO and iodobenzene diacetate.^[18]^ The lactone **14** was converted into an enol triflate and a palladium mediated Stille coupling with tributyl(vinyl)tin afforded the A−C fragment **11**.^[19]^ Both synthetic sequences from the bicyclic triflate **7** to the tricyclic fragment **11** comprise six steps, but reactions in the route involving Stille coupling were easier to perform on a large scale and the overall yield (55 %) for this route was higher than that involving enyne RCM (26 %).

**Scheme 3:**
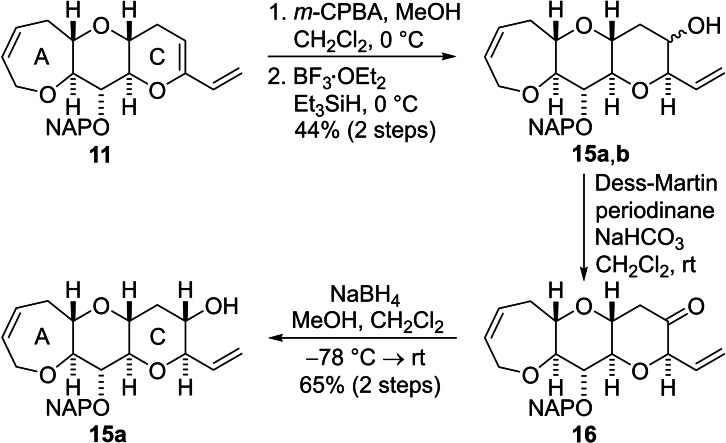
Functionalization of ring C to permit extension of the fused polycyclic ether array.

Chemoselective functionalization of the A−C fragment **11** was required to allow construction of ring D (Scheme 3). Epoxidation of the C‐ring enol ether by reaction with *m*‐CPBA was followed by reductive ring opening of the epoxy acetal by treatment with a mixture of triethylsilane and boron trifluoride etherate.^[20]^ A diastereomeric mixture of the alcohols **15 a**,**b** was obtained from this sequence of reactions, but the stereogenic center at the allylic position of ring C was created with the required configuration. The diastereomeric alcohols were not separated at this stage; instead, the mixture was oxidized and the resulting ketone **16** was reduced immediately to give the required alcohol **15 a** in a highly diastereoselective manner.

**Scheme 4:**
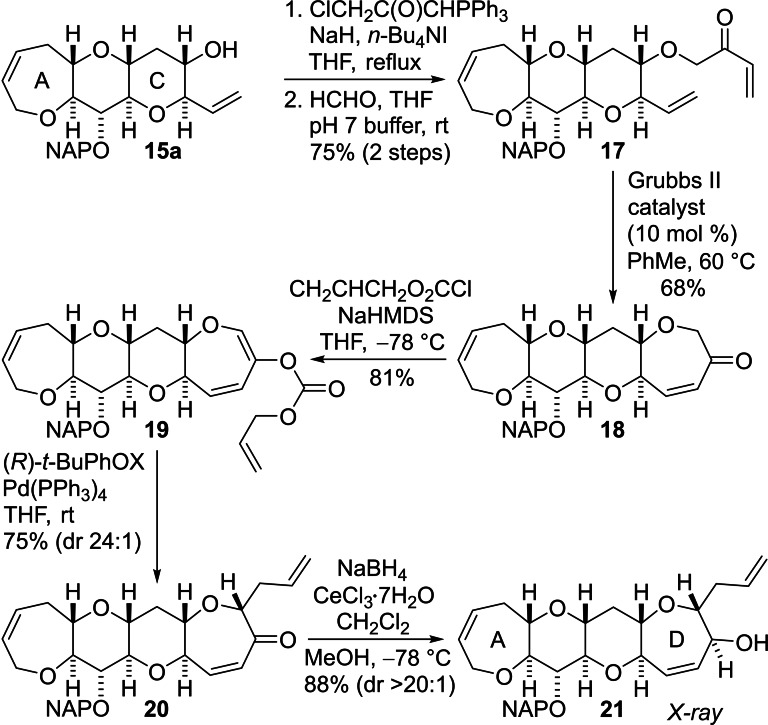
Installation of ring D by sequential RCM and stereoselective palladium‐mediated Tsuji–Trost allylation.

Synthesis of the fully functionalized A−C fragment **15 a** allowed the pivotal sequence of RCM and Tsuji–Trost allylation to be implemented for the first time (Scheme 4).^[13]^ The alcohol was first alkylated with 1‐chloro‐3‐(triphenylphosphoranylidene)‐2‐propanone and the resulting phosphonium ylide was then treated with aqueous formaldehyde in the presence of pH 7 buffer to give the enone **17** in good yield.^[21]^ Ring D was then constructed by treatment of the triene **17** with the Grubbs second generation ruthenium catalyst. The resulting tetracyclic ketone **18** was deprotonated and the enolate was *O*‐acylated by reaction with allyl chloroformate to give the enol carbonate **19**.^[13]^ Attachment of the allyl chain to ring D was then accomplished by stereoselective Tsuji–Trost allylation mediated by a palladium complex of the (*R*)‐*t*‐butyl‐PHOX ligand, a procedure that we had already demonstrated is highly effective for functionalization of simpler medium‐sized cyclic ethers.[^12^, ^13^, ^22^] The allylated enone **20** was obtained in excellent yield and with a high level of diastereocontrol. Reduction of the carbonyl group under Luche conditions^[23]^ then delivered the crystalline allylic alcohol **21** with a high degree of diastereocontrol. The structure of the alcohol **21** was established by use of X‐ray crystallography.^[24]^

**Scheme 5:**
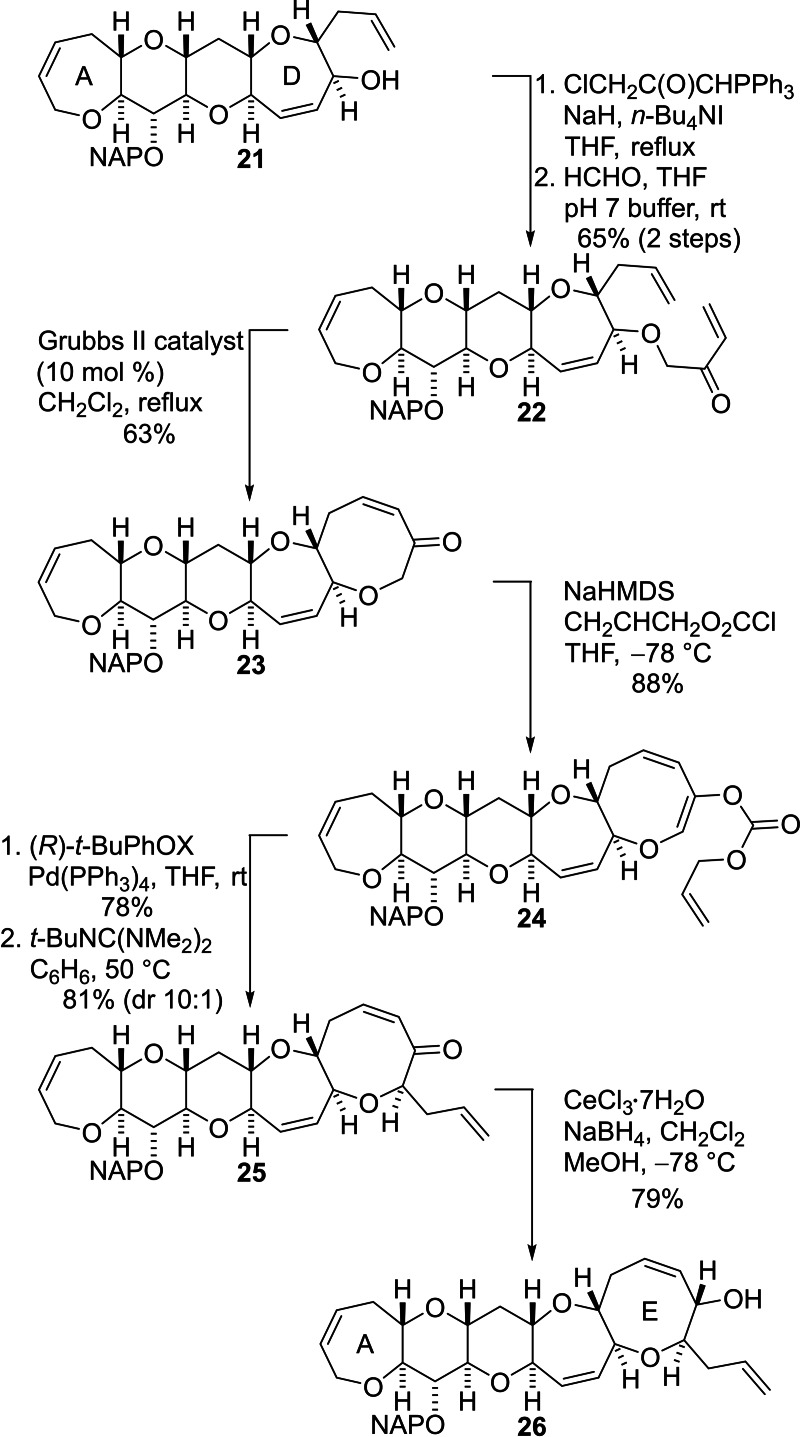
Installation of ring E by sequential RCM and stereoselective palladium‐mediated Tsuji–Trost allylation.

Ring E was then constructed by repetition of the reaction sequence employed to assemble ring D (Scheme 5). The alcohol **21** was subjected to *O*‐alkylation with 1‐chloro‐3‐(triphenylphosphoranylidene)‐2‐propanone and a subsequent Wittig reaction of the intermediate phosphonium ylide with formaldehyde produced the enone **22** in good yield. The Grubbs second generation ruthenium catalyst was used to perform RCM to give the eight‐membered E‐ring. The resulting pentacyclic ketone **23** was deprotonated and the enolate was treated with allyl chloroformate to produce the enol carbonate **24**. Stereoselective Tsuji–Trost allylation was then accomplished by reaction with a palladium complex of the (*R*)‐*t*‐butyl‐PHOX ligand. In this case, although the required ketone **25** was the major product, the diastereomeric ratio was variable and so the mixture of diastereomers was treated with Barton's base to maximize the amount of the required diastereomer **25** produced when necessary.^[25]^ Luche reduction of the carbonyl group of the enone **25** delivered the A−E fragment **26** in good yield and in a highly diastereoselective manner. The ^1^H and ^13^C NMR data and other data for the alcohol **26** matched those reported by Hirama and co‐workers for this compound during their synthesis of CTX3C.^[26]^

**Scheme 6:**
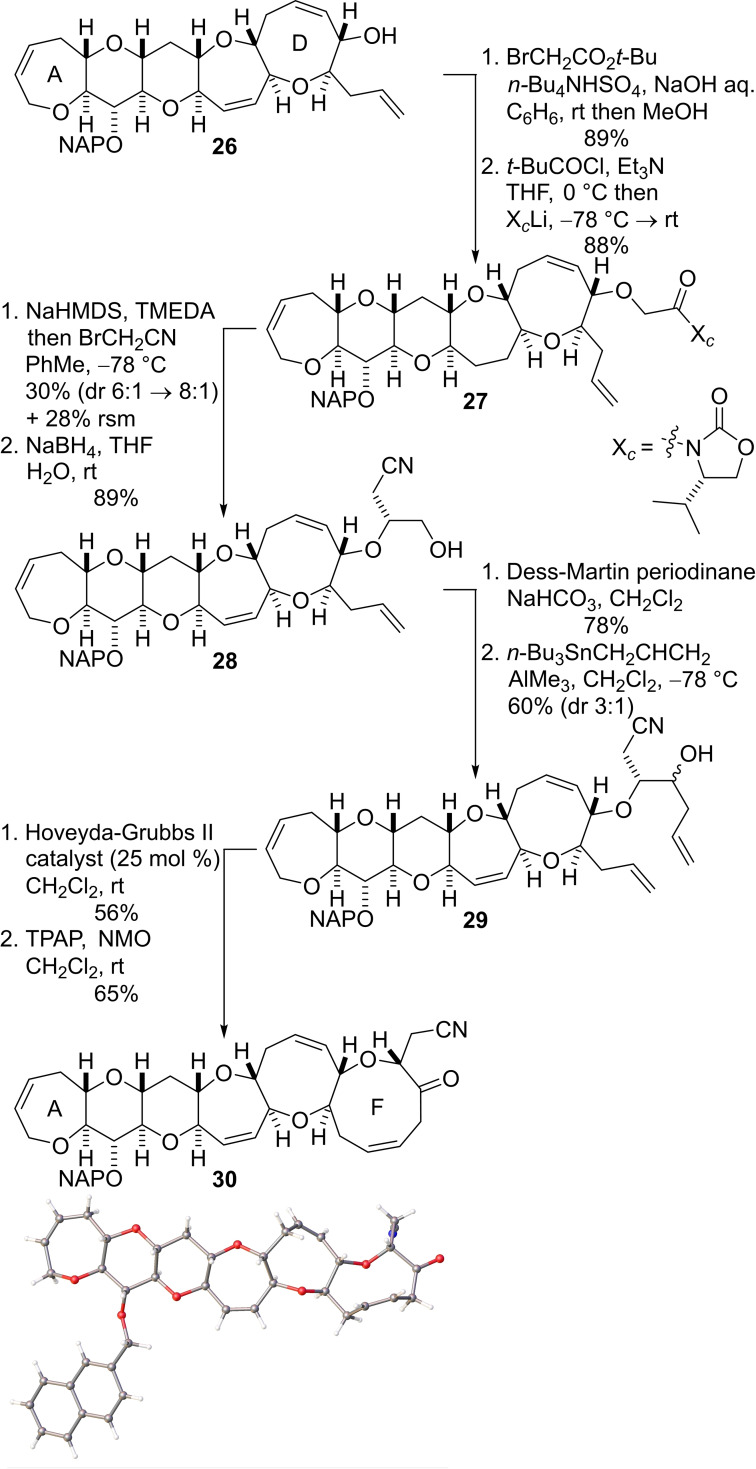
Construction of ring F to complete the A−F fragment of CTX3C.

Conversion of the allylic alcohol **26** into the hexacyclic A−F array was accomplished by the sequence of reactions shown in Scheme 6, with RCM again serving as the key reaction to form the nine‐membered F‐ring.[^10b^, ^27^] Our route to the precursor (**29**) required for the RCM reaction was analogous to that used by Crimmins and co‐workers during their synthesis of the E‐ring of brevetoxin A.^[28]^ The free hydroxyl group of the pentacyclic alcohol **26** was first alkylated with *t*‐butyl bromoacetate under phase transfer conditions. In situ ester hydrolysis followed by acidic workup produced the free carboxylic acid. Conversion of the acid into a mixed anhydride by reaction with pivaloyl chloride was followed by addition of lithiated (*S*)‐4‐isopropyl‐2‐oxazolidinone gave the imide **27**.^[29]^ Subsequent deprotonation with sodium bis(trimethylsilyl)amide and alkylation of the resulting enolate with bromoacetonitrile proceeded in modest yield and with good diastereoselectivity. Removal of the Evans chiral auxiliary with sodium borohydride in wet THF then delivered the alcohol **28** in excellent yield.^[30]^ The alcohol was oxidized according to the Dess–Martin protocol and the resulting aldehyde was reacted allyltri‐*n*‐butyltin in the presence of trimethylaluminium at low temperature to afford a diastereomeric mixture (3 : 1 ratio) of the alcohols **29** in 60 % yield. The mixture of alcohols was then subjected to RCM mediated by the Hoveyda‐Grubbs II catalyst to give the required hexacyclic alcohol in 56 % yield as an inseparable mixture of diastereomers. Oxidation of the homoallylic alcohol with tetra‐*n*‐propylammonium perruthenate (TPAP)[^31^, ^32^] afforded the crystalline ketone **30** (Scheme 6). The structure of hexacyclic ketone **30** was determined unambiguously by single crystal X‐ray diffraction studies and NMR analysis (Scheme 6).^[24]^

## Conclusions

In summary, the fused hexacyclic ketone **30**, which corresponds to the fully functionalized A−F ring system of CTX3C and is suitable for coupling to the I−M fragment of the natural product, has been synthesized from the glucal derivative **1** in a total of 37 steps. The key feature of the route to this intermediate is iterative application of a six‐step reaction sequence to construct rings D and E that features RCM followed by stereoselective Tsuji–Trost allylation of the newly created medium‐sized cyclic ether.

## Supporting Information

The authors have cited additional references within the Supporting Information.^[34]^

## Conflict of interest

The authors declare no conflict of interest.

1

## Supporting information

As a service to our authors and readers, this journal provides supporting information supplied by the authors. Such materials are peer reviewed and may be re‐organized for online delivery, but are not copy‐edited or typeset. Technical support issues arising from supporting information (other than missing files) should be addressed to the authors.

Supporting Information

## Data Availability

The data that support the findings of this study are available in the supplementary material of this article.
