# Supplementary material for: Synthesis of the A−F Fragment of the Pacific Ciguatoxin CTX3C by Iterative Ring‐Closing Metathesis and Tsuji–Trost Allylation
Source: Chemistry. 2023 Nov 2;29(72):e202303121. doi: 10.1002/chem.202303121 (PMC10946863; doi:10.1002/chem.202303121)
Supplement: Supplementary file 1 — Supporting Information [file CHEM-29-0-s001.pdf]

Chemistry–A European Journal

Supporting Information

Synthesis of the A–F Fragment of the Pacific Ciguatoxin CTX3C by Iterative Ring-Closing Metathesis and Tsuji–Trost Allylation

Myron Triantafyllakis, Sam Alexander, Sophie Woolford, Claire Wilson, and J. Stephen Clark*

Table of Contents

**General**	**S2**
**Experimental Procedures**	**S3**
**X-Ray Data**	**S29**
**References**	**S32**
**NMR Data**	**S33**

General

Unless stated otherwise, reactions were performed in flame-dried glassware using anhydrous solvents under argon. Organic solvents were dried using a Pure Solv solvent purification system.

Column chromatography was performed silica gel (Fluorochem LC60A, 35–70 micron or Merck Geduran Si60, 40–63 micron under forced flow. Petroleum ether used for column chromatography was the 40–60 °C fraction.

Reactions were monitored by thin-layer chromatography (TLC) on Fisher and Merck silica gel 60 covered alumina plates. TLC plates were visualised under UV light and developed with a solution of potassium permanganate or anisaldehyde.

Specific rotations were recorded using an Autopol V automatic polarimeter at 589 nm.

IR spectra were recorded using a type IIa diamond single reflection element on a Shimadzu FTIR-8400S instrument. The IR spectrum of the compound was recorded directly on a thin film (liquid) or powder (solid) at ambient temperature.

^{1}H NMR spectra were recorded on a Bruker AVIII 400 MHz, Bruker AVIII 500 MHz or Bruker AVIII HD 600 MHz spectrometer at ambient temperature. ^{13}C NMR spectra were recorded at 101 MHz, 126 MHz or 151 MHz at ambient temperature.

HRMS data were obtained by the analytical services of the University of Glasgow on a Jeol MStation JMS-700 (EI), a Bruker micro TOFq (ESI) or an Agilent 6546 LC/Q-TOF (ESI, APCI) mass spectrometer.

Melting points were recorded with an Electrothermal IA 9100 apparatus.

Experimental Procedures

Enol ether 2

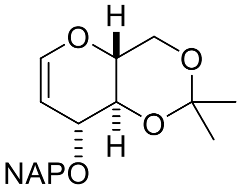

2

To a solution of the alcohol **1**[1] (36.9 g, 0.198 mol) in anhydrous DMF (1.0 L) was added sodium bis(trimethylsilyl)amide (109 mL of a 2.0 M solution in THF, 0.218 mol) dropwise at 0 °C and the resulting solution was stirred at 0 °C for 15 min. 2-(Bromomethyl)naphthalene (48.24 g, 218.2 mmol) was added followed by tetra-*n*-butylammonium iodide (731 mg, 1.98 mmol). The solution was warmed to room temperature and stirred for further 4 h before the reaction was quenched by addition of a saturated aqueous solution of sodium bicarbonate (500 mL). The mixture was extracted with diethyl ether (4 × 500 mL) and the combined organic extracts were washed with water (3 × 300 mL) and brine (300 mL), then dried (magnesium sulfate) and filtered. The solvent was removed by evaporation under reduced pressure and the resulting orange solid was recrystallized from petroleum ether to afford the ether **2** as a pale-yellow solid (51.8 g, 80%). R_f 0.22 (petroleum ether:diethyl ether, 9:1). Mp 62–63 °C. $[\alpha]_D^{21}$ −12.1 (*c* = 1.40 in $CHCl_3$). ^{1}H NMR (500 MHz, $CDCl_3$) δ 7.88–7.77 (4H, m), 7.48 (3H, m), 6.33 (1H, dd, *J* = 6.2, 1.7 Hz), 4.92 (1H, d, *J* = 12.4 Hz), 4.84 (1H, d, *J* = 12.4 Hz), 4.80 (1H, dd, *J* = 6.2, 2.0 Hz), 4.26 (1H, ddd, *J* = 7.3, 2.0, 1.7 Hz), 4.08 (1H, dd, *J* = 10.3, 7.3 Hz), 3.95 1H, (1H, dd, *J* = 10.8, 5.7 Hz), 3.86 (1H, dd, *J* = 10.8. 10.3 Hz), 3.74 (1H, ddd, *J* = 10.3, 10.3, 5.7 Hz), 1.52 (3H, s), 1.46 (3H, s). ^{13}C NMR (126 MHz, $CDCl_3$) δ 144.5, 136.2, 133.3, 132.9, 128.0, 127.84, 127.7, 126.3, 126.0, 125.7, 102.5, 99.6, 73.7, 72.6, 71.7, 69.6, 61.8, 29.1, 19.1. $\nu_{max.}$ (film) 2994, 2891, 1713, 1640, 941, 868, 814, 755, 754 cm^{-1}. HRMS (ESI) calcd. for $C_{20}H_{22}NaO_4$ $[M+Na]^+$ 349.1410, found 349.1397. Anal. calcd. for $C_{20}H_{22}O_4$: C, 73.60; H, 6.79, found C, 73.76; H, 6.83.

Alcohol 3

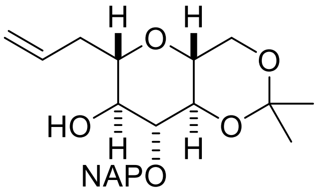

3

To a solution of enol ether **2** (35.0 g, 0.107 mol) in dichloromethane (400 mL), at 0 °C, was added over 15 min a freshly distilled solution of dimethyldioxirane (1.68 L of a 0.07 M solution in acetone, 0.118 mol) from an addition funnel. The solution was stirred at 0 °C until consumption of the starting material was indicated by NMR analysis of the crude mixture. The solvent was evaporated under

reduced pressure. The residue was dissolved in dichloromethane (300 mL), dried (magnesium sulfate), filtered and the solvent was evaporated under reduced pressure. The crude epoxide was dried for 1 h under high vacuum and was used for the next reaction without purification.

The crude epoxide was dissolved in anhydrous THF (500 mL) and the solution was cooled to 0 °C. Then solution of allylmagnesium chloride (660 mL of a 1.0 M solution in THF, 0.660 mol) was added slowly through a cannula. After the addition was complete the solution was warmed to room temperature and stirred for 2 h. The reaction was cooled to 0 °C and a saturated aqueous solution of ammonium chloride (500 mL) was added cautiously. The mixture was stirred until two phases had formed (approximately 30 min). The phases were separated, and the aqueous phase was extracted with diethyl ether (2 × 500 mL). The combined organic extracts were washed with brine (400 mL), dried (magnesium sulfate), filtered and evaporated under reduced pressure. The residue was purified by flash column chromatography on silica gel (petroleum ether:ethyl acetate 20:1) to afford the alcohol **3** (34.60 g, 84% over 2 steps) as a pale yellow oil. R_f 0.20 (petroleum ether:diethyl ether, 4:1). $[\alpha]_D^{25}$ +23 (*c* = 0.85 in $CHCl_3$). ^{1}H NMR (400 MHz, $CDCl_3$) δ 7.92–7.72 (4H, m), 7.53–7.43 (3H, m), 5.85 (1H, dddd, *J* = 17.3, 10.2, 7.3, 6.5 Hz), 5.15–5.03 (3H, m), 4.85 (1H, d, *J* = 12.0 Hz), 3.92 (1H, dd, *J* = 10.6, 5.3 Hz), 3.72 (2H, m), 3.49 (1H, dd, *J* = 8.2, 8.1 Hz), 3.41 (1H, dd, *J* = 9.3, 8.2 Hz), 3.38–3.32 (1H, m), 3.28 (1H, ddd, *J* = 10.3, 9.5, 5.3 Hz), 2.58 (1H, dd, *J* = 14.6, 7.3 Hz), 2.35 (1H, br s), 2.26 (1H, ddd, *J* = 14.6, 7.3, 7.3 Hz), 1.50 (3H, s), 1.45 (3H, s). ^{13}C NMR (101 MHz, $CDCl_3$) δ 136.1, 134.1, 133.3, 133.0, 128.3, 127.9, 127.7, 126.8, 126.2, 126.0, 125.8, 117.3, 99.2, 82.6, 79.3, 74.9, 74.3, 73.1, 71.5, 62.4, 36.2, 29.2, 19.2. $\nu_{max.}$ (film) 3470, 2895, 1641, 1603, 988, 914, 854, 816, 754, 735 cm^{-1}. HRMS (ESI) calcd. for $C_{23}H_{28}NaO_5$ $[M+Na]^+$ 407.1829, found 407.1814.

Diene 4

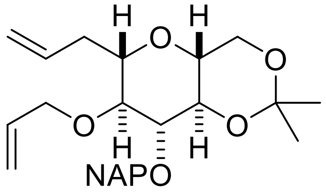

4

To a solution of the alcohol **3** (34.6 g, 90.0 mmol) in anhydrous THF (500 mL), at 0 °C, was added dropwise solution of sodium bis(trimethylsilyl)amide (67.5 mL, 0.135 mol, 2.0 M in THF) and the resulting solution was stirred at 0 °C for 1 h. Allyl bromide (15.6 mL, 0.181 mol) was added slowly followed immediately by tetra-*n*-butylammonium iodide (332 mg, 0.899 mmol). The solution was warmed to room temperature and stirred for 14 h before a saturated aqueous solution of ammonium chloride (200 mL) and diethyl ether (200 mL) were added. The layers were separated, and the aqueous phase was extracted with diethyl ether (2 × 200 mL). The combined organic extracts were washed with brine (200 mL), dried (magnesium sulfate) and filtered. The solvent was removed under reduced pressure and the residue was purified by flash column chromatography on silica gel (petroleum ether:ethyl acetate 50:1 to 15:1) to afford the diene **4** (33.6 g, 88%) as a pale yellow oil.

R_f 0.50 (petroleum ether: diethyl ether, 4:1). $[\alpha]_D^{25}$ −19.3 (*c* = 0.715 in $CHCl_3$). ^1H NMR (400 MHz, $CDCl_3$) δ 7.85–7.81 (4H, m), 7.52–7.46 (3H, m), 5.99–5.80 (2H, m), 5.25 (1H, d, *J* = 17.2 Hz), 5.17 (1H, d, *J* = 10.3 Hz), 5.11 (1H, d, *J* = 16.4), 5.07 (1H, d, *J* = 10.1 Hz), 5.04 (1H, d, *J* = 11.5), 4.90 (1H, d, *J* = 11.5 Hz), 4.43 (1H, dd, *J* = 12.2, 5.7 Hz), 4.14 (1H, dd, *J* = 12.2, 5.8 Hz), 3.92 (1H, dd, *J* = 10.7, 5.3 Hz), 3.75–3.60 (3H, m), 3.42–3.35 (1H, m), 3.28–3.17 (2H, m), 2.57 (1H, br d, *J* = 14.7 Hz), 2.27 (1H, ddd, *J* = 14.7, 7.5, 7.4 Hz), 1.51 (3H, s), 1.44 (3H, s). ^{13}C NMR (101 MHz, $CDCl_3$) δ 136.4, 134.9, 134.4, 133.3, 133.0, 128.0, 127.9, 127.7, 126.5, 126.1, 126.0, 125.7, 117.2, 116.9, 99.1, 83.6, 81.0, 79.2, 75.1, 74.7, 74.2, 71.2, 62.5, 36.2, 29.2, 19.2. $\nu_{max.}$ (film) 2992, 2880, 1643, 1603, 995, 918, 854, 816, 754 cm^{-1}. HRMS (ESI) calcd. for $C_{26}H_{32}NaO_5$ $[M+Na]^+$ 447.2142, found 447.2121.

Bicyclic ether 5[2]

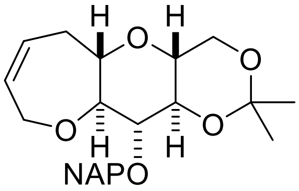

5

To a solution of the diene **4** (33.6 g, 79.1 mmol) in anhydrous and degassed dichloromethane (2.0 L) was added Grubbs II catalyst (2.0 g, 2.5 mmol) and the solution was heated at reflux for 16h. Methanol (20 mL) was added and the solvent was removed under reduced pressure. The residue was purified by flash column chromatography on silica gel (petroleum ether:ethyl acetate 10:1) to afford the bicyclic ether **5** (27.3 g, 87%) as a colourless solid. R_f 0.20 (petroleum ether:diethyl ether, 4:1). Melting point: 128–129 °C (lit.[2] 136–137 °C). $[\alpha]_D^{24}$ −10.7 (*c* = 0.915 in $CHCl_3$) {lit.[2] $[\alpha]_D^{21}$ −12.8 (*c* = 0.99 in $CHCl_3$) }. ^1H NMR (400 MHz, $CDCl_3$) δ 7.86–7.82 (4H, m), 7.55–7.44 (3H, m), 5.90–5.82 (1H, m), 5.80–5.71 (1H, m), 5.02 (1H, d, *J* = 12.1 Hz), 4.97 (1H, d, *J* = 12.1 Hz), 4.33 (1H, dd, *J* = 15.7, 5.8 Hz), 4.04 (1H, br d, *J* = 15.7 Hz), 3.91 (1H, ddd, *J* = 10.7, 5.2, 1.3 Hz), 3.74–3.66 (2H, m), 3.58 (1H, dd, *J* = 8.3, 7.8 Hz), 3.42–3.31 (2H, m), 3.28 (1H, ddd, *J* = 10.2, 10.0, 5.3 Hz), 2.63 (1H, ddd, *J* = 16.5, 8.0, 3.4 Hz), 2.38–2.26 (1H, m), 1.51 (3H, s), 1.44 (3H, s). ^{13}C NMR (101 MHz, $CDCl_3$) δ 136.9, 133.3, 132.9, 131.3, 127.9, 127.85, 127.76, 126.4, 126.1, 125.91, 125.85, 125.6, 99.3, 87.4, 81.8, 77.2, 74.8, 74.2, 70.9, 68.6, 62.4, 34.7, 29.2, 19.2. $\nu_{max.}$ (film) 2992, 2943, 2890, 1603, 950, 857, 817, 755 cm^{-1}. HRMS (ESI) calcd. for $C_{24}H_{28}NaO_5$ $[M+Na]^+$ 419.1829, found 419.1816. Anal. calcd. for $C_{24}H_{28}O_5$: C, 72.70; H, 7.12, found C, 72.69; H, 7.03.

Diol 6[3]

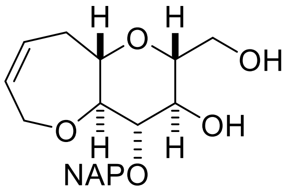

6

To a solution of the acetal **5** (27.3 g, 68.9 mmol) in a mixture of methanol (200 mL) and dichloromethane (200 mL) was added *p*-toluenesulfonic acid hydrate (3.27 g, 17.2 mmol) and the solution was stirred at room temperature for 2 h. The reaction was quenched by the addition of brine (100 mL) and the phases were separated. The aqueous phase was extracted with ethyl acetate (3 × 100 mL) and the combined organic extracts were dried (magnesium sulfate) and filtered. The solvent was removed under reduced pressure and the residue was purified by flash column chromatography on silica gel (petroleum ether:ethyl acetate 1:1) to afford the diol **6** (24.1 g, 98%) as a colourless solid. R_f 0.30 (petroleum ether:ethyl acetate, 1:2). Mp 87–89 °C. $[\alpha]_D^{22}$ −27.2 (*c* = 1.15 in $CHCl_3$) {lit.[3] $[\alpha]_D^{20}$ −25.3 (*c* = 1.74 in $CHCl_3$)}. ^1H NMR (400 MHz, $CDCl_3$) δ 7.88–7.78 (4H, m), 7.52–7.44 (3H, m), 5.95–5.86 (1H, m), 5.81–5.75 (1H, m), 5.16 (1H, d, *J* = 11.9 Hz), 4.89 (1H, d, *J* = 11.9 Hz), 4.29 (1H, dd, *J* = 15.3, 6.1 Hz), 4.05–3.96 (1H, m), 3.84 (1H, br d, *J* = 11.2 Hz), 3.76–3.65 (1H, m), 3.60–3.45 (2H, m), 3.44–3.37 (1H, m), 3.37–3.25 (2H, m), 2.64 (1H, ddd, *J* = 16.1, 8.0, 3.9 Hz), 2.56 (1H, br s), 2.42–2.31 (1H, m), 2.19–2.03 (1H, m). ^{13}C NMR (101 MHz, $CDCl_3$) δ 136.3, 133.3, 133.0, 131.5, 128.4, 127.9, 127.7, 127.2, 126.6, 126.2, 125.9, 125.7, 87.7, 84.8, 78.3, 75.9, 75.2, 70.4, 67.9, 62.9, 34.5. $\nu_{max.}$ (film) 3401, 2879, 2868, 951, 907, 854, 818 cm^{-1}. HRMS (ESI) calcd. for $C_{21}H_{24}NaO_5$ $[M+Na]^+$ 379.1516, found 379.1500.

Triflate 7

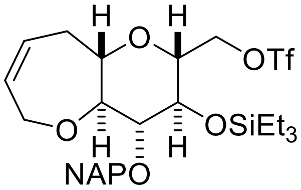

7

To a solution of the diol **6** (20.0 g, 56.1 mmol) in anhydrous dichloromethane (300 mL), at −78 °C, was added 2,6-lutidine (32.5 mL, 281 mmol) followed by slow addition of trifluoromethanesulfonic anhydride (9.89 mL, 58.9 mmol). The solution was stirred at −78 °C for 30 min and triethylsilyl trifluoromethanesulfonate (19.0 mL, 84.0 mmol) was added dropwise. The mixture was then stirred at −78 °C for an additional period of 30 min. The reaction was quenched by addition of a saturated aqueous solution of sodium bicarbonate (100 mL) and the mixture was allowed to warm to room temperature. Diethyl ether (200 mL) was added and the phases were separated. The organic phase was washed with a saturated aqueous solution of copper(II) sulfate (3 × 200 mL) and brine (150 mL), and was then dried (magnesium sulfate) and filtered. The solvent was removed under reduced

pressure and the residue was purified quickly by flash column chromatography on silica gel, (petroleum ether:diethyl ether, 15:1) to afford the triflate **7** (33.1 g, 98%) as a yellow oil that was used immediately in the next reaction.

Alkyne S1

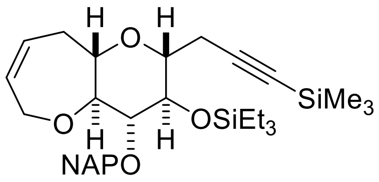

S1

To a solution of trimethylsilylacetylene (27.4 mL, 0.192 mol) in anhydrous THF (300 mL), at −78 °C, was added dropwise solution of *n*-butyllithium (77.0 mL of a 2.5 M solution in hexane, 192 mmol). The solution of the anion was stirred at 0 °C for 30 min and then cooled to −78 °C. A solution of the triflate **7** (33.1 g, 55.0 mmol) in anhydrous THF (50 mL) was then added followed immediately by DMPU (6.9 mL, 58 mmol) in one portion, and the mixture was stirred at −78 °C for 30 min. The dry ice bath was removed and stirring was continued for 1 h before the reaction was quenched by addition of a saturated aqueous solution of ammonium chloride (200 mL). The phases were separated, and the aqueous phase was extracted with diethyl ether (2 × 300 mL). The combined organic extracts were washed with brine (200 mL), dried (magnesium sulfate) and filtered. The solvent was removed under reduced pressure and the residue was purified by flash column chromatography on silica gel (petroleum ether:diethyl ether, 20:1) to afford the alkyne **S1** (28.8 g, 95%). R_f 0.40 (petroleum ether:diethyl ether, 9:1). $[\alpha]_D^{24}$ +45 (*c* = 0.88 in $CHCl_3$). ^{1}H NMR (400 MHz, $CDCl_3$) δ 7.85–7.81 (4H, m), 7.53–7.45 (3H, m), 5.88–5.73 (2H, m), 5.15 (1H, d, *J* = 11.7 Hz), 4.90 (1H, d, *J* = 11.7 Hz), 4.13 (1H, dd, *J* = 15.4, 5.5 Hz), 3.87 (1H, br d, *J* = 15.4 Hz), 3.64 (1H, dd, *J* = 8.9, 8.7 Hz), 3.46 (1H, dd, *J* = 8.4, 8.3 Hz), 3.41 (1H, dd, *J* = 8.9, 8.4 Hz), 3.35–3.24 (2H, m), 2.69 (1H, dd, *J* = 17.1, 3.5 Hz), 2.67 (1H, dd, *J* = 16.2, 3.6 Hz), 2.53 (1H, dd, *J* = 17.1, 5.9 Hz), 2.44–2.32 (1H, m), 0.94 (9H, t, *J* = 7.9 Hz), 0.71–0.63 (6H, m), 0.17 (9H, s). ^{13}C NMR (101 MHz, $CDCl_3$) δ 137.2, 133.4, 132.7, 131.4, 127.8, 127.71, 127.65, 127.3, 125.9, 125.62, 125.59, 125.51, 103.1, 88.5, 86.7, 85.8, 77.6, 75.9, 75.1, 73.2, 67.8, 34.5, 23.4, 7.00, 5.3, 0.1. $\nu_{max.}$ (film) 2955, 2876, 2177, 972, 841, 818 cm^{-1}. HRMS (ESI) calcd. for $C_{32}H_{46}NaO_4Si_2$ $[M+Na]^+$ 573.2827, found 573.2803.

Alcohol 8

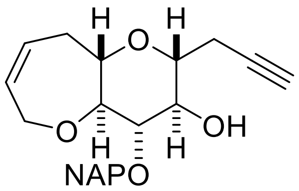

8

To a solution of the protected alkyne **S1** (28.77 g, 52.22 mmol) in anhydrous THF (200 mL), at 0 °C, was added solution of tetra-*n*-butylammonium fluoride (209 mL of a 1 M solution in THF, 209 mmol) over 15 min using an addition funnel. The resulting mixture was stirred for 16 h at room temperature before a saturated aqueous solution of ammonium chloride (200 mL) and ethyl acetate (200 mL) were added. The phases were separated, and the aqueous phase was extracted with ethyl acetate (2 × 200 mL). The combined organic extracts were washed with brine (200 mL), dried (magnesium sulfate) and filtered. The solvent was removed under reduced pressure and the residue was purified by flash column chromatography on silica gel (petroleum ether:ethyl acetate, 4:1) to afford alkyne **8** (18.27 g, 96%) as a colourless solid. R_f 0.23 (petroleum ether:diethyl ether, 2:1). Mp 95–97 °C. $[\alpha]_D^{26}$ −19.9 (*c* = 2.30 in $CHCl_3$). ^1H NMR (400 MHz, $CDCl_3$) δ 7.87–7.82 (4H, m), 7.52–7.47 (3H, m), 5.96–5.88 (1H, m), 5.87–5.78 (1H, m), 5.17 (1H, d, *J* = 11.7 Hz), 4.89 (1H, d, *J* = 11.7 Hz), 4.30 (1H, dd, *J* = 15.3, 6.0 Hz), 4.03 (1H, br d, *J* = 15.3 Hz), 3.54 (1H, app. t, *J* = 8.1 Hz), 3.50 (1H, app. t, *J* = 8.4 Hz), 3.45 (1H, app. t, *J* = 8.0 Hz), 3.39–3.25 (2H, m), 2.74–2.63 (2H, m), 2.55–2.27 (3H, m), 2.00 (1H, s). ^{13}C NMR (101 MHz, $CDCl_3$) δ 136.3, 133.3, 133.0, 131.5, 128.4, 127.9, 127.7, 127.5, 126.6, 126.2, 126.0, 125.8, 87.8, 84.6, 80.4, 76.3, 76.0, 75.2, 72.3, 69.9, 67.8, 34.4, 22.0. $\nu_{max.}$ (film) 3439, 3289, 3053, 2893, 2866, 2118, 1603, 905, 856, 818, 754 cm^{-1}. HRMS (ESI) calcd. for $C_{23}H_{24}NaO_4$ $[M+Na]^+$ 387.1567, found 387.1558.

Diene 9

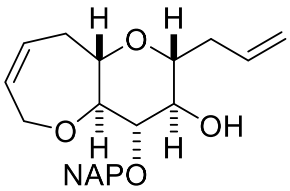

9

To a solution of alkyne **8** (18.27 g, 50.13 mmol) in ethyl acetate (200 mL) was added Lindlar catalyst (1.08 g) followed by quinoline (0.29 mL, 2.4 mmol). A stream of hydrogen gas was passed through the reaction mixture for 15 min. The mixture was stirred at room temperature under static atmosphere of hydrogen for 2 h and then the solution was purged with argon for 20 min. The mixture was filtered through a pad of Celite and the pad was washed with ethyl acetate (200 mL). The filtrate and washings were combined, and the solvent was removed under reduced pressure. The residue was purified by flash column chromatography on silica gel (petroleum ether:ethyl acetate, 3:1) to afford the alkene **9** (16.90 g, 92%) as a colourless solid. R_f 0.58 (petroleum ether:diethyl ether, 1:1). Mp 112−114 °C. $[\alpha]_D^{26}$ −31 (*c* = 0.60 in $CHCl_3$);). ^1H NMR (400 MHz, $CDCl_3$) δ 7.87–7.80 (4H, m), 7.52–7.43 (3H, m), 5.94–5.77 (3H, m), 5.17 (1H, d, *J* = 11.9 Hz), 5.11 (1H, ddt, *J* = 17.2, 1.7, 1.2 Hz), 5.05 (1H, ddt, *J* = 10.1, 1.7, 1.2 Hz), 4.89 (1H, d, *J* = 11.9 Hz), 4.30 (1H, dd, *J* = 15.2, 6.0 Hz), 4.02 (1H, app. dq, *J* = 15.2, 2.9 Hz), 3.48 (1H, app. t, *J* = 8.5 Hz), 3.42 (1H, app. t, *J* = 8.7 Hz), 3.37 (1H, ddd, *J* = 9.2, 8.6, 2.2 Hz), 3.33–3.15 (2H, m), 2.64 (1H, ddd, *J* = 15.9, 8.0, 3.9 Hz), 2.59–2.51 (1H, m), 2.43–2.32 (1H, m), 2.28–2.11 (2H, m). ^{13}C NMR (101 MHz, $CDCl_3$) δ 136.5, 134.7, 133.5, 133.2,

131.7, 128.6, 128.1, 127.88, 127.87, 126.8, 126.3, 126.1, 125.9, 117.1, 88.3, 85.1, 78.2, 75.9, 75.4, 73.1, 67.9, 36.3, 34.7. $\nu_{max.}$ (film) 3455, 3053, 3024, 2893, 2859, 1640, 1602, 818 cm^{-1}. HRMS (ESI) cald. for $C_{23}H_{26}NaO_4$ $[M+Na]^+$ 389.1723, found 389.1714.

Enol ether S2

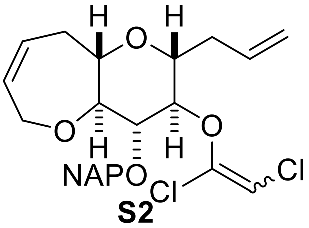

S2

To a suspension of potassium hydride (9.22 g, from 30.7 g of a 30% dispersion in mineral oil, 0.230 mol) in anhydrous THF (500 mL), at 0 °C, was added slowly solution of the diene **9** (16.90 g, 46.12 mmol) in anhydrous THF (50 mL) and the mixture was stirred at room temperature for 1 h. Then trichloroethylene (8.3 mL, 92 mmol) was added at −78 °C, the solution was warmed to room temperature and stirring was continued for 2 h. The reaction was quenched cautiously by dropwise addition of methanol (60 mL) until gas evolution ceased. The mixture was diluted with a saturated aqueous solution of ammonium chloride (200 mL) and diethyl ether (200 mL) and the phases were separated. The aqueous phase was extracted with diethyl ether (200 mL) and the combined organic extracts were washed with brine (200 mL), dried (magnesium sulfate) and filtered. The solvent was removed under reduced pressure and the residue was purified by flash column chromatography on silica gel (petroleum ether:diethyl ether, 20:1) to afford an isomeric mixture of the dichloroenol ether **S2** (17.66 g, 83%) as a yellow oil which was used immediately in the next reaction. R_f 0.38 (petroleum ether:diethyl ether, 9:1).

Alkynyl ether 10

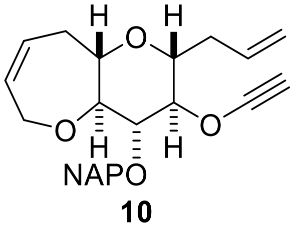

10

To a solution of the dichloroenol ether **S2** (17.66 g, 38.28 mmol) in anhydrous diethyl ether (300 mL), at −78 °C, was added dropwise solution of *n*-butyllithium (91.9 mL of a 2.5 M solution in hexane, 0.23 mol) and the mixture was stirred at −78 °C for 4 h. The reaction was quenched by dropwise addition of methanol (60 mL) at −78 °C and the mixture was warmed to room temperature before a saturated aqueous solution of ammonium chloride (200 mL) was added. The phases were separated, and the aqueous phase was extracted with diethyl ether (200 mL). The combined extracts were washed with brine (200 mL), dried (magnesium sulfate) and filtered. The solvent was removed under reduced pressure and the residue was purified by flash column chromatography on silica gel

(petroleum ether:diethyl ether, 10:1) to afford the alkyne **10** (12.85 g, 86%) as a yellow oil. R_f 0.63 (petroleum ether:diethyl ether, 4:1). $[\alpha]_D^{27}$ −13 (*c* = 0.87 in $CHCl_3$). ^{1}H NMR (400 MHz, $CDCl_3$) δ 7.90–7.80 (4H, m), 7.59 (1H, dd, *J* = 8.5, 1.6 Hz), 7.50–7.42 (2H, m), 5.94–5.76 (3H, m), 5.23–5.09 (3H, m), 5.05 (2H, s), 4.31 (1H, dd, *J* = 15.4, 6.0 Hz), 4.03 (1H, app. dq, *J* = 15.4, 2.9 Hz), 3.90–3.75 (2H, m), 3.55 (1H, ddd, *J* = 9.4, 7.4, 3.2 Hz), 3.42 (1H, app. t, *J* = 8.8 Hz), 3.22 (1H, app. td, *J* = 9.9, 4.0 Hz), 2.69–2.59 (1H, ddd, *J* = 16.1, 8.0, 4.0 Hz), 2.39–2.28 (2H, m), 1.62 (1H, s). ^{13}C NMR (101 MHz, $CDCl_3$) δ 136.3, 133.5, 133.4, 133.2, 131.7, 128.09, 128.07, 127.8, 127.2, 126.8, 126.4, 126.0, 125.9, 118.2, 89.1, 88.0, 87.4, 81.4, 76.8, 75.9, 75.7, 68.1, 35.8, 34.4, 28.2. $\nu_{max.}$ (film) 3316, 3057, 3024, 2940, 2862, 2153, 1740, 1643, 1603, 990, 916, 854, 818 754 cm^{-1}. HRMS (ESI) cald. for $C_{25}H_{26}NaO_4$ $[M+Na]^+$ 413.1723, found 413.1714.

Ester 12

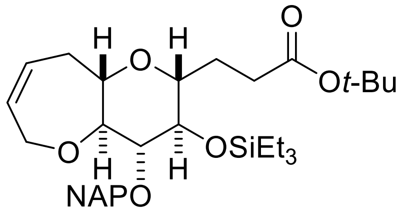

12

To a solution of lithium diisopropylamide (47.5 mL of a 2.0 M solution in a mixture of THF / heptane / ethylbenzene, 95 mmol) in anhydrous THF (200 mL) at 0 °C, was added DMPU (11.5 mL, 95.1 mmol). The resulting mixture was stirred for 15 min at 0 °C and then cooled down to −78 °C. Neat *t*-butyl acetate (12.7 mL, 94.7 mmol) was added dropwise and the solution was stirred at −78 °C for 30 min. A solution of the triflate **7** (16.3 g, 27.0 mmol) in anhydrous THF (70 mL) was added slowly and stirring was continued for an additional 30 min. The reaction was quenched by the addition of a saturated aqueous solution of ammonium chloride (100 mL) at −78 °C and the mixture was warmed up to room temperature. The phases were separated, and the aqueous phase was extracted with diethyl ether (2 × 150 mL). The combined organic extracts were washed with water (4 × 50 mL) and brine (100 mL), and then dried (magnesium sulfate) and filtered. The volatiles were removed under reduced pressure and the residue was purified by flash column chromatography on silica gel (petroleum ether:diethyl ether, 20:1) to afford the ester **12** (15.0 g, 97%) as a pale-yellow oil. Alternatively, the resulting crude ester can be used for the next reaction without further purification. R_f 0.63 (petroleum ether:diethyl ether, 4:1); $[\alpha]_D^{24}$ +21.1 (*c* = 1.43 in $CHCl_3$). ^{1}H NMR (500 MHz, $CDCl_3$) δ 7.84–7.81 (4H, m), 7.51–7.45 (3H, m), 5.86–5.80 (1H, m), 5.80–5.74 (1H, m), 5.12 (1H, d, *J* = 11.8 Hz), 4.90 (1H, d, *J* = 11.8 Hz), 4.11 (1H, dd, *J* = 15.3, 5.8 Hz), 3.84 (1H, app. dq, *J* = 15.3, 2.8 Hz), 3.44 (1H, dd, *J* = 8.3, 8.2 Hz), 3.40 (1H, dd, *J* = 8.3, 8.6 Hz), 3.38 (1H, dd, *J* = 8.2, 8.9 Hz), 3.22–3.13 (2H, m), 2.62 (1H, ddd, *J* = 16.1, 7.9, 3.9 Hz), 2.44 (1H, ddd, *J* = 15.7, 9.6, 5.4 Hz), 2.34–2.25 (2H, m), 2.16 (1H, dddd, *J* = 14.0, 9.4, 6.7, 2.6 Hz), 1.65–1.55 (1H, m), 1.45 (9H, s), 0.93 (9H, t, *J* = 7.9 Hz), 0.67–0.61 (6H, m). ^{13}C NMR (126 MHz, $CDCl_3$) δ 172.8, 137.3, 133.3, 132.7, 131.5,

127.8, 127.7, 127.6, 127.4, 125.9, 125.6, 125.52, 125.48, 88.9, 85.8, 80.0, 78.9, 75.8, 75.04, 75.00, 67.7, 34.6, 32.0, 28.1, 27.5, 7.0, 5.3. $\nu_{max.}$ (film) 2953, 2875, 1729, 1456, 1416, 1391, 1366, 1254, 1151, 1092, 1007, 952, 893, 850, 818, 737, 638, 618 cm^{-1}. HRMS (ESI) calcd. for $C_{33}H_{48}NaO_6Si$ $[M+Na]^+$ 591.3118, found 591.3099.

Diol 13

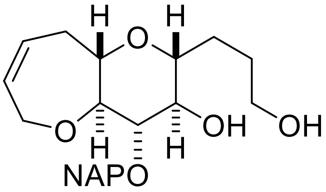

13

To a suspension of lithium aluminium hydride (1.0 g, 26 mmol) in anhydrous THF (230 mL), at 0 °C, was added dropwise solution of the ester (15.0 g, 26.4 mmol) in anhydrous THF (50 mL). After addition was complete, the mixture was warmed to room temperature and stirred for 1 h. The reaction was quenched by addition of a saturated aqueous solution of Rochelle salt (100 mL) cautiously at 0 °C. The mixture was diluted with ethyl acetate (100 mL) and then stirred vigorously at room temperature until a biphasic mixture formed (1 h). The phases were separated, and the aqueous phase was extracted with ethyl acetate (2 × 100 mL). The combined organic extracts were washed with brine (200 mL), and then dried (magnesium sulfate), and filtered. The solvent was evaporated under reduced pressure and the resulting crude alcohol was used in the next reaction without further purification.

To a solution of the crude alcohol in THF (140 mL) was added a solution of tetra-*n*-butylammonium fluoride (53 mL of a 1 M solution in THF, 53 mmol) dropwise at room temperature and the resulting solution was stirred for 1 h. The reaction was quenched with by the addition of water (100 mL) and the resulting mixture was diluted with ethyl acetate (100 mL). The phases were separated and the aqueous was extracted with ethyl acetate (3 × 100 mL). The combined organic extracts were washed with brine (100 mL), then dried (magnesium sulfate) and filtered. The solvent was evaporated under reduced pressure and the residue was purified by flash column chromatography on silica gel (petroleum ether:ethyl acetate, 1:1 to 1:3) to afford the diol **13** (8.7 g, 86% over 2 steps) as a colourless solid. R_f 0.13 (petroleum ether:ethyl acetate, 1:1). Mp 91–92 °C. $[\alpha]_D^{17}$ −20.2 (*c* = 1.25 in $CHCl_3$). ^1H NMR (500 MHz, $CDCl_3$) δ 7.86–7.81 (4H, m), 7.51–7.46 (3H, m), 5.91 (1H, ddt, *J* = 11.2, 6.4, 3.3 Hz), 5.80 (1H, ddt, *J* = 11.2, 8.1, 3.0 Hz), 5.15 (1H, d, *J* = 11.9 Hz), 4.88 (1H, d, *J* = 11.9 Hz), 4.29 (1H, dd, *J* = 15.3, 6.1 Hz), 4.01 (1H, app. dq, *J* = 15.3, 2.9 Hz), 3.66–3.55 (2H, m), 3.46 (1H, app. t, *J* = 8.6 Hz), 3.41 (1H, app. t, *J* = 8.6 Hz), 3.31 (1H, dd, *J* = 9.0, 8.8 Hz), 3.28–3.18 (2H, m), 2.66–2.58 (2H, m), 2.41–2.32 (2H, m), 1.97 (1H, app. dtd, *J* = 14.3, 7.2, 2.5 Hz), 1.75–1.62 (2H, m), 1.46 (1H, app. ddt, *J* = 14.3, 8.6, 7.2 Hz). ^{13}C NMR (126 MHz, $CDCl_3$) δ 136.2, 133.3, 133.0, 131.6, 128.4, 127.9, 127.7, 127.3, 126.6, 126.2, 125.9, 125.8, 87.9, 84.8, 78.6, 75.8, 75.2, 73.1,

67.7, 62.7, 34.4, 28.7, 28.4. $\nu_{max.}$ (film) 3372, 3360, 2921, 2855, 962, 894, 855, 817, 752, 738, 705 cm^{-1}. HRMS (ESI) calcd. for $C_{23}H_{28}NaO_5$ [M+Na]$^+$ 407.1829, found 407.1829.

Lactone 14

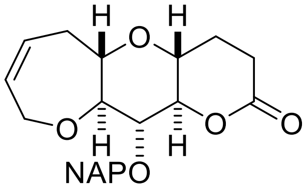

14

To a solution of diol **13** (8.7 g, 23 mmol) in anhydrous dichloromethane (115 mL) was added bis(acetoxy)iodobenzene (18.3 g, 56.8 mmol) followed by TEMPO (710 mg, 4.54 mmol) and the resulting solution was stirred at room temperature for 3 h. The reaction was quenched by addition of a saturated aqueous solution of sodium thiosulfate (100 mL) and the biphasic mixture was stirred vigorously until both layers were clear. The phases were separated, and the aqueous phase was extracted with dichloromethane (2 × 100 mL). The combined extracts were washed with a saturated aqueous solution of sodium bicarbonate (3 × 75 mL), dried (magnesium sulfate) and filtered. The solvent was evaporated under reduced pressure and the residue was purified by flash column chromatography on silica gel (petroleum ether:ethyl acetate, 4:1 to 1:1) to afford the lactone **14** (7.08 g, 82%) as a beige solid. R_f 0.56 (petroleum ether:ethyl acetate, 1:1). Mp 128–130 °C. $[\alpha]_D^{16}$ −44.5 (*c* = 1.00 in $CHCl_3$). ^{1}H NMR (400 MHz, $CDCl_3$) δ 7.89–7.81 (4H, m), 7.57 (1H, dd, *J* = 8.4, 1.6 Hz), 7.49–7.42 (2H, m), 5.88 (1H, app. ddt, *J* = 11.5, 5.9, 3.2 Hz), 5.77 (1H, app. ddt, *J* = 11.5, 7.9, 2.9 Hz), 5.09 (1H, d, *J* = 11.5 Hz), 5.01 (1H, d, *J* = 11.5 Hz), 4.32 (1H, dd, *J* = 15.6, 5.9 Hz), 4.11–4.00 (2H, m), 3.69 (1H, dd, *J* = 8.8, 8.6 Hz), 3.55–3.42 (1H, m), 3.41 (1H, dd, *J* = 8.9, 8.5 Hz), 3.38–3.29 (1H, m), 2.84–2.73 (1H, m), 2.66 (1H, *J* = 16.4, 8.0, 4.0 Hz), 2.65–2.54 (1H, m), 2.40–2.28 (1H, m), 2.26–2.12 (1H, m) 1.93–1.80 (1H, m). ^{13}C NMR (101 MHz, $CDCl_3$) δ 170.1, 136.0, 133.2, 133.0, 131.3, 127.9, 127.6, 126.5, 126.3, 126.0, 125.9, 125.7, 86.9, 81.7, 80.6, 76.7 (2C), 75.4, 71.5, 68.3, 34.4, 27.6, 24.4. $\nu_{max.}$ (film) 3059, 2938, 2886, 1742, 945, 818, 733, 702 cm^{-1}. HRMS (ESI) calcd. for $C_{23}H_{24}NaO_5$ [M+Na]$^+$ 403.1516, found 403.1519.

A–C Triene 11

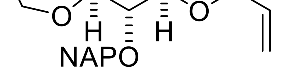

11

Method A – via an enol triflate

To a solution of the lactone **14** (6.0 g, 16 mmol) in anhydrous THF (130 mL), at −78 °C, was added DMPU (2.3 mL, 19 mmol) followed by dropwise addition of potassium bis(trimethylsilyl)amide (37.8

mL of a 0.5 M solution in toluene, 18.9 mmol). The mixture was stirred at −78 °C for 15 min before *N*-phenyl-bis(trifluoromethanesulfonimide (7.3 g, 20 mmol) in anhydrous THF (20 mL) was added dropwise. The mixture was stirred at −78 °C for a further 1 h and then the reaction was quenched by the addition of pH 7 phosphate buffer (100 mL). The mixture was warmed to room temperature and diluted with diethyl ether (100 mL). The phases were separated, and the aqueous phase was extracted with diethyl ether (100 mL). The combined organic extracts were washed with brine (100 mL), dried (magnesium sulfate) and filtered. Solvent was removed under reduced pressure to afford the crude enol triflate, which was used in the next reaction without further purification.

A solution of the enol triflate in anhydrous THF (150 mL) was sparged with argon for 30 min and tri-*n*-butyl(vinyl)tin (6.9 mL, 24 mmol) was added. Lithium chloride (2.0 g, 47 mmol) and tetrakis(triphenylphosphine)palladium(0) (1.81 g, 1.57 mmol) were added and the solution was heated at reflux for 12 h. The mixture was cooled to room temperature and diethyl ether (100 mL) was added followed by a 20% aqueous solution of potassium fluoride (100 mL). The mixture was then stirred vigorously for 30 min. The phases were separated, and the aqueous phase was extracted with diethyl ether (100 mL). The combined organic extracts were washed with brine (100 mL), dried (magnesium sulfate) and filtered. The solvent was removed under reduced pressure and the residue was purified by flash column chromatography on silica gel (petroleum ether:ethyl acetate 50:1 to 10:1) to afford the tricyclic triene **11** (4.9 g, 80% over 2 steps) as a colourless solid.

Method B – RCM of an alkynyl ether

To a solution of alkyne **10** (4.68 g, 12.0 mmol) in anhydrous and degassed toluene was added Hoveyda-Grubbs II catalyst (376 mg, 0.600 mmol). The solution was sparged with ethene for 15 minutes (colour yellow/orange) and then stirred for 3 h at 70 °C under a static atmosphere of ethene. The reaction was allowed to cool to room temperature and methanol (10 mL) was added. The mixture was purged with argon for 10 min and the solvent was removed under reduced pressure. The solvent was removed under reduced pressure and the residue was purified by flash column chromatography on silica gel (petroleum ether:ethyl acetate, 10:1) to afford the tricyclic triene **11** (2.01g, 43%) a colourless solid. R_f 0.27 (petroleum ether:diethyl ether, 9:1). Mp 124–126 °C. $[\alpha]_D^{29}$ −68.5 (*c* = 0.47 in $CHCl_3$). ^1H NMR (500 MHz, $CDCl_3$) δ 7.91 (1H, s), 7.87–7.82 (3H, m), 7.62 (1H, dd, *J* = 8.5, 1.8 Hz), 7.50–7.43 (2H, m), 6.12 (1H, dd, *J* = 17.1, 10.8 Hz), 5.92 (1H, dddd, *J* = 11.2, 6.3, 3.3, 3.0 Hz), 5.84–5.78 (1H, m), 5.57 (1H, d, *J* = 17.1 Hz), 5.17 (1H, d, *J* = 11.8 Hz), 5.11 (1H, d *J* = 10.8 Hz), 5.09 (1H, d, *J* = 11.8 Hz), 4.81 (1H, dd, *J* = 5.6, 2.7 Hz), 4.37 (1H, dd, *J* = 15.4, 5.9 Hz), 4.10 (1H, app. dq, *J* = 15.4, 2.9 Hz), 3.77 (1H, app. t, *J* = 8.8 Hz), 3.68 (1H, dd, *J* = 9.5, 9.2 Hz), 3.54 (1H, ddd, *J* = 9.7, 9.5, 6.2 Hz), 3.49 (1H, app. t, *J* = 8.8 Hz), 3.38 (1H, ddd, *J* = 9.6, 9.4, 4.0 Hz), 2.69 (1H, ddd, *J* = 16.2, 8.0, 4.0 Hz), 2.46 (1H, ddd, *J* = 17.3, 6.2, 5.6 Hz), 2.43–2.35 (1H, m), 2.20 (1H, ddd, *J* = 17.3, 9.8, 2.7 Hz). ^{13}C NMR (126 MHz, $CDCl_3$) δ 150.5, 136.6, 133.3, 133.0, 131.5, 131.3, 127.9(2C), 127.6, 126.8, 126.3, 126.1, 125.8, 125.6, 113.2, 100.3, 87.1, 82.3, 78.7, 76.2, 75.3, 71.3, 68.2, 34.6, 27.9. $\nu_{max.}$ (film) 3053, 3024, 2899, 2851, 1657, 1601, 907, 854, 816 cm^{-1}. HRMS (EI) calcd. for

$C_{25}H_{26}O_4$ $[M]^+$ 390.1831, found 390.1833. Anal. calcd. for $C_{25}H_{26}O_4$: C, 76.90; H, 6.71, found C, 76.71; H, 6.79.

A–C Alcohols 15a,b

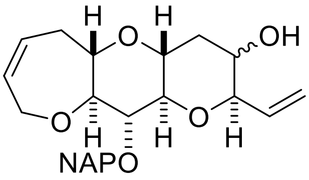

15a,**b**

To a solution of the enol ether **11** (4.0 g, 10 mmol) in dichloromethane (100 mL) and methanol (25 mL), at 0 °C, was added *m*-chloroperoxybenzoic acid (2.65 g, 15.4 mmol) and the solution was stirred until complete consumption of starting material was observed by TLC analysis (approximately 1 h). The reaction was quenched by the addition of a saturated aqueous solution of sodium thiosulfate (50 mL) and the mixture was stirred for a further 30 min. The phases were separated, and the aqueous phase was extracted with dichloromethane (2 × 50 mL). The combined organic extracts were washed with an aqueous solution of sodium bicarbonate (2 × 50 mL, prepared from 25 mL a saturated aqueous solution of sodium bicarbonate and 25 mL of water) and water (30 mL), then dried (magnesium sulfate) and filtered. The solvent was removed under reduced pressure and the crude methoxy acetal was used at the next step without purification.

The crude acetal was dissolved in anhydrous dichloromethane (100 mL) and triethylsilane (1.63 mL, 10.2 mmol) was added. The mixture was cooled 0 °C and boron trifluoride etherate (1.26 mL, 10.2 mmol) was added dropwise. The resulting solution was stirred at 0 °C for 1 h and then the reaction was quenched by the addition of a saturated aqueous solution of sodium bicarbonate (50 mL). The mixture was stirred for 10 minutes before the phases were separated. The aqueous phase was extracted with dichloromethane (100 mL) and the combined organic extracts were dried (magnesium sulfate) and filtered. The solvent was removed under reduced pressure and the residue was purified by flash column chromatography on silica gel (petroleum ether:ethyl acetate 2:1) to deliver the required alcohol (1.84 g, 44% over 2 steps) as a mixture of diastereomers **15a** and **15b**.

A–C Alcohol 15a

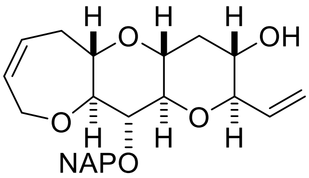

15a

The diastereomeric mixture of alcohols **15a** and **15b** (1.84 g, 4.50 mmol) was dissolved in anhydrous dichloromethane (45 mL) and sodium bicarbonate (1.50 g, 17.9 mmol) was added in one portion and the mixture was cooled to 0 °C. Dess-Martin periodinane (2.86 g, 6.74 mmol) was added and the mixture was stirred at room temperature for 2 h. The reaction was quenched by the addition of a

saturated aqueous solution of sodium thiosulfate (20 mL) and the mixture was stirred for a further 30 min. Diethyl ether (50 mL) was added and the phases were separated. The organic phase was washed successively with a saturated aqueous solution of sodium bicarbonate (30 mL) and water (30 mL), then dried (magnesium sulfate) and filtered. The solvent was removed under reduced pressure to give the ketone **16**, which was used for the next step without purification.

To a mixture of the crude ketone **16** in methanol (40 mL) and dichloromethane (40 mL) was added sodium borohydride (222 mg, 5.87 mmol) at −78 °C and the mixture was stirred at room temperature for 1 h. The reaction was quenched with addition of a saturated aqueous solution of ammonium chloride (20 mL) and the mixture was diluted with ethyl acetate (20 mL). The phases were separated, and the aqueous phase was extracted with ethyl acetate (3 × 20 mL). The combined organic phases were washed with brine (20 mL), dried (magnesium sulfate) and filtered. The solvent was removed under reduced pressure and the residue was purified by flash column chromatography on silica gel (petroleum ether:ethyl acetate 2:1) to afford the alcohol **15a** (1.20 g, 65% over 2 steps) as a colourless viscous oil. The product was dissolved in a mixture of petroleum ether and dichloromethane (3:1). Subsequent evaporation of the solvent under reduced delivered the alcohol **15a** as a colourless solid. R_f 0.46 (petroleum ether:ethyl acetate, 1:1). Mp 88–90 °C. $[\alpha]_D^{23}$ +0.28 (*c* = 1.85 in $CHCl_3$). ^1H NMR (400 MHz, $CDCl_3$) δ 7.86 (1H, br s), 7.84–7.75 (3H, m), 7.55 (1H, dd, *J* = 8.5, 1.7 Hz), 7.48–7.41 (2H, m), 5.93 (1H, ddd, *J* = 17.2, 10.6, 6.6 Hz), 5.90–5.84 (1H, m), 5.82–5.74 (1H, m), 5.46 (1H, ddd, *J* = 17.2, 1.7, 1.3 Hz), 5.37 (1H, ddd, *J* = 10.6, 1.7, 1.0 Hz), 5.06 (1H, dd, *J* = 12.1, 0.8 Hz), 4.99 (1H, dd, *J* = 12.1, 0.8 Hz), 4.32 (1H, dd, *J* = 15.6, 5.9 Hz), 4.05 (1H, app. dq, *J* = 15.6, 2.7 Hz), 3.62 (1H, dddd, *J* = 9.1, 6.6, 1.3, 1.0 Hz), 3.55 (1H, dd, *J* = 8.8, 8.4 Hz), 3.40 (1H, dd, *J* = 9.2, 8.4 Hz), 3.43–3.36 (1H, m), 3.30 (1H, ddd, *J* = 9.8, 9.2, 4.0 Hz), 3.22 (1H, dd, *J* = 9.4, 8.8 Hz), 3.17 (1H, ddd, *J* = 10.9, 9.4, 4.2 Hz), 2.66 (1H, ddd, *J* = 16.2, 7.9, 4.0 Hz), 2.43 (1H, dd, *J* = 11.6, 4.4, 4.2 Hz), 2.36 (1H, dddd, *J* = 16.2, 9.8, 3.0, 2.5 Hz), 1.82 (1H, brs), 1.53 (1H, ddd, *J* = 11.6, 11.1, 10.9 Hz). ^{13}C NMR (101 MHz, $CDCl_3$) δ 136.7, 135.4, 133.3, 132.9, 131.4, 127.9, 127.8, 127.6, 126.7, 126.3, 126.1, 125.8, 125.6, 118.8, 87.4, 83.0, 82.0, 81.1, 76.8, 75.0, 73.2, 68.8, 68.4, 37.6, 34.6. $\nu_{max.}$ (film) 3449, 3024, 2934, 2893, 2870, 1643, 1603, 928, 856, 818, 735 cm^{-1}. HRMS (ESI) calcd. for $C_{25}H_{28}NaO_5$ $[M+Na]^+$ 431.1829, found 431.1818.

A–C Enone 17

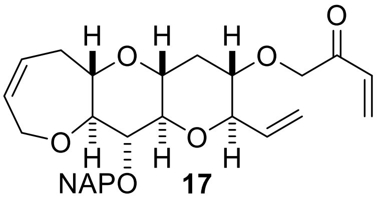

17

To a suspension of sodium hydride (280 mg, from 467 mg of a 60% dispersion in mineral oil, 11.7 mmol) in anhydrous THF (40 mL) at 0 °C was added dropwise, a solution of the alcohol **15a** (1.20 g, 2.92 mmol) in anhydrous THF (10 mL) and the mixture was stirred at room temperature for 20 min.

1-Chloro-3-(triphenylphosphoranylidene)-2-propanone[4] (1.34 g, 3.80 mmol) was added followed by tetra-*n*-butylammonium iodide (107 mg, 0.290 mmol) and the resulting mixture was heated at reflux for 12 h. The reaction was quenched cautiously by the addition of water (20 mL) and the resulting mixture was diluted with ethyl acetate (20 mL). The phases were separated, and the aqueous phase was extracted with ethyl acetate (2 × 20 mL). The combined organic extracts were washed with brine (20 mL), dried (magnesium sulfate) and filtered. The solvent was removed under reduced pressure to afford the crude product.

To a solution of the crude phosphonium ylide in THF (50 mL) was added pH 7 phosphate buffer solution (50 mL) followed by solution of formaldehyde (4.8 mL of a 37% w/w solution in water, 58 mmol). The resulting mixture was stirred at room temperature for 2 h and then diluted with diethyl ether (50 mL). The phases were separated, and the aqueous phase was extracted with diethyl ether (2 × 50 mL). The combined organic extracts were washed with water (2 × 50 mL) and brine (50 mL), then dried (magnesium sulfate) and filtered. The solvent was evaporated under reduced pressure and the residue was purified by flash column chromatography on silica gel (petroleum ether:ethyl acetate, 3:1) to afford the enone **17** (1.04 g, 75% over 2 steps) as a colourless solid. R_f 0.37 (petroleum ether:diethyl ether, 1:1). $[\alpha]_D^{22}$ +25 (*c* = 0.55 in $CHCl_3$). ^1H NMR (400 MHz, $CDCl_3$) δ 7.86–7.79 (4H, m), 7.55 (1H, dd, *J* = 8.4, 1.7 Hz), 7.47–7.43 (2H, m), 6.55 (1H, dd, *J* = 17.6, 10.7 Hz), 6.34 (1H, dd, *J* = 17.6, 1.4 Hz), 6.06 (1H, ddd, *J* = 17.3, 10.7, 5.4 Hz), 5.91–5.83 (1H, m), 5.83 (1H, dd, *J* = 10.7, 1.4 Hz), 5.83–5.72 (1H, m), 5.48 (1H, app. dt, *J* = 17.3, 1.8, 1.4 Hz), 5.30 (1H, app. dt, *J* = 10.7, 1.8, 1.4 Hz), 5.06 (1H, d, *J* = 12.0, Hz), 4.99 (1H, d, *J* = 12.0, Hz), 4.36–4.29 (1H, m), 4.32 (1H, d, *J* = 16.6 Hz), 4.26 (1H, d, *J* = 16.6 Hz), 4.06 (1H, app. dq , *J* = 15.6, 2.9 Hz), 3.81 (1H, dddd, *J* = 9.3, 5.4, 1.4 Hz), 3.55 (1H, dd, *J* = 9.0, 8.3 Hz), 3.39 (1H, dd, *J* = 9.2, 8.3 Hz), 3.29 (1H, ddd, *J* = 9.7, 9.2, 3.9 Hz), 3.25 (1H, dd, *J* = 9.3, 9.0 Hz), 3.20 (1H, ddd, *J* = 10.3, 9.3, 4.4 Hz), 3.13 (1H, ddd, *J* = 11.4, 9.3, 4.2 Hz), 2.64 (1H, ddd, *J* = 16.3, 7.9, 4.0 Hz), 2.57 (1H, ddd, *J* = 11.6, 4.4, 4.2 Hz), 2.41–2.30 (1H, m), 1.56 (1H, ddd, *J* = 11.6, 11.4, 10.3 Hz). ^{13}C NMR (101 MHz, $CDCl_3$) δ 196.7, 136.6, 135.3, 133.3, 133.0, 132.2, 131.4, 129.4, 127.9, 127.8, 127.62, 127.60, 126.6, 126.3, 126.1, 125.8, 125.6, 117.3, 87.3, 81.9, 81.1, 80.4, 78.0, 76.9, 75.1, 73.7, 73.0, 68.4, 35.4, 34.6. $\nu_{max.}$ (film): 2933, 2872, 1702, 1616, 990, 856, 819 cm^{-1}. HRMS (ESI) calcd. for $C_{29}H_{32}NaO_6$, $[M+Na]^+$ 499.2091, found 499.2072.

A–D Enone 18

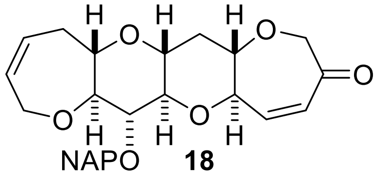

18

To a solution of the triene **17** (1.04 g, 2.19 mmol) in anhydrous and degassed toluene (365 mL), at 60 °C, was added solution of Grubbs II catalyst (186 mg, 0.218 mmol) in anhydrous and degassed toluene (37 mL) dropwise over 4 h using a syringe pump. After the addition was complete, the mixture

was stirred at 60 °C for an additional 1 h. The mixture was allowed to cool to room temperature and methanol (10 mL) was added. The solvent was evaporated under reduced pressure and the residue was purified by flash column chromatography on silica gel (petroleum ether:ethyl acetate, 3:1) to afford the tetracyclic enone **18** (668 mg, 68%) as a colourless solid. R_f 0.30 (petroleum ether:diethyl ether, 1:1). Mp 107–109 °C. $[\alpha]_D^{21}$ −53.3 (*c* = 1.22 in $CHCl_3$). ^1H NMR (400 MHz, $CDCl_3$) δ 7.86–7.81 (4H, m), 7.55 (1H, dd, *J* = 8.5, 1.7 Hz), 7.49–7.35 (2H, m), 6.56 (1H, dd, *J* = 12.8, 2.3 Hz), 6.03 (1H, dd, *J* = 12.8, 2.6 Hz), 5.88 (1H, app. ddt, *J* = 11.1, 6.0, 3.2 Hz), 5.78 (1H, 1H, app. ddt, *J* = 11.1, 7.9, 2.9 Hz), 5.02 (2H, s), 4.39 (1H, d, *J* = 18.2 Hz), 4.32 (1H, dd, *J* = 15.6, 6.0 Hz), 4.25 (1H, d, *J* = 18.2 Hz), 4.05 (1H, app. dq, *J* = 15.6, 2.5 Hz), 4.02 (1H, ddd, *J* = 9.0, 2.6, 2.3 Hz), 3.55 (1H, app. t, *J* = 8.8 Hz), 3.47 (1H, ddd, *J* = 11.2, 9.0, 4.7 Hz), 3.41 (1H, dd, *J* = 8.8, 8.4 Hz), 3.31 (1H, ddd, *J* = 10.2, 8.8, 4.0 Hz), 3.26 (1H, dd, *J* = 9.3, 9.1 Hz), 3.17 (1H, ddd, *J* = 11.5, 9.3, 4.3 Hz), 2.67 (1H, ddd, *J* = 16.2, 7.9, 4.0 Hz), 2.47 (1H, ddd, *J* = 11.7, 4.7, 4.3 Hz), 2.41–2.29 (1H, m), 1.66 (1H, ddd, *J* = 11.7, 11.5, 11.2 Hz). ^{13}C NMR (101 MHz, $CDCl_3$) δ 201.1, 136.5, 133.3, 133.0, 131.3, 128.0, 127.9, 127.8, 127.7, 126.5, 126.3, 126.0, 125.9, 125.7, 87.4, 82.0, 81.6, 80.3, 78.2, 77.3, 76.9, 75.3, 73.0, 68.4, 36.2, 34.5. $\nu_{max.}$ (film) 3025, 2935, 2875, 1668, 1603, 977, 855, 819, 749 cm^{-1}. HRMS (ESI) calcd. for $C_{27}H_{28}NaO_6$ $[M+Na]^+$ 471.1778, found 471.1763.

A–E Enol carbonate 19

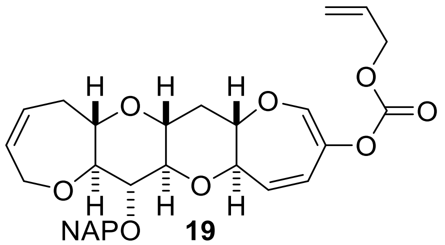

19

To a stirred solution of enone **18** (120 mg, 0.268 mmol) in THF (20 mL) at −78 °C was added dropwise allyl chloroformate (43 μL, 0.40 mmol) and the solution was stirred for 10 min. Sodium bis(trimethylsilyl)amide (0.20 mL of a 1.95 M solution in THF, 0.39 mmol) was added dropwise and the resulting solution stirred at −78 °C for 2 h. The reaction was quenched by addition of an aqueous solution of potassium dihydrogenphosphate (5 mL). The aqueous phase was extracted with ethyl acetate (2 × 10 mL) and the combined organic extracts were washed with brine (5 mL), dried (magnesium sulfate) and filtered. The solvent was evaporated under reduced pressure and the crude product was purified by flash column chromatography on silica gel (petroleum ether:ethyl acetate, 4:1) to afford the enol carbonate **19** (116 mg, 81%) as a colourless solid. R_f = 0.58 (petroleum ether:diethyl ether, 1:1). Mp 149–151 °C. $[\alpha]_D^{21}$ −20 (*c* = 0.89 in $CHCl_3$). ^1H NMR (500 MHz, $CDCl_3$) δ 7.86–7.81 (4H, m), 7.56 (1H, dd, *J* = 8.5, 1.5 Hz), 7.49–7.43 (2H, m), 6.72 (1H, s), 5.96 (1H, ddt, *J* = 17.2, 10.5, 5.7 Hz), 5.88 (1H, dddd, *J* = 12.0, 6.2, 3.2, 2.6 Hz), 5.84–5.73 (3H, m), 5.40 (1H, br d, *J* = 17.2 Hz), 5.31 (1H, br d, *J* = 10.5 Hz), 5.04 (1H, d, *J* = 12.0 Hz), 5.01 (1H, d, *J* = 12.0 Hz), 4.68 (2H, br d, *J* = 5.7 Hz), 4.32 (1H, dd, *J* = 15.6, 5.9 Hz), 4.05 (1H, app. dq, *J* = 15.6, 3.0 Hz), 3.95 (1H, br d, *J* = 7.5 Hz), 3.69 (1H, ddd, *J* = 11.8, 7.5, 4.6 Hz), 3.55 (1H, app. t, *J* = 8.6 Hz), 3.40 (1H,

app. t, J = 8.9 Hz), 3.32 (1H, app. td, J = 9.6, 4.1 Hz), 3.24 (1H, dd, J = 9.2, 8.7 Hz), 3.20 (1H, app. td, J = 10.1, 4.2 Hz), 2.67 (1H, ddd, J = 16.2, 8.0, 4.0 Hz), 2.60 (1H, ddd, J = 11.8, 4.6, 4.4 Hz), 2.40–2.31 (1H, m), 1.70 (1H, ddd, J = 11.8, 11.4, 11.2 Hz). ^{13}C NMR (126 MHz, $CDCl_3$) δ 154.5, 142.1, 136.6, 133.4, 133.3, 133.0, 131.4, 131.1, 129.4, 127.9, 127.6, 126.6, 126.3, 126.0, 125.98, 125.88, 125.6, 121.2, 119.5, 87.4, 81.9, 80.4, 77.3, 76.8, 75.9, 75.2, 72.8, 69.1, 68.4, 36.0, 34.6. $\nu_{max.}$ (film) 3022, 2934, 2872, 1759, 1663, 980, 951, 854, 816, 736 cm^{-1}. HRMS (ESI) calcd. for $C_{31}H_{32}NaO_8$ [M+Na]$^+$ 555.1989, found 555.1965.

A–D Enone 20

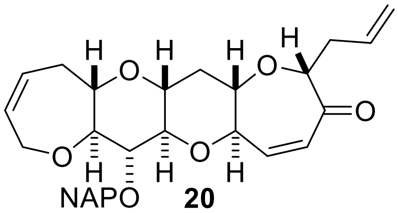

20

To a stirred solution of tetrakis(triphenylphosphine)palladium(0) (23 mg, 20 μmol) in degassed and anhydrous THF (8 mL) at room temperature was added (*R*)-*t*Bu-PHOX (12 mg, 30 μmol) and the mixture was stirred for 30 min. A solution of allyl carbonate **19** (112 mg, 0.210 mmol) in THF (7 mL) was added. The mixture was stirred at room temperature for 2 h and then filtered through Celite. The solvent was evaporated and the residue was purified by flash column chromatography on silica gel (petroleum ether:diethyl ether, 7:3) to afford the enone **20** (77 mg, 75%, *dr* 24:1) as a colourless solid. R_f 0.58 (petroleum ether:diethyl ether, 1:1). Mp 97–99 °C. $[\alpha]_D^{21}$ −84.0 (c = 0.875 in $CHCl_3$). ^{1}H NMR (400 MHz, $CDCl_3$) δ 7.86–7.81 (4H, m), 7.54 (1H, dd, J = 8.4, 1.6 Hz), 7.49–7.45 (2H, m), 6.50 (1H, dd, J = 12.8, 2.4 Hz), 6.00 (1H, dd, J = 12.8, 2.6 Hz), 5.87 (1H, dddd, J = 11.3, 5.8, 3.0, 2.8 Hz), 5.81 (1H, dddd, J = 17.4, 10.5, 7.3, 6.6 Hz), 5.77 (1H, dddd, J = 11.3, 7.8, 3.0, 2.3 Hz), 5.10 (1H, ddd, J = 17.4, 1.7, 1.2 Hz), 5.07 (1H, ddd, J = 10.5, 1.7, 1.2 Hz), 5.02 (2H, s), 4.32 (1H, dd, J = 15.6, 5.8 Hz), 4.23 (1H, dd, J = 7.6, 4.2 Hz), 4.05 (1H, app. dq, J = 15.6, 2.9 Hz), 3.98 (1H, ddd, J = 8.9, 2.6, 2.4 Hz), 3.55 (1H, dd, J = 9.0, 8.6 Hz), 3.44 (1H, ddd, J = 11.3, 8.9, 4.7 Hz), 3.40 (1H, dd, J = 9.0, 8.6 Hz), 3.29 (1H, ddd, J = 10.1, 9.0, 3.9 Hz), 3.26 (1H, dd, J = 9.3, 9.0 Hz), 3.15 (1H, ddd, J = 11.5, 9.3, 4.3 Hz), 2.66 (1H, ddd, J = 16.3, 7.9, 4.0 Hz), 2.62–2.53 (1H, m), 2.49–2.30 (3H, m), 1.68 (1H, ddd, J = 11.8, 11.5, 11.3 Hz). ^{13}C NMR (101 MHz, $CDCl_3$) δ 203.0, 143.5, 136.6, 133.3, 133.2, 133.0, 131.3, 128.1, 127.9, 127.8, 127.6, 126.6, 126.2, 126.0, 125.9, 125.7, 117.7, 87.4, 86.7, 82.0, 81.6, 79.8, 77.0, 76.7, 75.3, 73.1, 68.4, 37.6, 36.2, 34.6. $\nu_{max.}$ (film) 3025, 2931, 2879, 1664, 1641, 1603, 918, 855, 818, 740 cm^{-1}. HRMS (ESI) calcd. for $C_{30}H_{32}NaO_6$ [M+Na]$^+$ 511.2091, found 511.2071.

A–D Allylic alcohol 21[5]

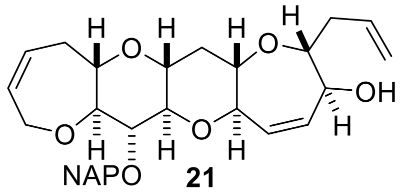

21

To a solution of the enone **20** (422 mg, 0.864 mmol) in dichloromethane (10 ml) and methanol (30 mL), at −78 °C, was added cerium(III) chloride heptahydrate (350 mg, 0.939 mmol) and sodium borohydride (43 mg, 1.1 mmol). The mixture was stirred at −78 °C for 1 h and then the reaction was quenched by the addition of water (20 mL). The mixture was warmed to room temperature and the phases were separated. The aqueous phase was extracted with dichloromethane (3 × 30 mL) and the combined organic extracts were dried (magnesium sulfate) and filtered. The solvent was evaporated under reduced pressure and the residue was purified by flash column chromatography on silica gel (petroleum ether:ethyl acetate, 2:1) to afford the allylic alcohol **21** as a colourless solid (373 mg, 88%). R_f 0.21 (petroleum ether:diethyl ether, 1:1). Mp 171–173 °C (lit.[5] Mp 178–179 °C). $[\alpha]_D^{24}$ −4.9 (*c* = 1.00 in $CHCl_3$) {lit.[4] $[\alpha]_D^{28}$ −5.93 (*c* = 1.01 in $CHCl_3$)}. ^{1}H NMR (500 MHz, $CDCl_3$) δ 7.84–7.80 (4H, m), 7.54 (1H, dd, *J* = 8.5, 1.7 Hz), 7.47–7.41 (2H, m), 5.92 (1H, dddd, *J* = 17.2 10.2, 7.2, 7.0 Hz), 5.87 (1H, dddd, *J* = 12.1, 6.1, 3.1, 2.6 Hz), 5.77 (1H, dddd, *J* = 11.1, 8.0, 3.2, 3.0 Hz), 5.72–5.64 (2H, m), 5.13 (1H, app. dq, *J* = 17.2, 1.8 Hz), 5.07 (1H, app. ddt, *J* = 10.2, 2.2, 1.2 Hz), 5.04 (1H, d, *J* = 12.0 Hz), 4.98 (1H, d, *J* = 12.0 Hz), 4.31 (1H, dd, *J* = 15.6, 5.9 Hz), 4.18–4.12 (1H, m), 4.06–3.99 (1H, m), 3.88–3.83 (1H, m), 3.52 (1H, app. t, *J* = 8.5 Hz), 3.36 (1H, dd, *J* = 9.2, 8.4 Hz), 3.39–3.33 (1H, m), 3.32–3.24 (2H, m), 3.16–3.06 (2H, m), 2.63 (1H, ddd, *J* = 16.3, 8.0, 4.0 Hz), 2.58–2.51 (1H, m), 2.38–2.29 (2H, m), 2.29–2.21 (1H, m), 1.72 (1H, d, *J* = 6.2 Hz), 1.58–1.49 (1H, m). ^{13}C NMR (126 MHz, $CDCl_3$) δ 136.7, 134.9, 134.2, 133.3, 133.0, 131.4, 131.3, 127.90, 127.87, 127.7 126.8, 126.3, 126.1, 125.9, 125.7, 117.1, 87.5, 84.4, 82.1, 81.0, 80.4, 78.7, 76.8, 75.2, 73.7, 73.3, 68.4, 37.6, 36.7, 34.6. $\nu_{max.}$ (film) 3479, 3327, 3023, 2931, 2882, 1642, 1603, 914, 852, 818 cm^{-1}. HRMS (ESI) calcd. for $C_{30}H_{34}NaO_6$ $[M+Na]^+$ 513.2248, found 513.2225.

A–D Enone 22

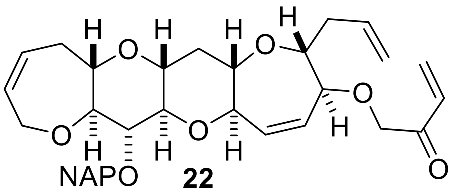

22

To a suspension of sodium hydride (120 mg, from 200 mg of a 60% suspension in mineral oil, 3.0 mmol) in anhydrous THF (30 mL) was added dropwise, at 0 °C, solution of the alcohol **21** (373 mg, 0.760 mmol) in anhydrous THF (15 mL) and the mixture was stirred at room temperature for 20 min. 1-Chloro-3-(triphenylphosphoranylidene)-2-propanone[4] (535 mg, 1.52 mmol) was added followed by tetra-*n*-butylammonium iodide (70 mg, 0.19 mmol) and the resulting mixture was heated at reflux for

12 h. The reaction was quenched by the cautious addition of water (20 mL) and the mixture was diluted with ethyl acetate (20 mL). The phases were separated, and the aqueous phase was extracted with ethyl acetate (2 × 20 mL). The combined organic extracts were washed with brine (20 mL), dried (magnesium sulfate) and filtered. The solvent was removed under reduced pressure to afford the crude product.

To a solution of the crude phosphonium ylide in THF (15 mL) was added pH 7 phosphate buffer solution (15 mL) followed by solution of formaldehyde (1.23 mL of 37% w/w solution in water, 15.2 mmol). The resulting mixture was stirred at room temperature for 2 h and then diluted with dichloromethane (40 mL). The phases were separated, and the aqueous phase was extracted with dichloromethane (40 mL). The combined organic extracts were washed with water (30 mL), dried (magnesium sulfate) and filtered. The solvent was evaporated under reduced pressure and the residue was purified by flash column chromatography on silica gel (petroleum ether:ethyl acetate, 3:1) to afford the enone **22** (276 mg, 65% over 2 steps) as colourless solid. R_f 0.45 (petroleum ether:diethyl ether, 1:1). Mp 146–147 °C. $[\alpha]_D^{26}$ +25.7 (*c* = 1.10 in $CHCl_3$). ^1H NMR (400 MHz, $CDCl_3$) δ 7.85–7.80 (4H, m), 7.55 (1H, dd, *J* = 8.5, 1.7 Hz), 7.48–7.43 (2H, m), 6.54 (1H, dd, *J* = 17.6, 10.7 Hz), 6.35 (1H, dd, *J* = 17.6, 1.4 Hz), 5.95–5.82 (2H, m), 5.85 (1H, dd, *J* = 10.7, 1.4 Hz), 5.81–5.73 (3H, m), 5.11–5.03 (2H, m), 5.04 (1H, d, *J* = 12.1 Hz), 4.99 (1H, d, *J* = 12.1 Hz), 4.39 (1H, d, *J* = 16.7 Hz), 4.31 (1H, dd, *J* = 15.5, 6.0 Hz), 4.28 (1H, d, *J* = 16.7 Hz), 4.04 (1H, app. dq, *J* = 15.5, 2.9 Hz), 3.91–3.84 (2H, m), 3.58–3.48 (2H, m), 3.38 (1H, dd, *J* = 9.0, 8.6 Hz), 3.34–3.23 (2H, m), 3.18–3.07 (2H, m), 2.46 (1H, ddd, *J* = 16.3, 7.9, 3.9 Hz), 2.63–2.55 (1H, m), 2.40–2.20 (3H, m), 1.55 (1H, app. q, *J* = 11.3 Hz). ^{13}C NMR (101 MHz, $CDCl_3$) δ 196.0, 136.7, 134.8, 133.3, 133.0, 132.7, 132.3, 131.3, 131.1, 129.3, 127.9, 127.8, 127.6, 126.7, 126.2, 126.0, 125.9, 125.6, 117.0, 87.5, 83.1, 82.1, 81.7, 81.0, 80.4, 78.4, 76.8, 75.1, 73.7, 73.2, 68.4, 37.2, 36.6, 34.6. $\nu_{max.}$ (film) 3022, 2936, 2895, 2830, 1711, 1641, 1619, 984, 909, 855, 816 cm^{-1}. HRMS (ESI) calcd. for $C_{34}H_{38}NaO_7$ $[M+Na]^+$ 581.2510, found 581.2491.

A–E Enone 23

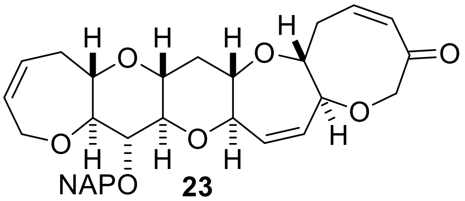

23

To a stirred solution of tetraene **22** (44 mg, 79 μmol) in anhydrous dichloromethane (15 mL) at room temperature was added the Grubbs II catalyst (8.0 mg, 8.0 μmol). The mixture was sparged with argon for 10 min, then heated to reflux and stirred overnight at reflux. The solvent was evaporated under reduced pressure and the residue was purified by flash column chromatography on silica gel (hexane:ethyl acetate, 4:1) to afford the pentacyclic enone **23** (26 mg, 63%) as a beige solid. R_f 0.35 (petroleum ether:diethyl ether, 1:1). Mp 105–106 °C. $[\alpha]_D^{25}$ +2.6 (*c* = 0.74 in $CHCl_3$). ^1H NMR (400

MHz, $CDCl_3$) δ 7.86–7.79 (4H, m), 7.54 (1H, dd, *J* = 8.4, 1.6 Hz), 7.49–7.43 (2H, m), 6.44 (1H, ddd, *J* = 12.4, 7.5, 7.4 Hz), 5.92–5.73 (5H, m), 5.04 (1H, d, *J* = 12.0 Hz), 5.00 (1H, d, *J* = 12.0 Hz), 4.45 (1H, d, *J* = 18.0 Hz), 4.32 (1H, dd, *J* = 15.6, 5.8 Hz), 4.27–4.21 (1H, m), 4.21 (1H, d, *J* = 18.0 Hz), 4.05 (1H, app. dq, *J* = 15.6, 2.9 Hz), 3.88–3.82 (1H, m), 3.58 (1H, ddd, *J* = 9.5, 9.0, 1.8 Hz), 3.53 (1H, dd, *J* = 8.6, 8.6 Hz), 3.38 (1H, dd, *J* = 8.9, 8.6 Hz), 3.34–3.24 (2H, m), 3.17–3.07 (2H, m), 2.84–2.72 (1H, m), 2.68–2.57 (2H, m), 2.39–2.26 (2H, m), 1.58–1.48 (1H, m). ^{13}C NMR (101 MHz, $CDCl_3$) δ 203.5, 139.3, 136.6, 133.2, 132.9, 132.7, 132.4, 131.3, 128.6, 127.8 (2C), 127.6, 126.6, 126.3, 126.0, 125.9, 125.6, 87.4, 85.8, 81.9, 80.9, 80.2, 79.9, 78.5, 76.8, 76.7, 75.1, 73.0, 68.3, 37.3, 36.8, 34.6. $\nu_{max.}$ (film) 3055, 3028, 2922, 2893, 1682, 999, 957, 854, 816 cm^{-1}. HRMS (ESI) calcd. for $C_{32}H_{34}NaO_7$ $[M+Na]^+$ 553.2197, found 553.2186.

A–E Enol carbonate 24

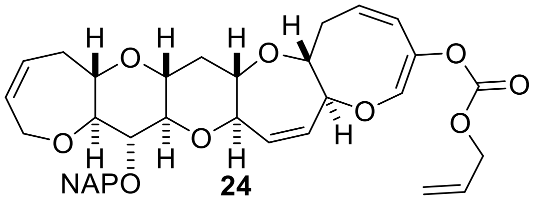

To a solution of enone **23** (114 mg, 0.215 mmol) in anhydrous THF (5 mL) was added allyl chloroformate (117 μL, 1.10 mmol) and the solution was cooled to −78 °C. Sodium bis(trimethylsilyl)amide (220 μL of a 2.0 M solution in THF, 0.44 mmol) was added dropwise and the solution was stirred at −78 °C for 1 h. The reaction was quenched by addition of an aqueous solution of potassium dihydrogenphosphate (5 mL) and the resulting mixture was warmed to room temperature and diluted with dichloromethane (15 mL). The phases were separated, and the aqueous phase was extracted with dichloromethane (10 mL). The combined organic extracts were dried (magnesium sulfate) and filtered. The solvent was evaporated under reduced pressure and the residue was purified by flash column chromatography on silica gel (petroleum ether:ethyl acetate, 8:1) to afford the enol carbonate **24** (116 mg, 88%) as a colourless foam that was used immediately in the subsequent reaction. R_f 0.45 (petroleum ether:diethyl ether, 1:4). Mp 138–140 °C. $[\alpha]_D^{25}$ −82 (*c* = 0.51 in $CHCl_3$). ^1H NMR (500 MHz, $CDCl_3$) δ 7.66–7.61 (4H, m), 7.36 (1H, dd, *J* = 8.5, 1.6 Hz), 7.28–7.25 (2H, m), 6.41 (1H, s), 5.79–5.54 (6H, m), 5.52 (1H, dd, *J* = 13.0, 2.0 Hz), 5.49 (1H, dd, *J* = 13.0, 2.0 Hz), 5.18 (1H, dd, *J* = 17.2, 1.6 Hz), 5.10 (1H, d, *J* = 10.3 Hz), 4.85 (1H, d, *J* = 12.1 Hz), 4.80 (1H, d, *J* = 12.1 Hz), 4.45 (2H, d, *J* = 5.8 Hz), 4.12 (1H, dd, *J* = 15.6, 5.9 Hz), 3.84 (1H, app. dq, *J* = 15.6, 2.9 Hz), 3.64 (1H, app. d, *J* = 9.0 Hz), 3.37 (1H, app. dt, *J* = 9.0, 3.3 Hz), 3.33 (1H, app. t, *J* = 8.5 Hz), 3.18 (1H, app. t, *J* = 8.9 Hz), 3.15–3.05 (2H, m), 2.96–2.87 (2H, m), 2.54 (1H, ddd, *J* = 13.6, 9.0, 2.7 Hz), 2.45 (1H, ddd, *J* = 16.3, 8.0, 4.0 Hz), 2.33 (1H, ddd, *J* = 13.6, 7.1, 3.6 Hz), 2.20–2.06 (2H, m), 1.54 (1H, ddd, *J* = 11.5, 11.2, 10.5 Hz). ^{13}C NMR (126 MHz, $CDCl_3$) δ 154.5, 140.9, 136.6, 133.2, 132.9, 132.4, 131.3, 131.13, 131.12, 128.3, 127.79, 127.77, 127.6, 126.6, 126.2, 126.0, 125.8, 125.6, 125.2, 119.2, 87.4, 81.9, 80.8, 80.1, 78.3, 77.9, 76.7, 76.0, 75.1, 73.0, 68.9,

68.3, 36.8, 34.5, 33.0. $\nu_{max.}$ (film) 3034, 2920, 2893, 2874, 2851, 1759, 1651, 1632, 1603, 972, 951, 818 cm^{-1}. HRMS (ESI) calcd. for $C_{36}H_{38}NaO_9$ $[M+Na]^+$ 637.2408, found 637.2393.

A–E Enone 25

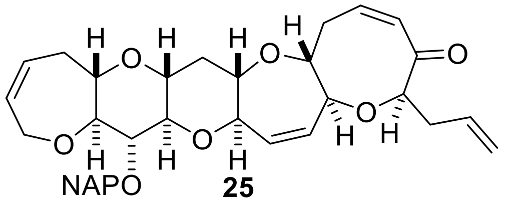

25

To a solution of (*R*)-*t*-BuPHOX ligand (11 mg, 0.028 mmol) in anhydrous and degassed THF (6 mL) at room temperature was added tetrakis(triphenylphosphine)palladium(0) (22 mg, 0.019 mmol) and the mixture was stirred for 30 min. A solution of the carbonate **24** (116 mg, 0.189 mmol) in anhydrous and degassed THF (4 mL) was added and the solution was stirred at room temperature for 2 h. The solvent was evaporated under reduced pressure and the residue was purified by flash column chromatography on silica gel (pentane:ethyl acetate, 4:1 to 3:1) to afford the desired pentacyclic enone **25** (84 mg, 78 %) as a mixture (3:1) of diastereomers as a colourless solid.

To a solution of the enones synthesized above (84mg, 0.15 mmol) in benzene (7.5 mL) was added Barton's base (61 µL, 0.30 mmol) and the mixture was heated at 50 °C for 16 h. The solution was cooled to room temperature and the solvent was evaporated under reduced pressure. The residue was purified by flash column chromatography on silica gel (pentane:ethyl acetate, 4:1 to 3:1) to afford the desired enone **25** (68 mg, 81%) as colourless solid (diastereomeric ratio 10:1). R_f 0.42 (petroleum ether:diethyl ether, 1:1). Mp 182–183 °C. $[\alpha]_D^{23}$ +41 (*c* = 0.50 in $CHCl_3$). ^1H NMR (400 MHz, $CDCl_3$) δ 7.85–7.81 (4H, m), 7.56 (1H, dd, *J* = 8.5, 1.5 Hz), 7.48–7.45 (2H, m), 6.42 (1H, ddd, *J* = 12.1, 9.0, 8.9 Hz), 5.89 (1H, app. d, *J* = 12.1 Hz), 5.92–5.68 (6H, m), 5.21–5.11 (2H, m), 5.04 (1H, d, *J* = 12.0 Hz), 4.99 (1H, d, *J* = 12.0 Hz), 4.32 (1H, dd, *J* = 15.5, 5.8 Hz), 4.26 (1H, dd, *J* = 8.7, 3.5 Hz), 4.19 (1H, app. dq, *J* = 8.8, 2.3 Hz), 4.04 (1H, app. dq, *J* = 15.5, 2.9 Hz), 3.87 (1H, app. dq, *J* = 9.0, 2.3 Hz), 3.56–3.45 (2H, m), 3.38 (1H, dd, *J* = 9.0, 8.5 Hz), 3.41–3.25 (2H, m), 3.17–3.06 (2H, m), (1H, app. dt, *J* = 13.7, 9.2 Hz), 2.77–2.60 (2H, m), 2.56 (1H, ddd, *J* = 13.7, 9.0, 1.8 Hz), 2.40–2.27 (3H, m), 1.58–1.46 (1H, m). ^{13}C NMR (101 MHz, $CDCl_3$) δ 202.6, 137.3, 136.6, 133.9, 133.8, 133.2, 132.9, 131.4, 131.3, 130.6, 127.8 (2C), 127.6, 126.6, 126.2, 126.0, 125.8, 125.6, 118.4, 88.1, 87.5, 87.3, 81.9, 80.9, 80.2, 79.6, 78.0, 76.7, 75.1, 73.0, 68.3, 37.2, 36.8, 35.6, 34.5. $\nu_{max.}$ (film) 2922, 2882, 2855, 1738, 1688, 1643, 816 cm^{-1}. HRMS (ESI) calcd. for $C_{35}H_{38}NaO_7$ $[M+Na]^+$ 593.2510, found 593.2485.

A–E Allylic alcohol 26[3]

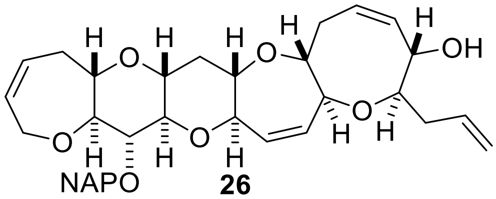

26

To a solution of the enone **25** (68 mg, 0.12 mmol) in dichloromethane (4 ml) and methanol (4 mL), at −78 °C, was added cerium(III) chloride heptahydrate (147 mg, 0.395 mmol) and sodium borohydride (27 mg, 0.71 mmol). The mixture was stirred at −78 °C for 1 h and then water (10 mL) was added. The mixture was warmed to room temperature and the phases were separated. The aqueous phase was washed with dichloromethane (3 × 10 mL) and the combined extracts were dried (magnesium sulfate) and filtered. The solvent was evaporated under reduced pressure and the residue was purified by flash column chromatography on silica gel (pentane:ethyl acetate, 4:1 to 2:1) to afford the desired allylic alcohol **26** (53.8 mg, 79%) as a colourless solid. R_f 0.15 (petroleum ether:diethyl ether, 1:1). Mp 141–142 °C. $[\alpha]_D^{24}$ −56 (*c* = 0.29 in $CHCl_3$) {lit.[3] $[\alpha]_D^{21}$ −62.3 (*c* = 1.00 in $CHCl_3$)}. ^1H NMR (400 MHz, $CDCl_3$) δ 7.84–7.77 (4H, m), 7.55 (1H, dd, *J* = 8.5, 1.6 Hz), 7.48–7.42 (2H, m), 5.95 (1H, dddd, *J* = 17.2, 10.2, 7.5, 6.6 Hz), 5.90–5.67 (5H, m), 5.62 (1H, app. dt, *J* = 12.7, 2.6 Hz), 5.19–5.10 (2H, m), 5.03 (1H, d, *J* = 12.0 Hz), 4.98 (1H, d, *J* = 12.0 Hz), 4.31 (1H, dd, *J* = 15.6, 5.9 Hz), 4.26–4.20 (1H, m), 4.12–3.99 (1H, m), 4.04 (1H, app. dq, *J* = 15.6, 2.9 Hz), 3.81 (1H, app. dq, *J* = 9.0, 2.4 Hz), 3.60 (1H, ddd, *J* = 8.8, 3.4, 3.2 Hz), 3.55–3.47 (1H, m), 3.37 (1H, dd, *J* = 9.0, 8.7 Hz), 3.40–3.18 (3H, m), 3.15–3.05 (2H, m), 2.71 (1H, ddd, *J* = 13.2, 9.5, 3.8 Hz), 2.68–2.60 (2H, m), 2.40–2.17 (4H, m), 1.59–1.46 (2H, m). ^{13}C NMR (101 MHz, $CDCl_3$) δ 136.7, 136.5, 135.4, 135.3, 133.3, 133.0, 131.4, 130.5, 127.88, 127.85, 127.7, 126.8, 126.8, 126.3, 126.1, 125.9, 125.6, 117.1, 87.4, 85.7, 84.6, 82.0, 81.1, 80.9, 80.6, 78.0, 76.8, 75.1, 73.2, 72.1, 68.4, 37.4, 36.9, 34.6, 32.5. $\nu_{max.}$ (film) 2880, 1647, 914, 856, 818 cm^{-1}. HRMS (ESI) calcd. for $C_{35}H_{40}NaO_7$ $[M+Na]^+$ 595.2666, found 595.2638.

A–E Carboxylic acid S3

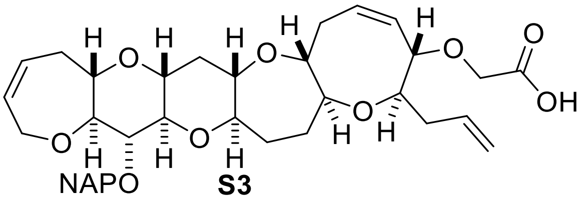

S3

To a solution of alcohol **26** (51 mg, 0.089 mmol) in benzene (1 mL) was added *t*-butyl bromoacetate (132 μL, 0.894 mmol) followed by tetra-*n*-butylammonium hydrogensulfate (6.1 mg, 0.018 mmol). A 50% aqueous solution of sodium hydroxide (1 mL) was added and the biphasic mixture was stirred vigorously at room temperature until consumption of the starting material as judged by TLC (4 h). Methanol (10 mL) was added and the mixture was stirred at room temperature for 12 h. The volatiles were removed under reduced pressure and water (3 mL) and diethyl ether (5 mL) were added. A 3

M solution of hydrochloric acid was added dropwise while stirring vigorously, until the aqueous phase was strongly acidic. The phases were separated and the aqueous phase was washed with diethyl ether (3 × 5 mL). The combined organic extracts were washed with water (2 × 3 mL) and brine (5 mL), then dried (magnesium sulfate) and filtered. The solvent was evaporated under reduced pressure and the residue was purified by flash column chromatography on silica gel (pentane:ethyl acetate, 3:1 to 0:1) to afford the carboxylic acid **S3** (50 mg, 89%) as a colourless solid. R_f 0.10 (ethyl acetate). Mp 119–121 °C. $[\alpha]_D^{18}$ −25 (*c* = 0.50 in $CHCl_3$). ^1H NMR (500 MHz, $CDCl_3$) δ 7.84–7.79 (4H, m), 7.54 (1H, dd, *J* = 8.4, 1.7 Hz), 7.48–7.45 (2H, m), 5.97–5.74 (5H, m), 5.71 (1H, dd, *J* = 11.2, 5.4 Hz), 5.62 (1H, app. dt, *J* = 12.7, 2.5 Hz), 5.17–5.10 (2H, m), 5.03 (1H, d, *J* = 11.9 Hz), 4.98 (1H, d, *J* = 11.9 Hz), 4.32 (1H, dd, *J* = 15.6, 5.9 Hz), 4.21 (1H, d, *J* = 16.8 Hz), 4.05 (1H, d, *J* = 16.8 Hz), 4.09–3.96 (3H, m), 3.81 (1H, app. dq, *J* = 9.0, 2.3 Hz), 3.61 (1H, ddd, *J* = 9.0, 3.5, 3.0 Hz), 3.56–3.48 (1H, m), 3.44 (1H, app. td, *J* = 9.1, 2.8 Hz), 3.37 (1H, dd, *J* = 9.0, 8.7 Hz), 3.28 (1H, ddd, *J* = 9.7, 9.3, 4.0 Hz), 3.23 (1H, ddd, *J* = 11.2, 8.9, 4.6 Hz), 3.14–3.07 (2H, m), 2.76–2.67 (2H, m), 2.64 (1H, ddd, *J* = 16.2, 7.9, 3.9 Hz), 2.39–2.19 (4H, m), 1.58–1.47 (1H, m). ^{13}C NMR (126 MHz, $CDCl_3$) δ 174.2, 136.6, 135.4, 135.0, 133.7, 133.3, 133.0, 131.3, 130.5, 130.0, 127.9, 127.8, 127.6, 126.8, 126.3, 126.1, 125.8, 125.6, 117.2, 87.4, 84.8, 84.5, 81.9, 81.2, 80.8, 80.5, 79.9, 77.9, 77.2, 75.1, 73.1, 68.3, 65.8, 37.3, 36.8, 34.6, 32.5. $\nu_{max.}$ (film) 2924, 2855, 1730, 1643, 914, 854, 816, 752, 683, 667, 646, 598, 582 cm^{-1}. HRMS (ESI) calcd. for $C_{37}H_{42}NaO_9$ $[M+Na]^+$ 653.2721, found 653.2698.

A–E Imide 27

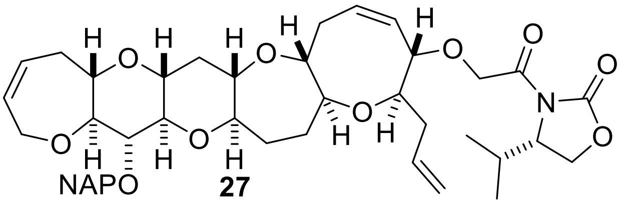

27

To a solution of the carboxylic acid **S3** (25 mg, 0.040 mmol) in anhydrous THF (2 mL), at −78 °C, was added triethylamine (34 μL, 0.24 mmol) followed by pivaloyl chloride (59 μL, 0.48 mmol). The solution was warmed to 0 °C and stirred for 1 h (monitored by TLC). To a separate flask containing solution of (*S*)-4-isopropyl-2-oxazolidinone (82 mg, 0.63 mmol) in anhydrous THF (6 mL) was added *n*-butyllithium (225 μL of a 2.5 M solution in hexane, 0.56 mmol) dropwise at −78 °C and the solution was stirred for a further 30 min at −78 °C. The flask containing the mixed anhydride was cooled to −78 °C and a portion (3 mL) of the solution of the lithiated oxazolidinone was added by syringe. The mixture was stirred 15 min at −78 °C and then warmed to rt and stirred for a further 1 h. The reaction was quenched by addition of a saturated aqueous solution of ammonium chloride (4 mL) and the mixture was diluted with ethyl acetate (5 mL). The phases were separated and the aqueous was extracted with ethyl acetate (2 × 5 mL). The combined organic extracts were washed with brine (5 mL), dried (magnesium sulfate) and filtered. The solvent was evaporated under reduced pressure and the residue was purified by flash column chromatography on silica gel (pentane:ethyl acetate,

5:1 to 2:1) to afford the imide **27** (26 mg, 88%) as a colourless solid. R_f 0.35 (petroleum ether:ethyl acetate, 1:1). Mp 179–180 °C. $[\alpha]_D^{18}$ −1.7 (*c* = 0.60 in $CHCl_3$). ^{1}H NMR (500 MHz, $CDCl_3$) δ 7.84–7.70 (4H, m), 7.54 (1H, dd, *J* = 8.4, 1.6 Hz), 7.47–7.44 (2H, m), 5.98–5.72 (5H, m), 5.61 (1H, app. dt, *J* = 12.7, 2.5, 2.5 Hz), 5.14 (1H, dd, *J* = 17.5, 2.0 Hz) 5.11 (1H, dd, *J* = 10.5, 2.0 Hz), 5.03 (1H, d, *J* = 11.9 Hz), 4.97 (1H, d, *J* = 11.9 Hz), 4.76 (1H, d, *J* = 17.9 Hz), 4.61 (1H, d, *J* = 17.9 Hz), 4.43 (1H, app. dt, *J* = 8.5, 3.4 Hz), 4.33 (1H, dd, *J* = 8.9, 8.7 Hz), 4.31 (1H, dd, *J* = 15.6, 5.9 Hz), 4.26 (1H, dd, *J* = 9.2, 3.1 Hz), 4.09–3.97 (3H, m), 3.81 (1H, app. dq, *J* = 9.0, 2.4 Hz), 3.60 (1H, app. dt, *J* = 8.7, 3.3 Hz), 3.54–3.48 (1H, m), 3.46 (1H, app. td, *J* = 9.2, 2.5 Hz), 3.36 (1H, dd, *J* = 9.0, 8.6 Hz), 3.27 (1H, app. td, *J* = 9.8, 4.0 Hz), 3.22 (1H, ddd, *J* = 11.2, 8.9, 4.4 Hz), 3.14–3.06 (2H, m), 2.80 (1H, ddd, *J* = 14.5, 6.6, 2.2 Hz), 2.73–2.66 (1H, m), 2.70 (1H, ddd, *J* = 13.7, 10.5, 3.8 Hz), 2.64 (1H, ddd, *J* = 16.2, 7.9, 3.9 Hz), 2.43 (1H, qqd, J = 7.0, 6.9, 3.9 Hz), 2.38–2.21 (4H, m), 1.56–1.46 (1H, m), 0.92 (3H, d, *J* = 7.0 Hz), 0.88 (3H, d, *J* = 6.9 Hz). ^{13}C NMR (126 MHz, $CDCl_3$) δ 169.7, 154.0, 136.6, 135.7, 135.2, 134.3, 133.2, 132.9, 131.3, 130.4, 129.5, 127.9, 127.8, 127.6, 126.8, 126.3, 126.1, 125.8, 125.6, 116.9, 87.4, 85.1, 84.6, 82.0, 81.1, 80.9, 80.5, 79.9, 78.0, 75.1, 73.2, 68.6, 68.3, 64.3, 58.2, 37.3, 36.8, 34.6, 32.5, 29.7, 28.1, 17.9, 14.6. $\nu_{max.}$ (film) 2959, 2924, 2872, 2855, 1780, 1717, 968, 854, 818, 754, 718, 683 cm^{-1}. HRMS (ESI) calcd. for $C_{43}H_{51}NNaO_{10}$ $[M+Na]^+$ 764.3405, found 764.3404.

A–E Nitrile 28

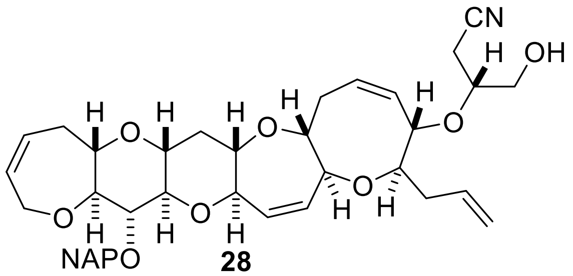

28

To a mixture of sodium bis(trimethylsilyl)amide (112 µL of a 0.6 M solution in toluene, 0.10 mmol) and TMEDA (30 µL, 0.20 mmol) in anhydrous toluene (200 µL) at −78 °C was added dropwise a solution of imide **27** (25 mg, 34 µmol) in anhydrous toluene (400 µL) and the solution was stirred at −78 °C for 30 min. Bromoacetonitrile (42 µL, 0.60 mmol) was added dropwise (colour of the solution became dark red/brown) and the mixture was stirred at −78 °C for 4 h. The reaction was quenched by the addition of a saturated aqueous solution of ammonium chloride (1 mL) and the mixture was warmed to room temperature. The mixture was diluted with ethyl acetate (1 mL) and the phases were separated. The aqueous phase was extracted with ethyl acetate (2 × 1 mL) and the combined organic extracts were then washed with brine (1 mL), then dried (magnesium sulfate) and filtered. The solvent was evaporated under reduced pressure and the residue was purified with flash column chromatography on silica gel (pentane:ethyl acetate 4:1 to 3:1) to afford the expected alkylated product (8.0 mg, 30%) as a white solid along with unreacted imide **28** (7 mg, 28%).

To a solution of the alkylation product (8.0 mg, 10 μmol) in THF (400 μL) and water (100 μL) was added sodium borohydride (1.9 mg, 50 μmol) and the resulting solution was stirred at room temperature for 2 h. The reaction was quenched by the addition of Rochelle salt (1 mL) and the mixture was diluted with ethyl acetate (1 mL) and stirred vigorously for 30 min. The phases were separated, and the aqueous phase was extracted with ethyl acetate (3 × 1 mL). The combined organic extracts were washed with brine (1 mL), then dried (magnesium sulfate) and filtered. The solvent was evaporated under reduced pressure and the residue was purified with flash column chromatography on silica gel (pentane:diethyl ether, 1:1 to 1:2) to deliver the alcohol **28** (6.0 mg, 89%) as a colourless solid. R_f 0.20 (petroleum ether:ethyl acetate, 1:1). $[\alpha]_D^{22}$ −28.0 (*c* = 0.225 in $CHCl_3$). ^{1}H NMR (500 MHz, $CDCl_3$) δ 7.85–7.80 (4H, m), 7.54 (1H, dd, *J* = 8.4, 1.6 Hz), 7.47–7.43 (2H, m), 5.98–5.84 (3H, m), 5.82–5.73 (3H, m), 5.61 (1H, app. dt, *J* = 12.7, 2.5 Hz), 5.18–5.12 (2H, m), 5.03 (1H, d, *J* = 12.0 Hz), 4.98 (1H, d, *J* = 12.0 Hz), 4.31 (1H, dd, *J* = 15.6, 5.9 Hz), 4.09–4.00 (3H, m), 3.83–3.73 (3H, m), 3.68–3.58 (2H, m), 3.54–3.48 (1H, m), 3.43 (1H, app. td, *J* = 8.7, 3.0 Hz), 3.37 (1H, dd, *J* = 8.9, 8.3 Hz), 3.27 (1H, ddd, *J* = 9.8, 9.4, 4.0 Hz), 3.22 (1H, ddd, *J* = 11.2, 8.9, 4.6 Hz), 3.14–3.06 (2H, m), 2.72–2.59 (5H, m), 2.39–2.24 (3H, m), 2.20 (1H, app. dt, *J* = 14.5, 8.2 Hz), 1.68 (1H, dd, *J* = 7.4, 4.2 Hz), 1.55–1.47 (1H, m). ^{13}C NMR (126 MHz, $CDCl_3$) δ 136.6, 135.3, 135.0, 134.3, 133.3, 133.0, 131.4, 130.7, 129.6, 127.88, 127.85, 127.7, 126.8, 126.3, 126.1, 125.9, 125.6, 117.4, 117.3, 87.4, 84.5, 84.4, 82.0, 81.3, 80.9, 80.5, 77.99, 77.96, 75.1, 73.5, 73.2, 68.4, 62.0, 37.4, 36.8, 34.6, 32.5, 21.0. $\nu_{max.}$ (film) 3500, 2926, 2873, 2250, 1643, 915, 856, 818 774, 737, 684 cm^{-1}. HRMS (ESI) calcd. for $C_{39}H_{45}NNaO_8$ $[M+Na]^+$ 678.3037, found 678.3030.

A–F Alcohol S4

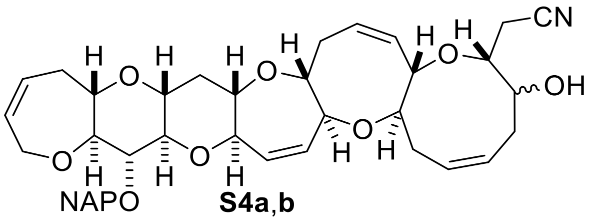

S4a,**b**

To a solution of the cyano alcohol **27** (6.0 mg, 9.1 μmol) in anhydrous dichloromethane (400 μL) was added sodium bicarbonate (6.0 mg, 71 μmol) followed by Dess-Martin Periodinane (7.8 mg, 18w μmol). The reaction mixture was stirred at room temperature for 1 h, diluted with dichloromethane (1 mL) and then quenched with a saturated aqueous solution of sodium thiosulfate (1 mL). The biphasic mixture was stirred vigorously for 30 min (until both layers were clear). The phases were separated, and the aqueous phase was extracted with dichloromethane (2 × 1 mL) and the combined organic extracts were washed with a saturated aqueous solution of sodium bicarbonate, then dried (magnesium sulfate) and filtered. The solvent was evaporated under reduced pressure and the residue was filtered through a thin pad of silica gel using diethyl ether to afford the required aldehyde (4.7 mg, 78%) as a colourless solid that was used in the next reaction without further purification.

To a solution of the aldehyde (4.7 mg, 7.2 μmol) in anhydrous dichloromethane (500 μL) was added allyltributylstannane (10 μL, 32 μmol). The solution was cooled to −78 °C and solution of trimethylaluminium (43 μL of a 2.0 M solution in toluene, 86 μmol) in anhydrous dichloromethane (150 μL) was added dropwise over approximately 1 h. After the addition was completed, the mixture was stirred at −78 °C for an additional 30 min. The reaction mixture was warmed to 0 °C, methanol (100 μL) was added dropwise and the solution was stirred for a further 30 min at 0 °C. The mixture was diluted with dichloromethane (1 mL) and a saturated aqueous solution of Rochelle salt (1 mL) was added. The biphasic mixture was stirred vigorously for 30 min and the phases were then separated. The aqueous phase was extracted with dichloromethane (3 × 1 mL) and the combined organic extracts were washed with water (1 mL), then dried (magnesium sulfate) and filtered. The solvent was evaporated under reduced pressure and the residue was purified by flash column chromatography on silica gel (pentane:diethyl ether, 5:1 to 2:1 to 1:1) to afford a diastereomeric mixture (3:1) of the alcohol **29a**,**b** (3.0 mg, 60 %) as a colourless oil that was used immediately in the subsequent reaction.

To a solution of the homoallylic alcohols synthesized above (3.0 mg, 4.3 μmol) in anhydrous and degassed dichloromethane (5 mL) was added Hoveyda-Grubbs II catalyst (0.70 mg, 1.1 μmol) and the solution was stirred at room temperature until complete consumption of starting material (16 h). Methanol (200 μL) was added, and the solvent evaporated under reduced pressure. The residue was purified by flash column chromatography on silica gel (pentane:diethyl ether, 2:1 to 1:3) to afford a diastereomeric mixture of hexacycle alcohol **S4a**,**b** (1.5 mg, 56%) as a colourless solid. R_f 0.45 (petroleum ether:ethyl acetate, 1:1). ^{1}H NMR (500 MHz, $CDCl_3$) δ 7.84–7.80 (4H, m), 7.54 (1H, dd, *J* = 8.5, 1.6 Hz), 7.48–7.44 (2H, m), 5.98–5.84 (3H, m), 5.82–5.74 (3H, m), 5.61 (1H, app. dt, *J* = 12.7, 2.5 Hz), 5.18–5.12 (2H, m), 5.03 (1H, d, *J* = 12.0 Hz), 4.97 (1H, d, *J* = 12.0 Hz), 4.31 (1H, dd, *J* = 15.6, 5.9 Hz), 4.09–4.00 (3H, m), 3.83–3.73 (3H, m), 3.67–3.58 (2H, m), 3.54–3.49 (1H, m), 3.44 (1H, app. td, *J* = 8.7, 3.0 Hz), 3.37 (1H, app. t, *J* = 8.6 Hz), 3.27 (1H, ddd, *J* = 9.8, 9.4, 4.0 Hz), 3.22 (1H, ddd, *J* = 11.2, 9.0, 4.6 Hz), 3.14–3.06 (2H, m), 2.72–2.55 (5H, m), 2.38–2.24 (3H, m), 2.20 (1H, app. dt, *J* = 14.5, 8.2 Hz), 1.70–1.65 (1H, m) 1.55–1.47 (1H, m). ^{13}C NMR (151 MHz, C_6D_6) δ 137.8, 136.3, 134.0, 133.5, 131.7, 131.3, 129.0, 128.4, 128.1, 127.9, 127.6, 126.8, 126.6, 126.5, 126.4, 126.2, 125.8, 117.2, 87.9, 86.2 (br) 85.2, 82.7, 82.5, 81.6, 80.9, 78.4, 77.3, 75.2, 73.4, 72.1 (br), 71.0 (br) 68.5, 37.5, 35.1, 33.0 (br), 32.7, 21.6. HRMS (ESI) calcd. for $C_{40}H_{45}NNaO_8$ $[M+Na]^+$ 690.3037, found 690.3021.

A–F Ketone 30

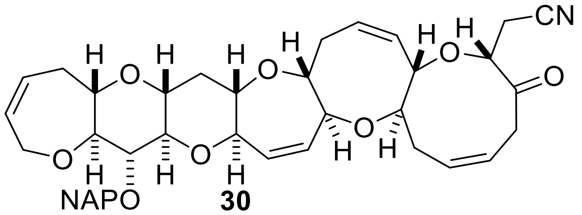

30

To a stirred solution of alcohol **S4a**,**b** (2 mg, 3.0 µmol) in CH_2Cl_2 (1 mL) was added *N*-methylmorpholine *N*-oxide (0.7 mg, 6.0 µmol) and activated 4 Å molecular sieves. The reaction was stirred at room temperature for 30 min and then tetra-*n*-propylammonium perruthenate (22 µL of a 0.014 M solution in dichloromethane, 0.3 µmol) was added. The reaction was stirred for 5 h and then filtered through a plug of silica and washed with excess ethyl acetate. The solvent was removed under reduced pressure and the residue was purified by flash column chromatography on silica gel (hexane:ethyl acetate, 3:1) to afford the hexacyclic ketone **30** (1.3 mg, 65 %) as a white solid. R_f 0.47 (petroleum ether:ethyl acetate, 2:1). $[\alpha]_D^{19}$ +13 (*c* = 0.10 in $CHCl_3$). ^1H NMR (400 MHz, C_6D_6) δ 7.96 (1H, s), 7.77–7.65 (4H, m), 7.32–7.22 (2H, m), 5.87–5.74 (4H, m), 5.70 (1H, dd, *J* = 11.1, 5.2 Hz), 5.58–5.44 (2H, m), 5.29–5.19 (1H, m), 5.25 (1H, d, *J* = 12.4 Hz), 5.18 (1H, d, *J* = 12.4 Hz), 4.13 (1H, dd, *J* = 15.6, 5.5 Hz), 3.97 (1H, app. dq, *J* = 15.6, 2.6 Hz), 3.83–3.76 (2H, m), 3.72−3.60 (3H, m), 3.46 (1H, ddd, *J* = 8.7, 3.3, 3.2 Hz), 3.40 (1H, dd, *J* = 8.9, 8.6 Hz), 3.29 (1H, app. td, *J* = 9.4, 4.0 Hz), 3.27–3.19 (3H, m), 3.07 (1H, ddd, *J* = 11.2, 9.0, 4.7 Hz), 2.94 (1H, ddd, *J* = 11.7, 9.3, 4.4 Hz), 2.72 (1H, app. td, *J* = 12.1, 3.5 Hz), 2.57 (1H, ddd, *J* = 16.2, 7.4, 4.0 Hz), 2.50 (1H, dd, *J* = 10.8, 6.4 Hz), 2.46–2.21 (3H, m), 2.13–2.03 (2H, m), 1.84 (1H, dd, *J* = 16.7, 6.7 Hz), 1.77 (1H, dd, *J* = 16.7, 4.7 Hz), 1.69 (1H, app. q, *J* = 11.5 Hz). ^{13}C NMR (151 MHz, C_6D_6) δ 206.3, 137.8, 135.9, 134.2, 134.0, 133.6, 131.7, 131.5, 130.3, 129.4, 128.4, 126.6, 126.59, 126.52, 126.46, 126.2, 125.9, 122.8, 116.4, 87.9, 85.0, 83.0, 82.6, 81.9, 81.7, 81.0, 80.6, 80.1, 78.5, 77.4, 75.3, 73.5, 68.6, 39.6, 37.6, 35.2, 32.7, 32.4, 21.6. $\nu_{max.}$ (film) 2977, 2928, 1719, 806 cm^{-1}. HRMS (APCI) calcd. for $C_{40}H_{44}NO_8$ $[M+H]^+$ 666.3061, found 666.3053.

X-Ray Data

Alcohol 21

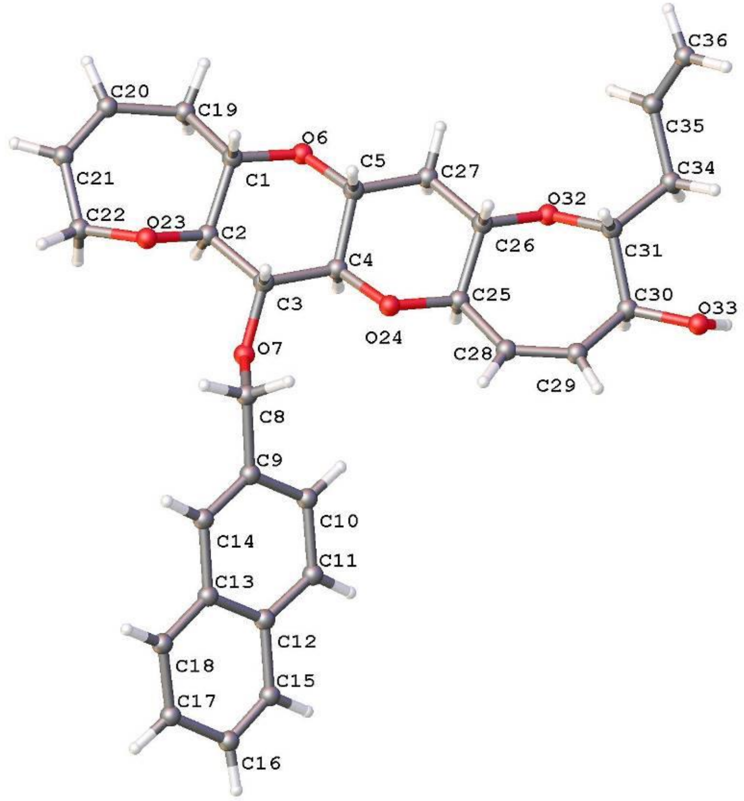

Crystal data

$C_{30}H_{34}O_6$	$F(000) = 524$
$M_r = 490.57$	$D_x = 1.096$ Mg m^{-3}
Monoclinic, $P2_1$	Cu *K*α radiation, λ = 1.54178 Å
a = 17.0450 (16) Å	Cell parameters from 7001 reflections
b = 4.6441 (4) Å	θ = 2.7–71.9°
c = 19.6969 (14) Å	μ = 0.61 mm^{-1}
β = 107.538 (4)°	*T* = 568 K
V = 1486.7 (2) Å^3	Column, colourless
Z = 2	0.36 × 0.06 × 0.02 mm

Data collection

Bruker D8 VENTURE diffractometer	3651 reflections with $I > 2\sigma(I)$
Radiation source: microfocus sealed tube, INCOATEC Iμs 3.0	$R_{int} = 0.087$
Multilayer mirror optics monochromator	$\theta_{max} = 74.8°$, $\theta_{min} = 2.7°$
Detector resolution: 7.4074 pixels mm^{-1}	$h = -21 \rightarrow 21$
φ and ω scans	$k = -5 \rightarrow 5$
26245 measured reflections	$l = -24 \rightarrow 24$
5935 independent reflections	

Refinement

Refinement on F^2	Hydrogen site location: inferred from neighbouring sites
Least-squares matrix: full	H-atom parameters constrained
$R[F^2 > 2\sigma(F^2)] = 0.081$	$w = 1/[\sigma^2(F_o^2) + (0.1784P)^2]$ where $P = (F_o^2 + 2F_c^2)/3$
$wR(F^2) = 0.281$	$(\Delta/\sigma)_{max} = 0.005$
$S = 1.01$	$\Delta\rangle_{max}$ = 0.19 e Å^{-3}
5935 reflections	$\Delta\rangle_{min}$ = −0.22 e Å^{-3}
327 parameters	Absolute structure: Refined as an inversion twin
312 restraints	Absolute structure parameter: 0.0 (5)

Ketone 30

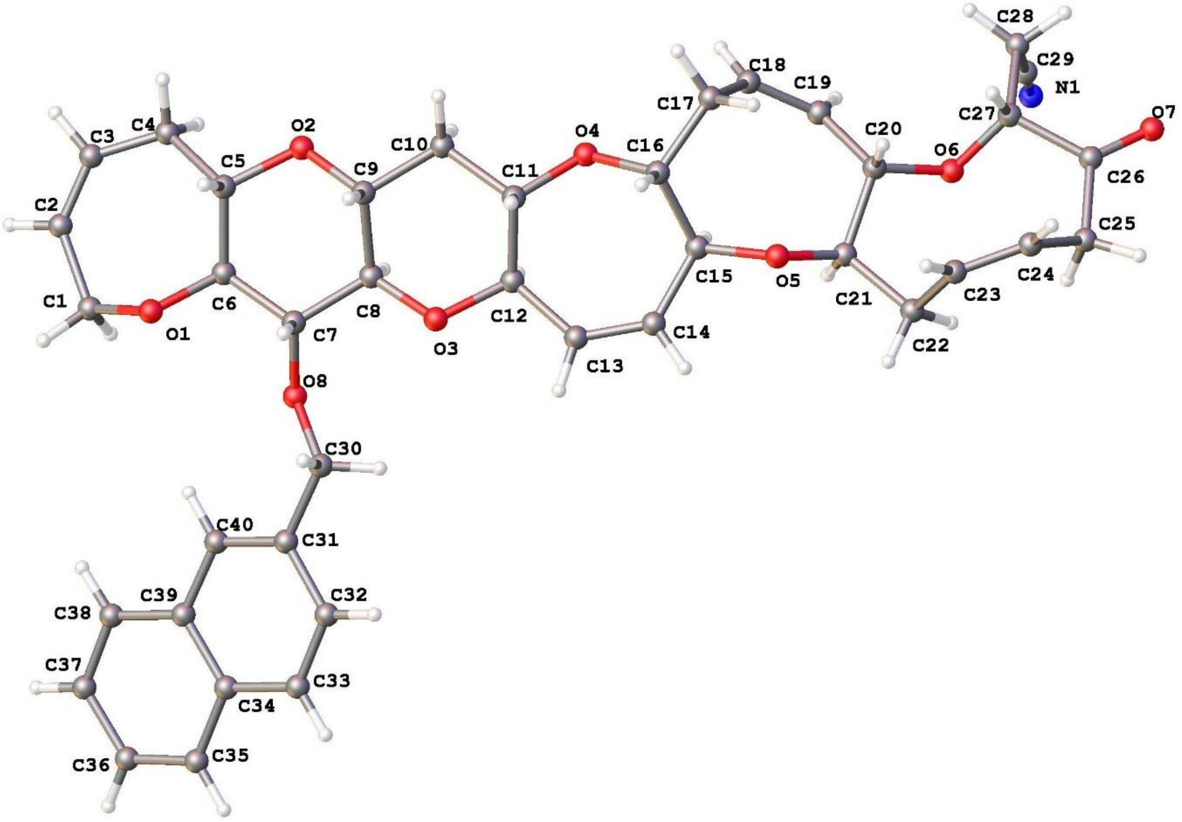

Crystal data

$C_{40}H_{43}NO_8$	D_x = 1.267 Mg m^{-3}
M_r = 665.75	Synchrotron radiation, λ = 0.6889 Å
Orthorhombic, $P2_12_12_1$	Cell parameters from 6890 reflections
a = 8.3895 (3) Å	θ = 2.0–25.5°
b = 10.3586 (6) Å	μ = 0.08 mm^{-1}
c = 40.1762 (14) Å	*T* = 100 K
V = 3491.4 (3) Å^3	Rod, colourless
Z = 4	0.14 × 0.01 × 0.01 mm
F(000) = 1416	

Data collection

Fluid Film Devices diffractometer	7141 independent reflections
Radiation source: Synchrotron, Undulator, I19, DLS, RAL	3087 reflections with $I > 2\sigma(I)$
Silicon 111 monochromator	R_{int} = 0.342
Detector resolution: 5.814 pixels mm^{-1}	θ_{max} = 25.5°, θ_{min} = 2.0°
Shutterless φ and ω scans	*h* = −10 → 10
Absorption correction: multi-scan *CrysAlis PRO* 1.171.42.94a (Rigaku Oxford Diffraction, 2023). Empirical absorption correction using spherical harmonics, implemented in SCALE3 ABSPACK scaling algorithm.	*k* = −12 → 12
T_{min} = 0.049, T_{max} = 1.000	*l* = −50 → 50
93629 measured reflections	

Refinement

Refinement on F^2	Hydrogen site location: inferred from neighbouring sites
Least-squares matrix: full	H-atom parameters constrained
$R[F^2 > 2\sigma(F^2)]$ = 0.085	$w = 1/[\sigma^2(F_o^2) + (0.0928P)^2]$ where $P = (F_o^2 + 2F_c^2)/3$
$wR(F^2)$ = 0.253	$(\Delta/\sigma)_{max}$ < 0.001
S = 0.98	$\Delta\rangle_{max}$ = 0.32 e Å^{-3}
7141 reflections	$\Delta\rangle_{min}$ = −0.26 e Å^{-3}
442 parameters	Absolute structure: Flack x determined using 876 quotients [(I+)− (I−)]/[(I+)+(I−)] (S. Parsons, H. D. Flack and T. Wagner, *Acta Cryst. B* **2013**, *69*, 249−259).
0 restraints	Absolute structure parameter: 0.6 (10)

References

1. M. Fétizon, D. D. Khac, N. D. Tho, *Tetrahedron Lett.* **1986**, *27*, 1777−1780.
2. S. Yamashita, K. Takeuchi, T. Koyama, M. Inoue, Y. Hayashi, M. Hirama, *Chem. Eur. J.* **2015**, *21*, 2621−2628.
3. M. Inoue, S. Yamashita, Y. Ishihara, M. Hirama, *Org. Lett.* **2006**, *8*, 5805−5808.
4. R. F. Hudson, P.A. Chopard, *J. Org. Chem.* **1963**, *28*, 2446−2447.
5. S. Kobayashi, Y. Takahashi, K. Komano, B. H. Alizadeh, Y. Kawada, T. Oishi, S. Tanaka, Y. Ogasawara, S. Sasaki, M. Hirama, *Tetrahedron* **2004**, *60*, 8375−8396.

^{1}H and ^{13}C NMR Spectra

	Page
^{1}H NMR Spectrum of **2** (500 MHz, $CDCl_3$)	S35
^{13}C NMR Spectrum of **2** (126 MHz, $CDCl_3$)	S36
^{1}H NMR Spectrum of **3** (400 MHz, $CDCl_3$)	S37
^{13}C NMR Spectrum of **3** (101 MHz, $CDCl_3$)	S38
^{1}H NMR Spectrum of **4** (400 MHz, $CDCl_3$)	S39
^{13}C NMR Spectrum of **4** (101 MHz, $CDCl_3$)	S40
^{1}H NMR Spectrum of **5** (400 MHz, $CDCl_3$)	S41
^{13}C NMR Spectrum of **5** (101 MHz, $CDCl_3$)	S42
^{1}H NMR Spectrum of **6** (400 MHz, $CDCl_3$)	S43
^{13}C NMR Spectrum of **6** (101 MHz, $CDCl_3$)	S44
^{1}H NMR Spectrum of **S1** (400 MHz, $CDCl_3$)	S45
^{13}C NMR Spectrum of **S1** (101 MHz, $CDCl_3$)	S46
^{1}H NMR Spectrum of **8** (400 MHz, $CDCl_3$)	S47
^{13}C NMR Spectrum of **8** (101 MHz, $CDCl_3$)	S48
^{1}H NMR Spectrum of **9** (400 MHz, $CDCl_3$)	S49
^{13}C NMR Spectrum of **9** (101 MHz, $CDCl_3$)	S50
^{1}H NMR Spectrum of **10** (400 MHz, $CDCl_3$)	S51
^{13}C NMR Spectrum of **10** (101 MHz, $CDCl_3$)	S52
^{1}H NMR Spectrum of **12** (500 MHz, $CDCl_3$)	S53
^{13}C NMR Spectrum of **12** (126 MHz, $CDCl_3$)	S54
^{1}H NMR Spectrum of **13** (500 MHz, $CDCl_3$)	S55
^{13}C NMR Spectrum of **13** (126 MHz, $CDCl_3$)	S56
^{1}H NMR Spectrum of **14** (400 MHz, $CDCl_3$)	S57
^{13}C NMR Spectrum of **14** (101 MHz, $CDCl_3$)	S58
^{1}H NMR Spectrum of **11** (500 MHz, $CDCl_3$)	S59
^{13}C NMR Spectrum of **11** (126 MHz, $CDCl_3$)	S60
^{1}H NMR Spectrum of **15a** (400 MHz, $CDCl_3$)	S61
^{13}C NMR Spectrum of **15a** (101 MHz, $CDCl_3$)	S62
^{1}H NMR Spectrum of **17** (400 MHz, $CDCl_3$)	S63
^{13}C NMR Spectrum of **17** (101 MHz, $CDCl_3$)	S64
^{1}H NMR Spectrum of **18** (400 MHz, $CDCl_3$)	S65
^{13}C NMR Spectrum of **18** (101 MHz, $CDCl_3$)	S66
^{1}H NMR Spectrum of **19** (500 MHz, $CDCl_3$)	S67

^{13}C NMR Spectrum of **19** (126 MHz, $CDCl_3$) S68
^{1}H NMR Spectrum of **20** (400 MHz, $CDCl_3$) S69
^{13}C NMR Spectrum of **20** (101 MHz, $CDCl_3$) S70
^{1}H NMR Spectrum of **21** (500 MHz, $CDCl_3$) S71
^{13}C NMR Spectrum of **21** (126 MHz, $CDCl_3$) S72
^{1}H NMR Spectrum of **22** (400 MHz, $CDCl_3$) S73
^{13}C NMR Spectrum of **22** (101 MHz, $CDCl_3$) S74
^{1}H NMR Spectrum of **23** (400 MHz, $CDCl_3$) S75
^{13}C NMR Spectrum of **23** (101 MHz, $CDCl_3$) S76
^{1}H NMR Spectrum of **24** (500 MHz, $CDCl_3$) S77
^{13}C NMR Spectrum of **24** (126 MHz, $CDCl_3$) S78
^{1}H NMR Spectrum of **25** (400 MHz, $CDCl_3$) S79
^{13}C NMR Spectrum of **25** (101 MHz, $CDCl_3$) S80
^{1}H NMR Spectrum of **26** (400 MHz, $CDCl_3$) S81
^{13}C NMR Spectrum of **26** (101 MHz, $CDCl_3$) S82
^{1}H NMR Spectrum of **S3** (500 MHz, $CDCl_3$) S83
^{13}C NMR Spectrum of **S3** (126 MHz, $CDCl_3$) S84
^{1}H NMR Spectrum of **27** (500 MHz, $CDCl_3$) S85
^{13}C NMR Spectrum of **27** (126 MHz, $CDCl_3$) S86
^{1}H NMR Spectrum of **28** (500 MHz, $CDCl_3$) S87
^{13}C NMR Spectrum of **28** (126 MHz, $CDCl_3$) S88
^{1}H NMR Spectrum of **S4a,b** (600 MHz, C_6D_6) S89
^{13}C NMR Spectrum of **S4a,b** (151 MHz, C_6D_6) S90
^{1}H NMR Spectrum of **30** (400 MHz, C_6D_6) S91
^{13}C NMR Spectrum of **30** (151 MHz, C_6D_6) S92

^{1}H NMR (500 MHz, $CDCl_3$)

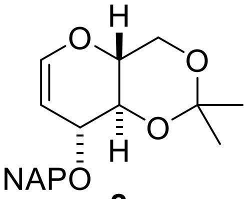

2

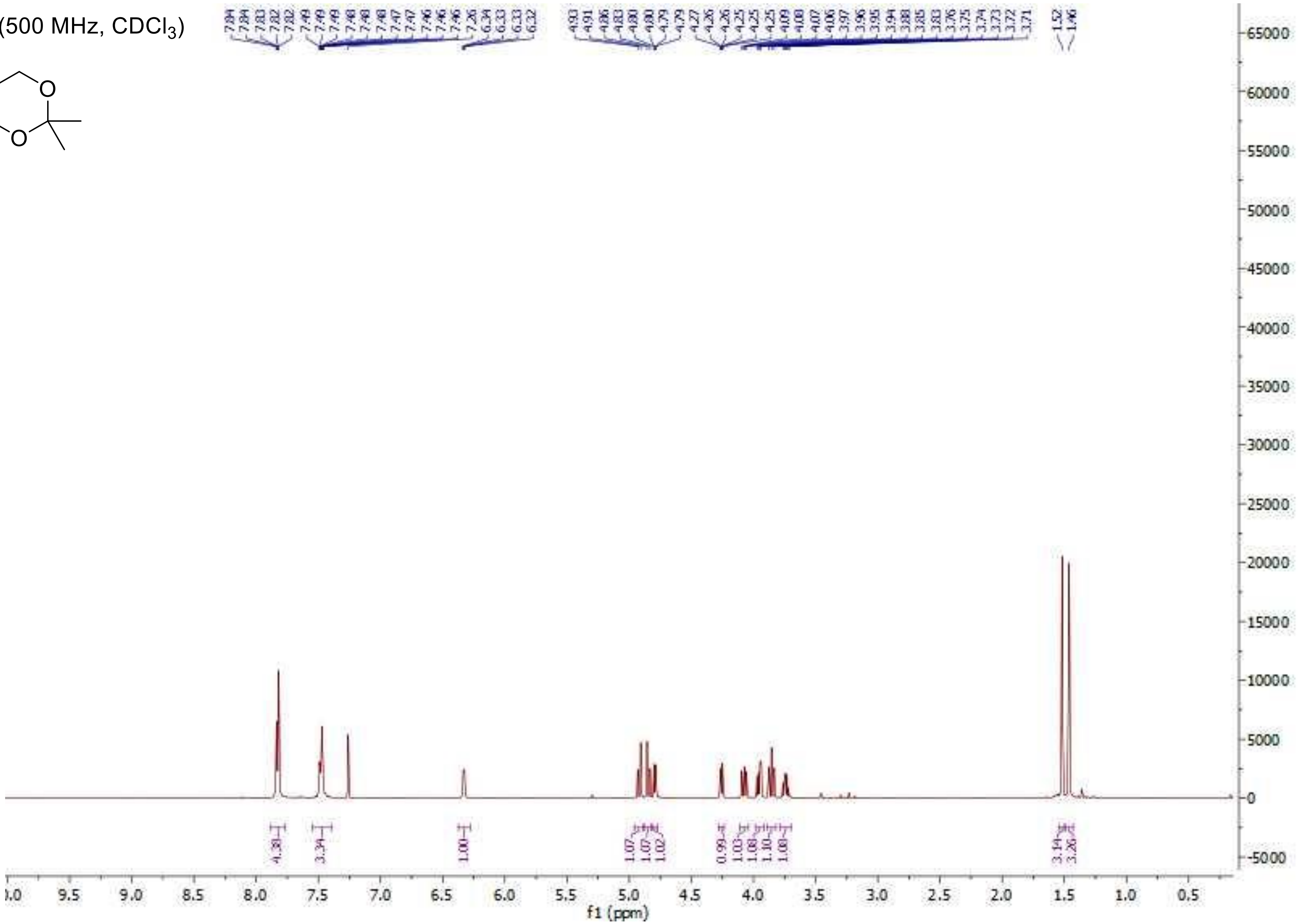

^{13}C NMR (126 MHz, $CDCl_3$)

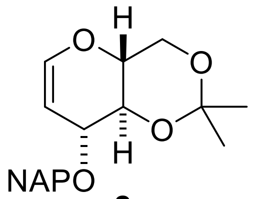

2

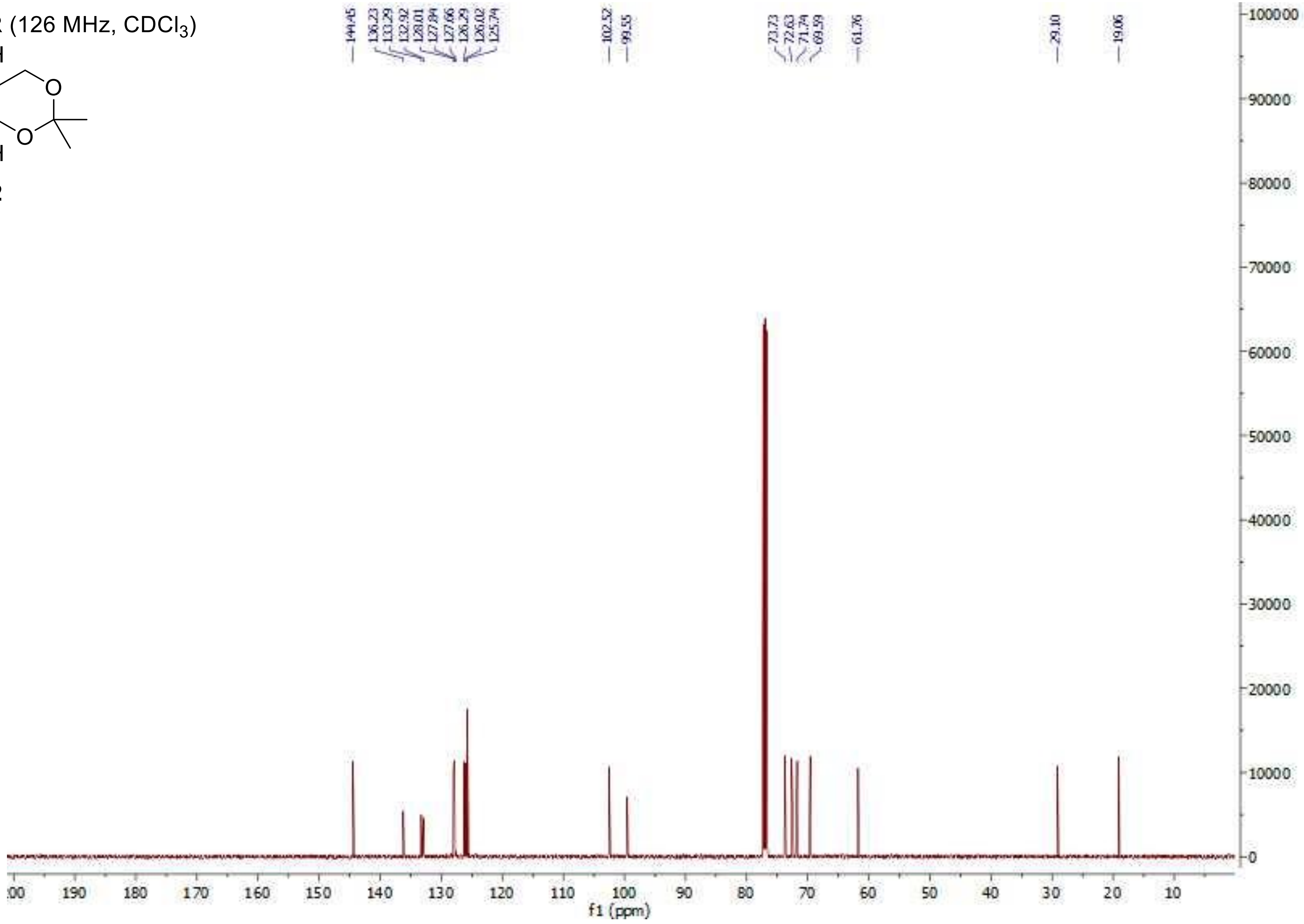

^{1}H NMR (400 MHz, $CDCl_3$)

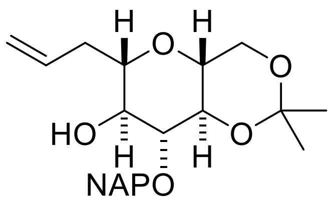

3

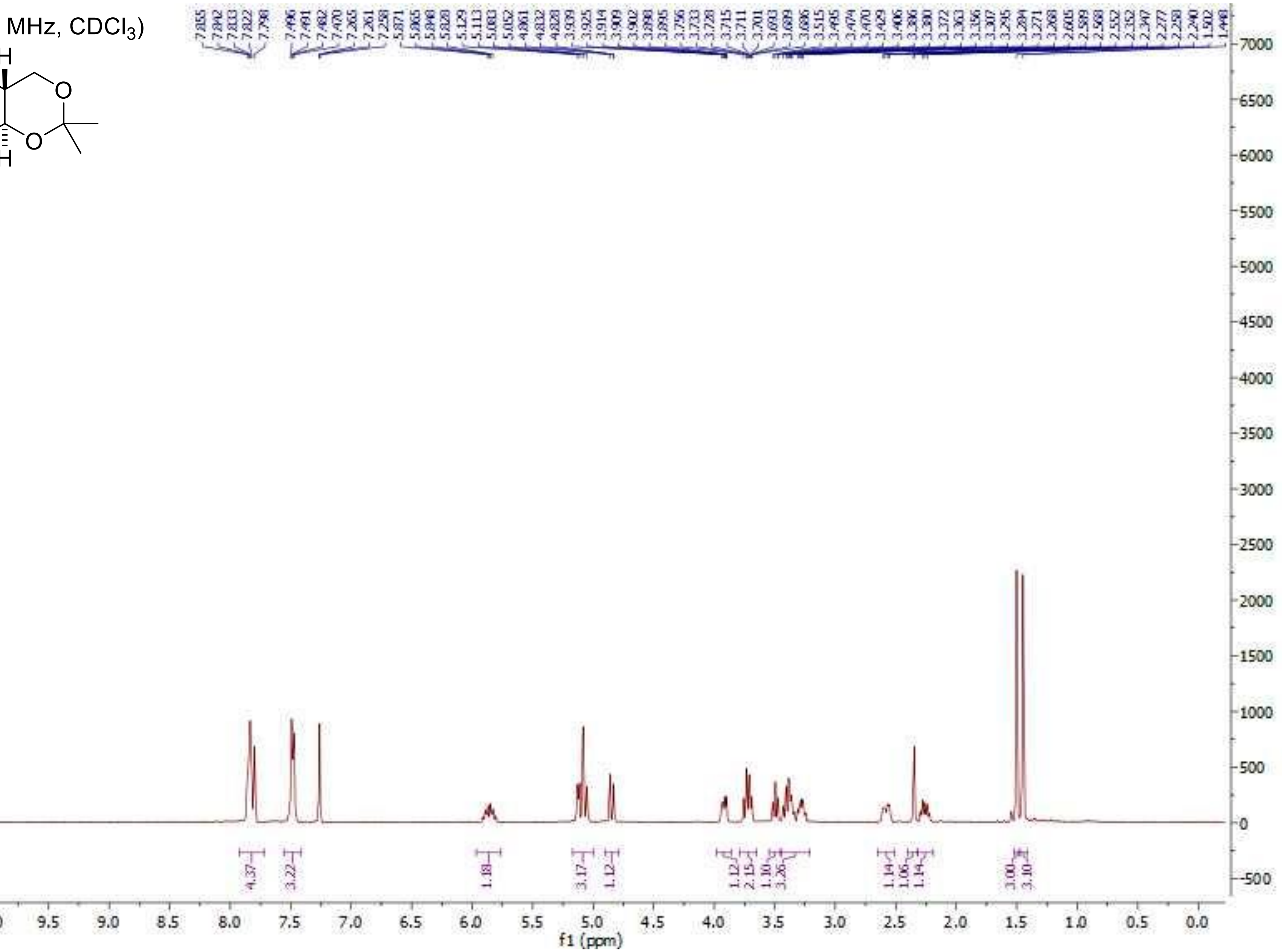

^{13}C NMR (101 MHz, $CDCl_3$)

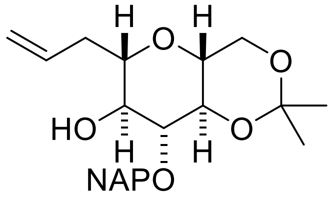

3

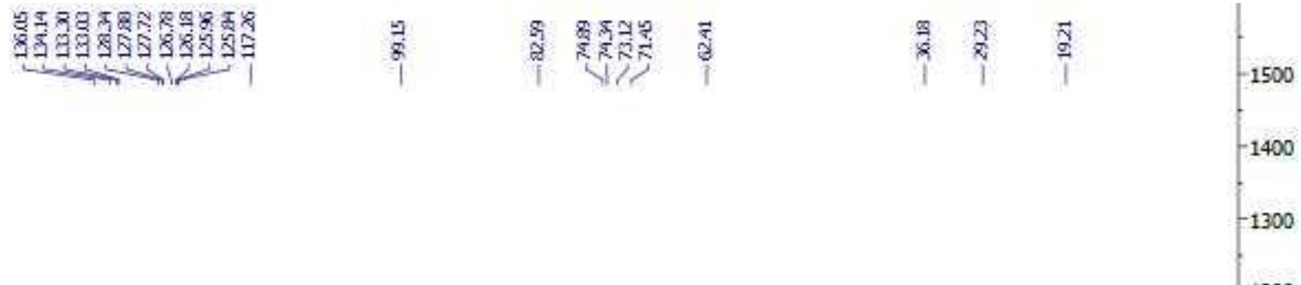

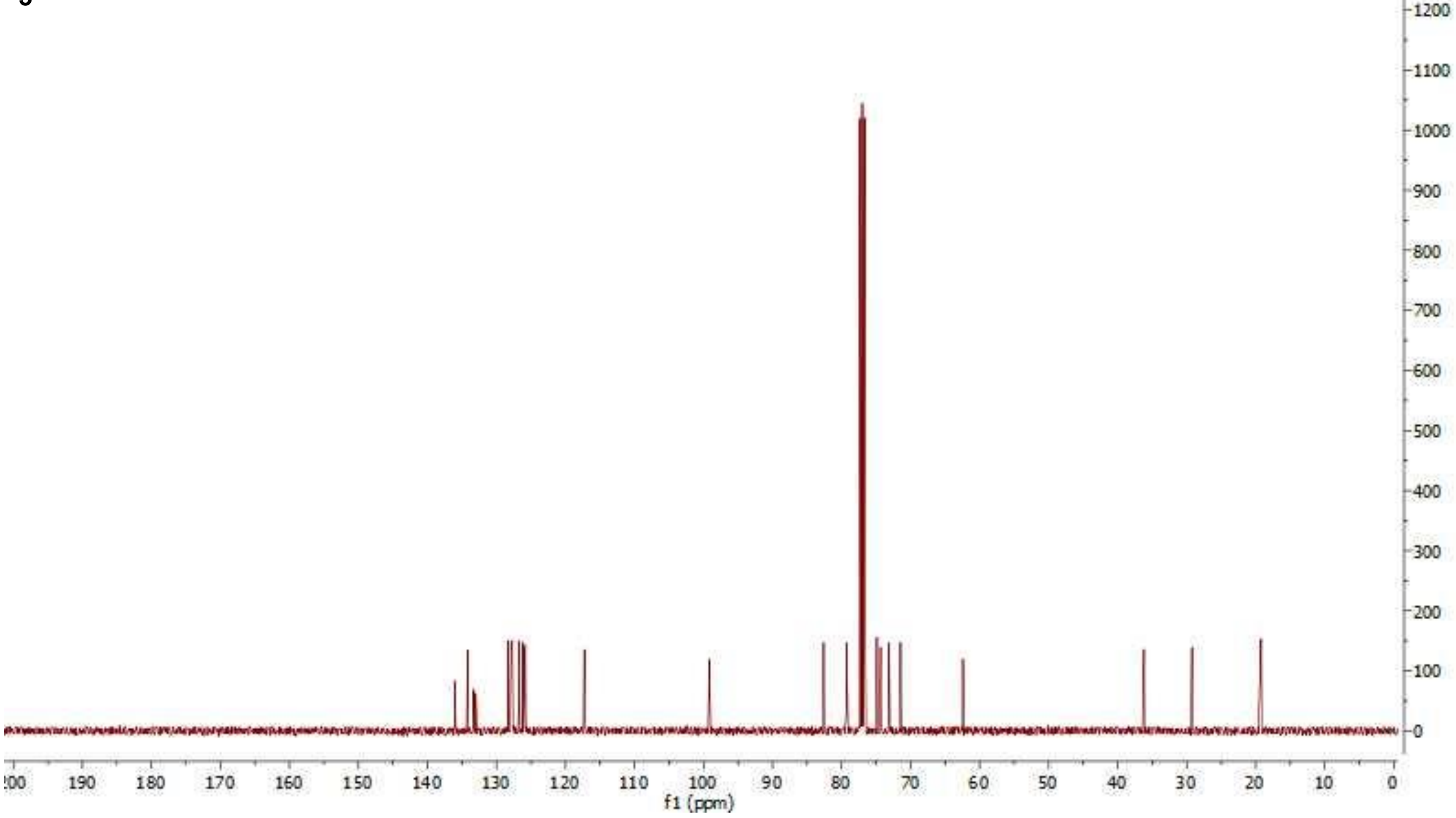

^1H NMR (400 MHz, $CDCl_3$)

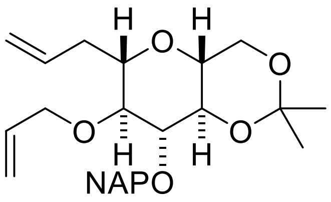

4

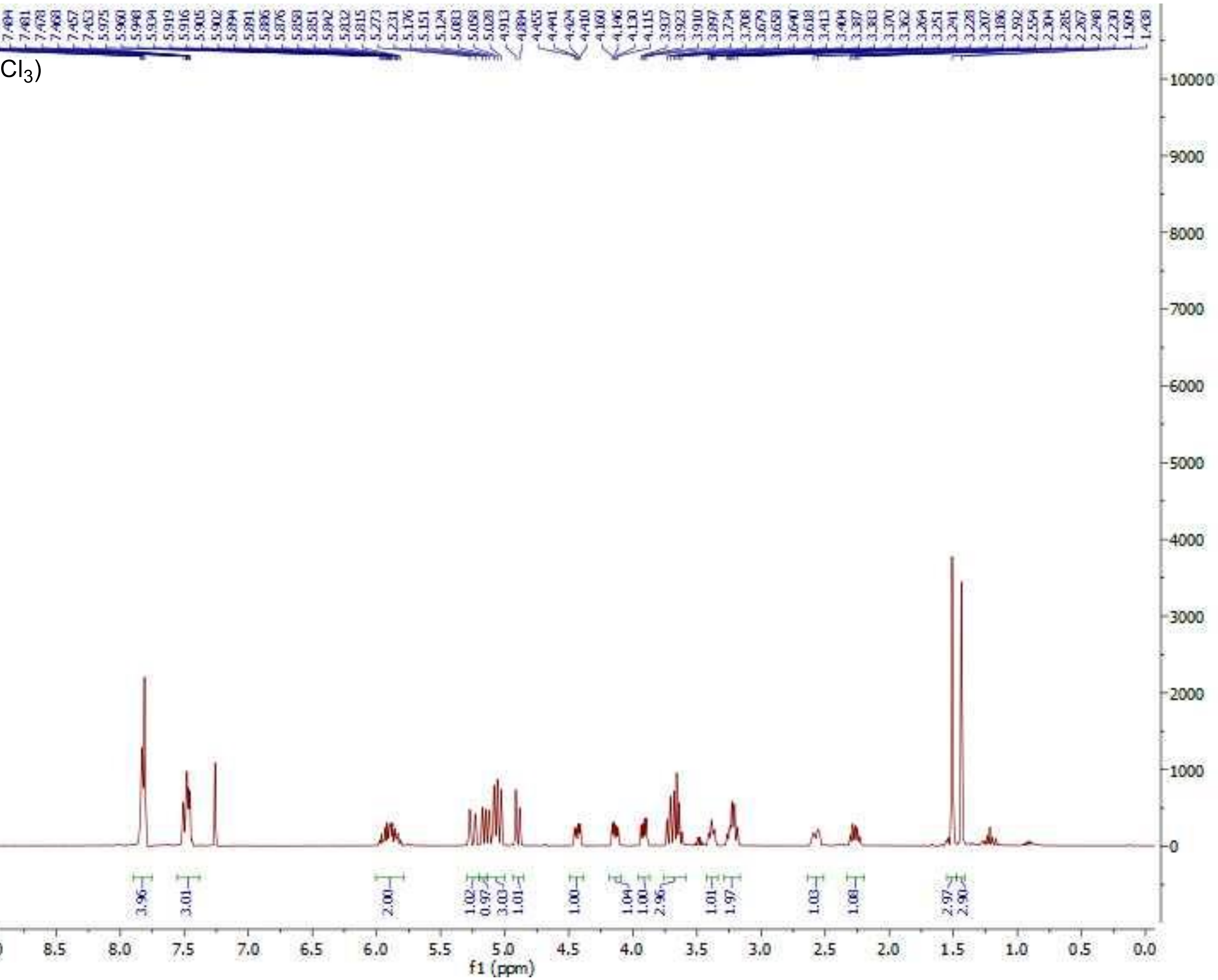

^{13}C NMR (101 MHz, $CDCl_3$)

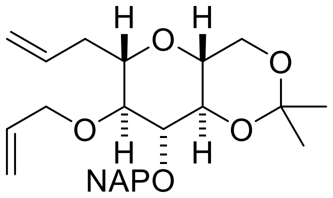

4

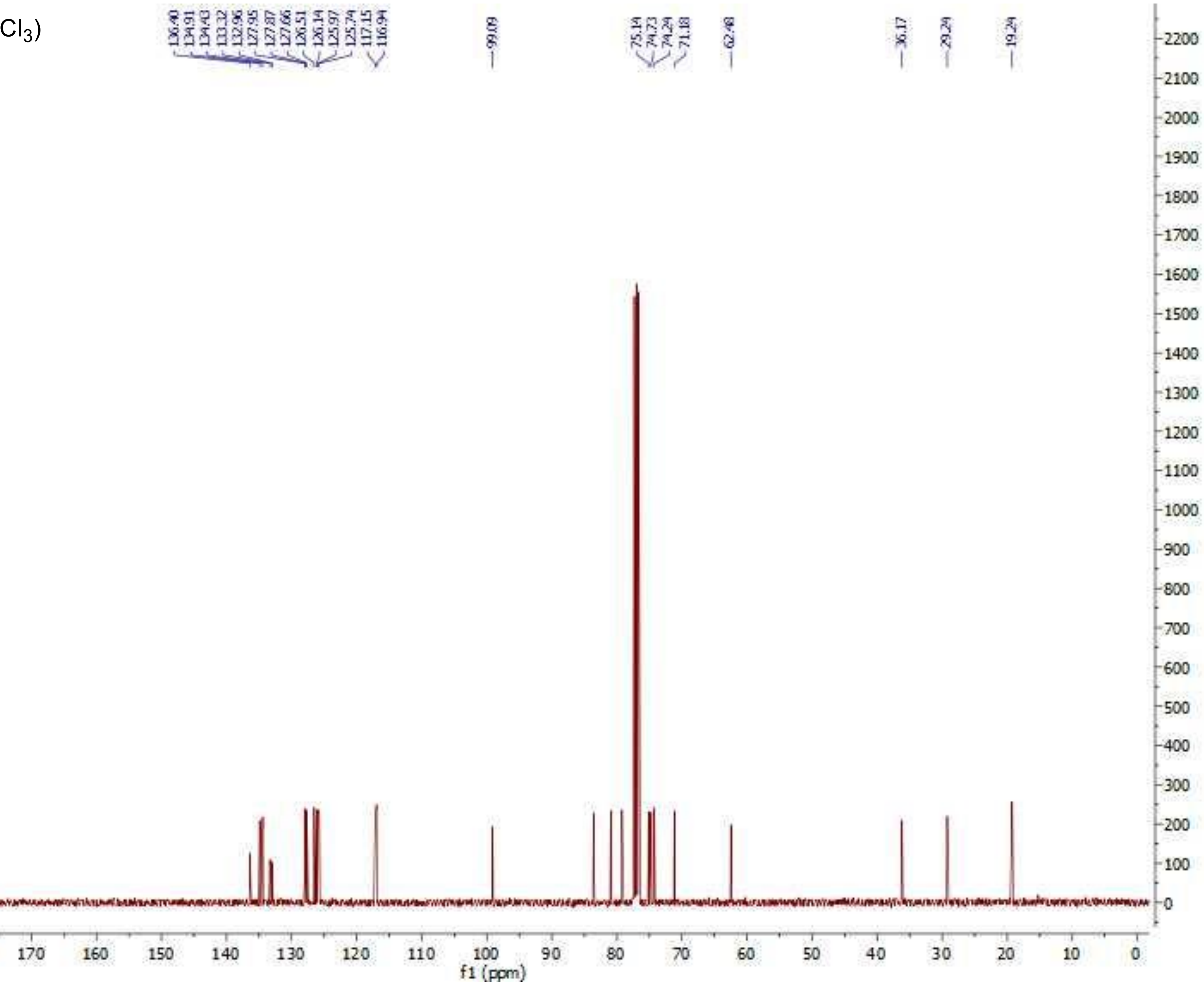

^1H NMR (400 MHz, $CDCl_3$)

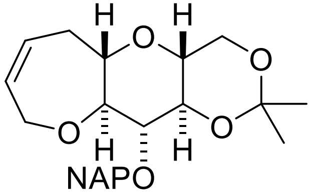

5

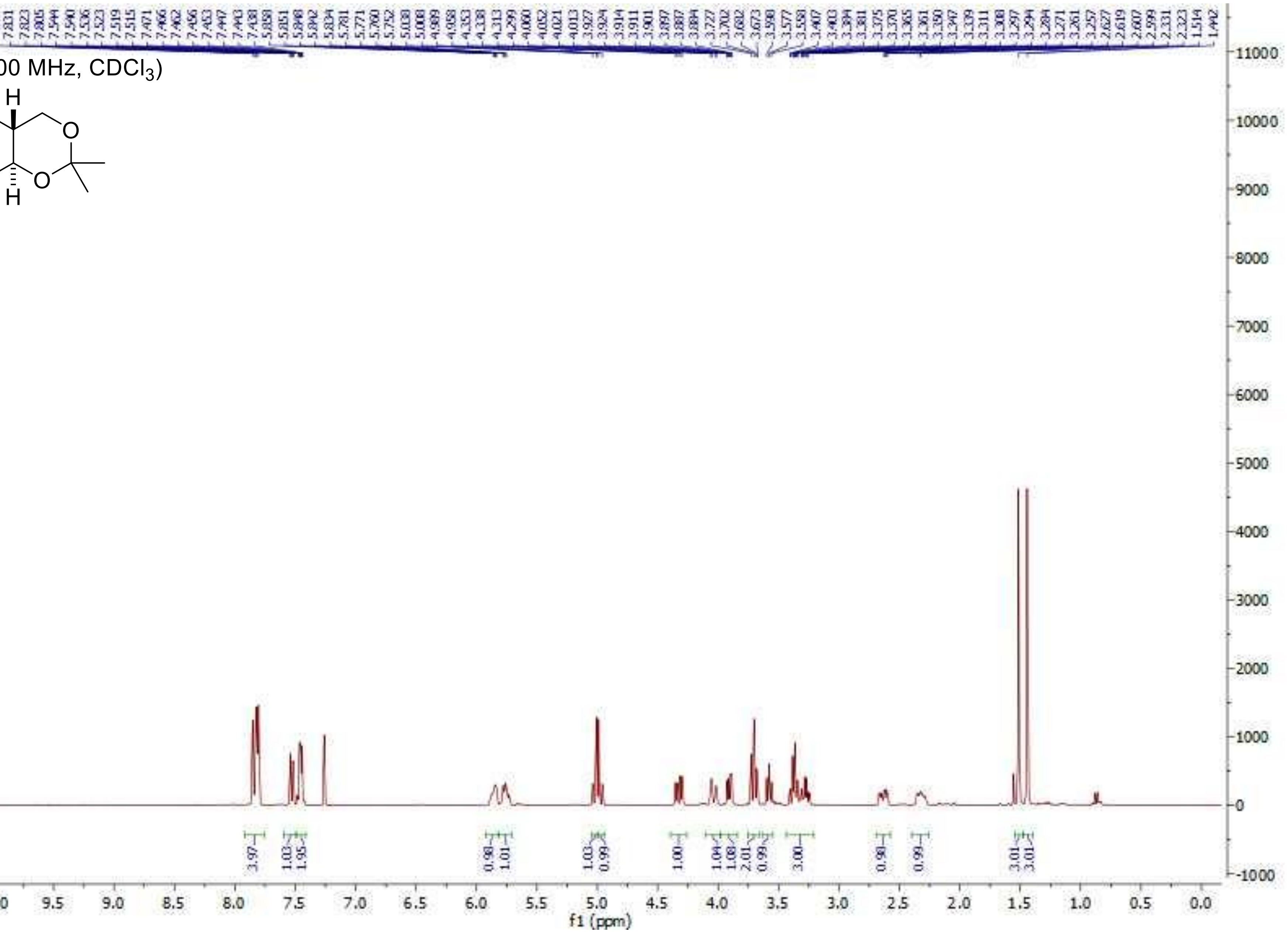

^{13}C NMR (101 MHz, $CDCl_3$)

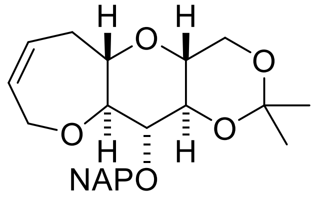

5

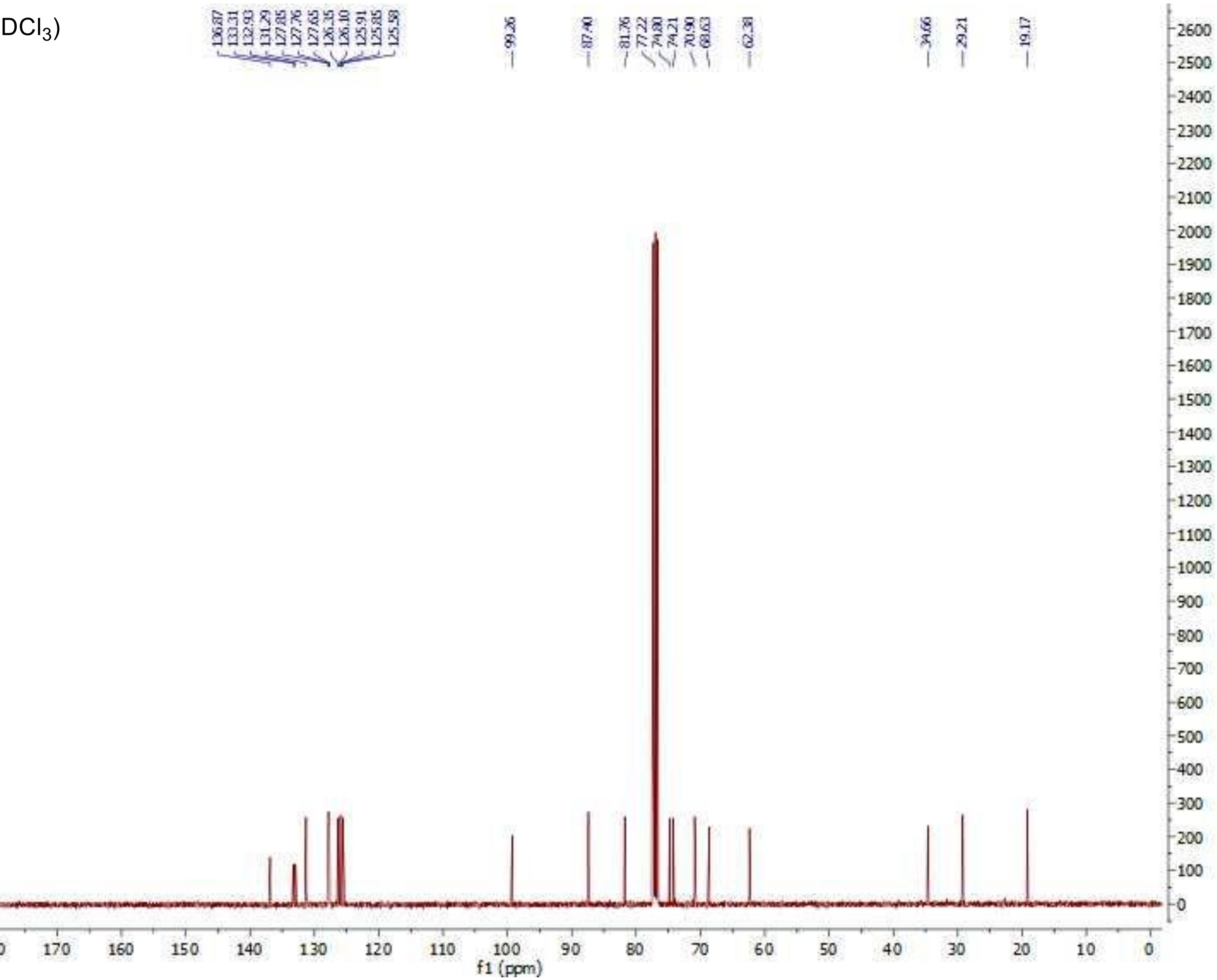

^{1}H NMR (400 MHz, $CDCl_3$)

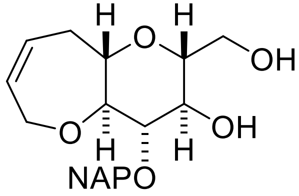

6

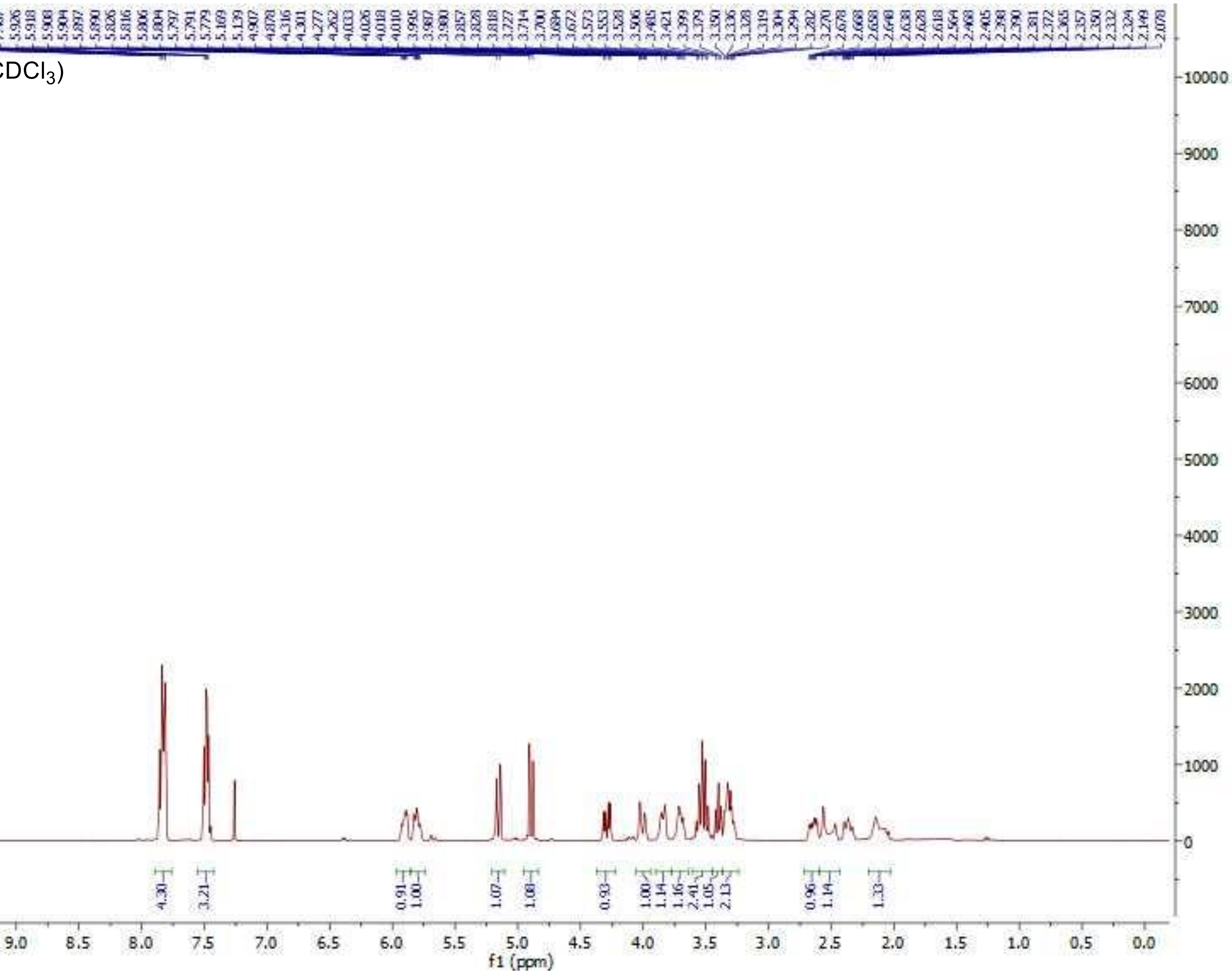

^{13}C NMR (101 MHz, $CDCl_3$)

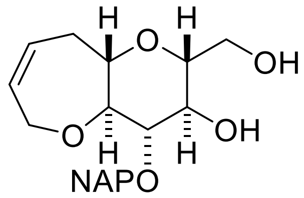

6

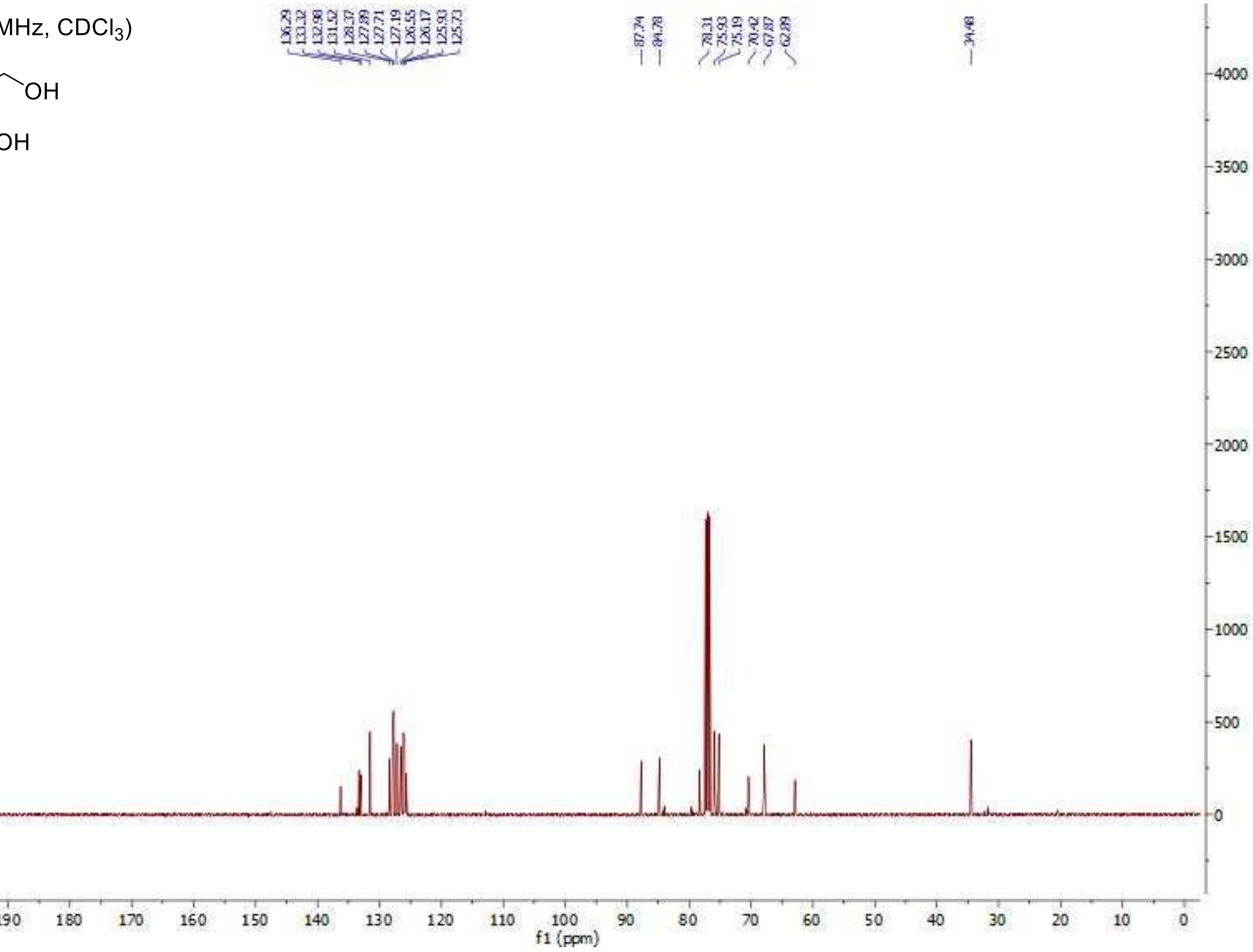

^{1}H NMR (400 MHz, $CDCl_3$)

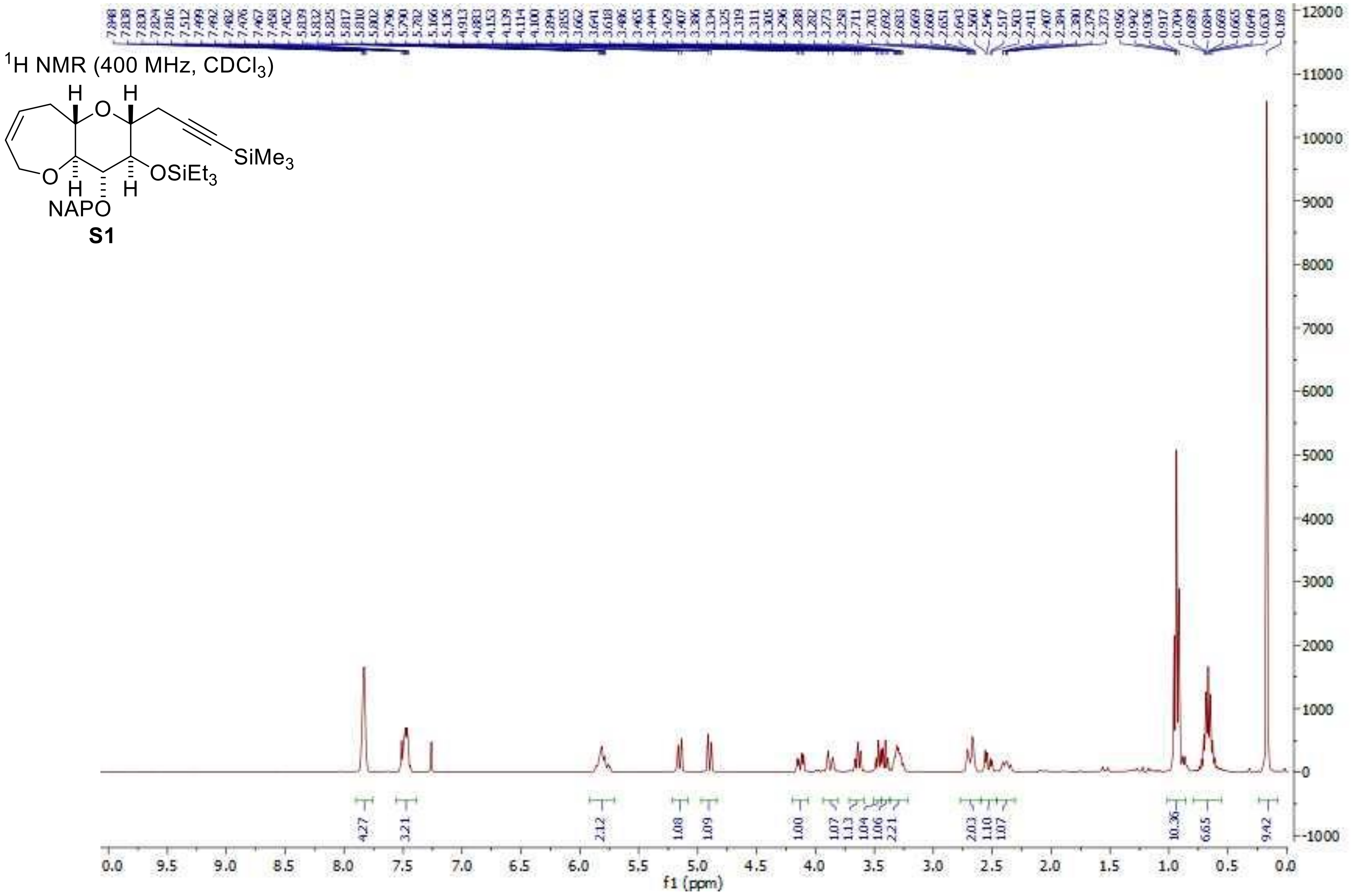

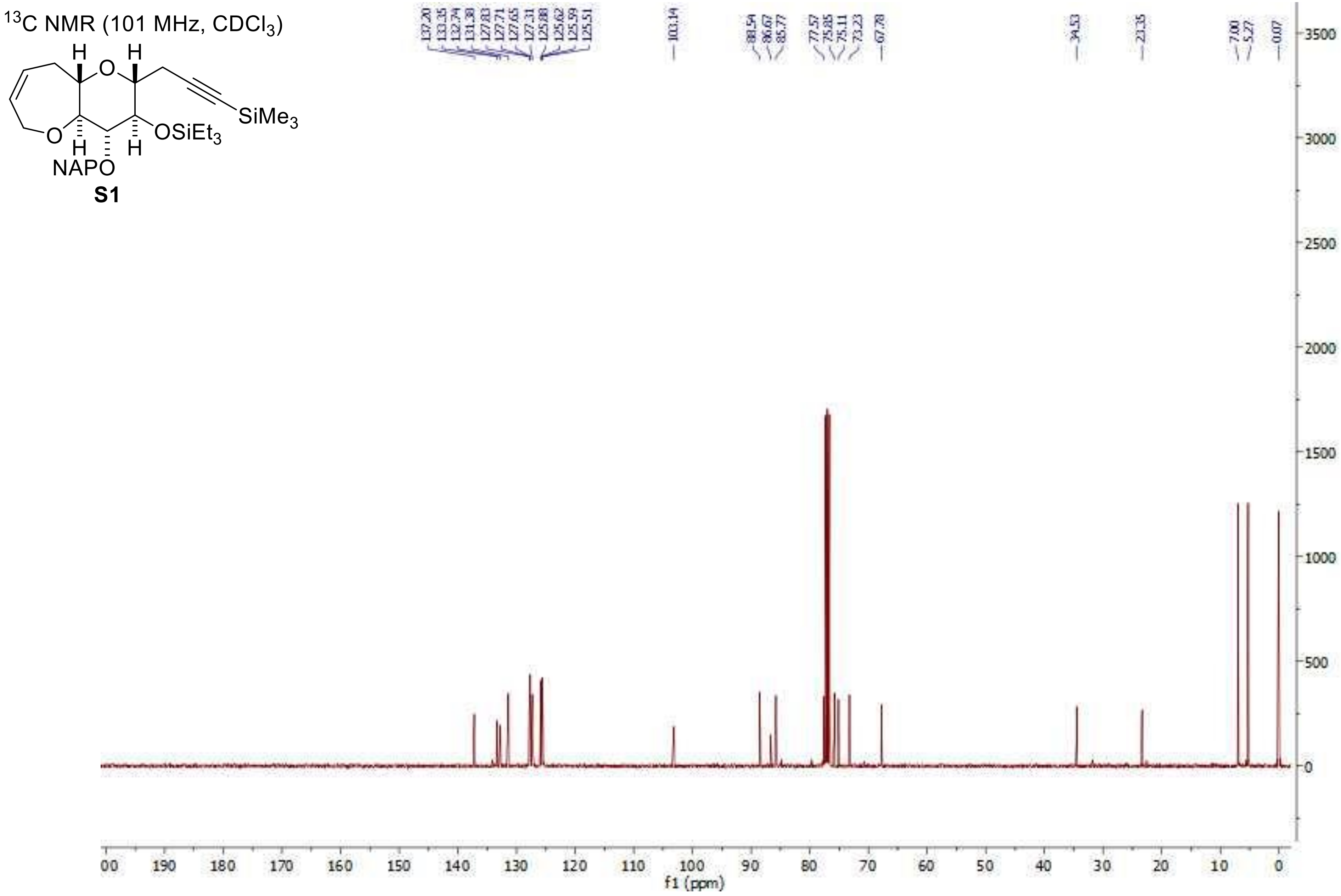

^{13}C NMR (101 MHz, CDCl$_3$)
SiMe$_3$
OSiEt$_3$
NAPO
S1
137.20
133.35
132.74
131.38
127.83
127.71
127.65
127.31
125.88
125.62
125.59
125.51
103.14
88.54
86.67
85.77
77.57
75.85
75.11
73.23
67.78
34.53
23.35
7.00
5.27
0.07
f1 (ppm)

^{1}H NMR (400 MHz, $CDCl_3$)

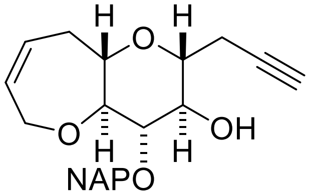

8

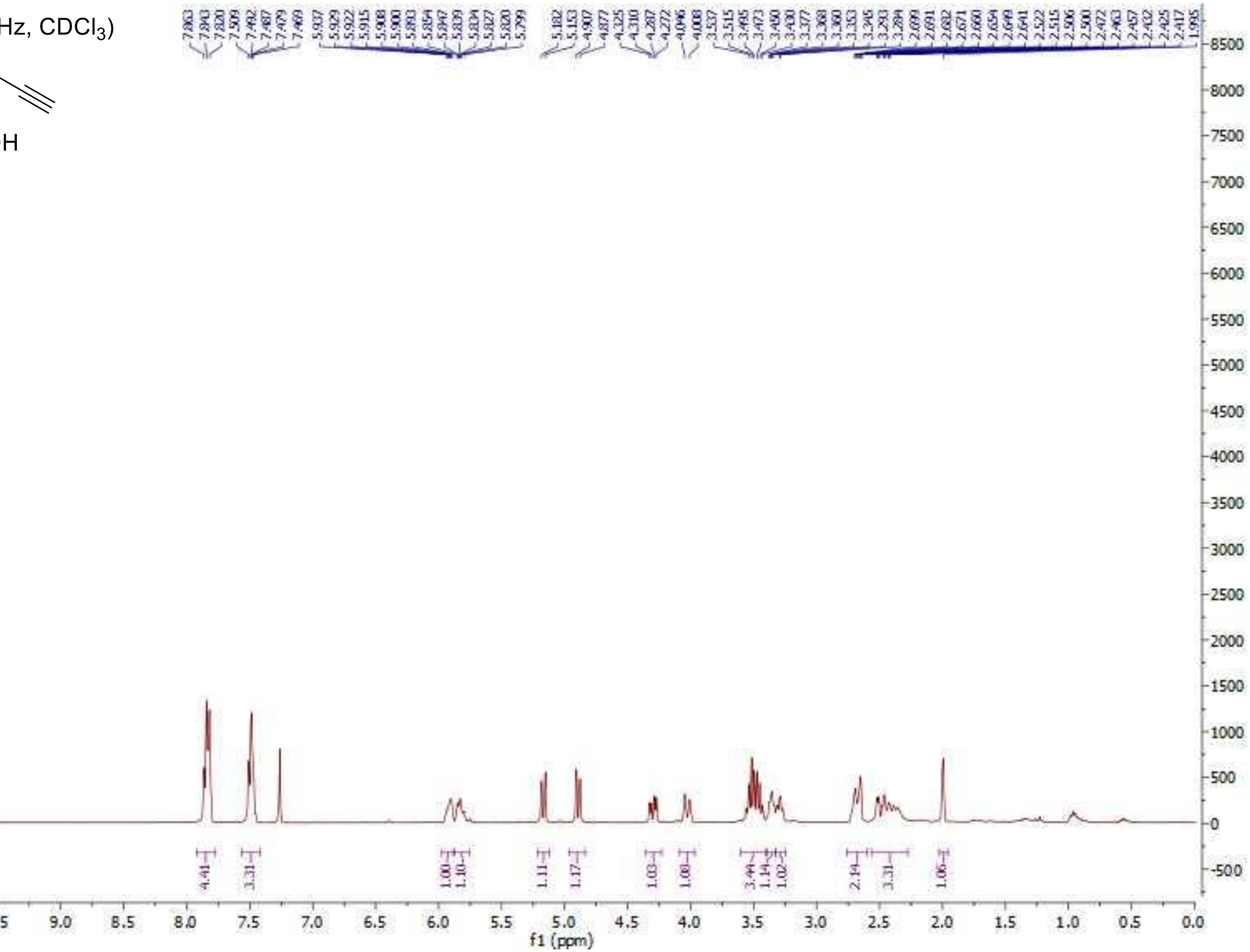

^{13}C NMR (101 MHz, $CDCl_3$)

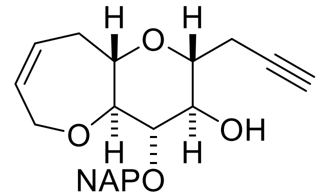

8

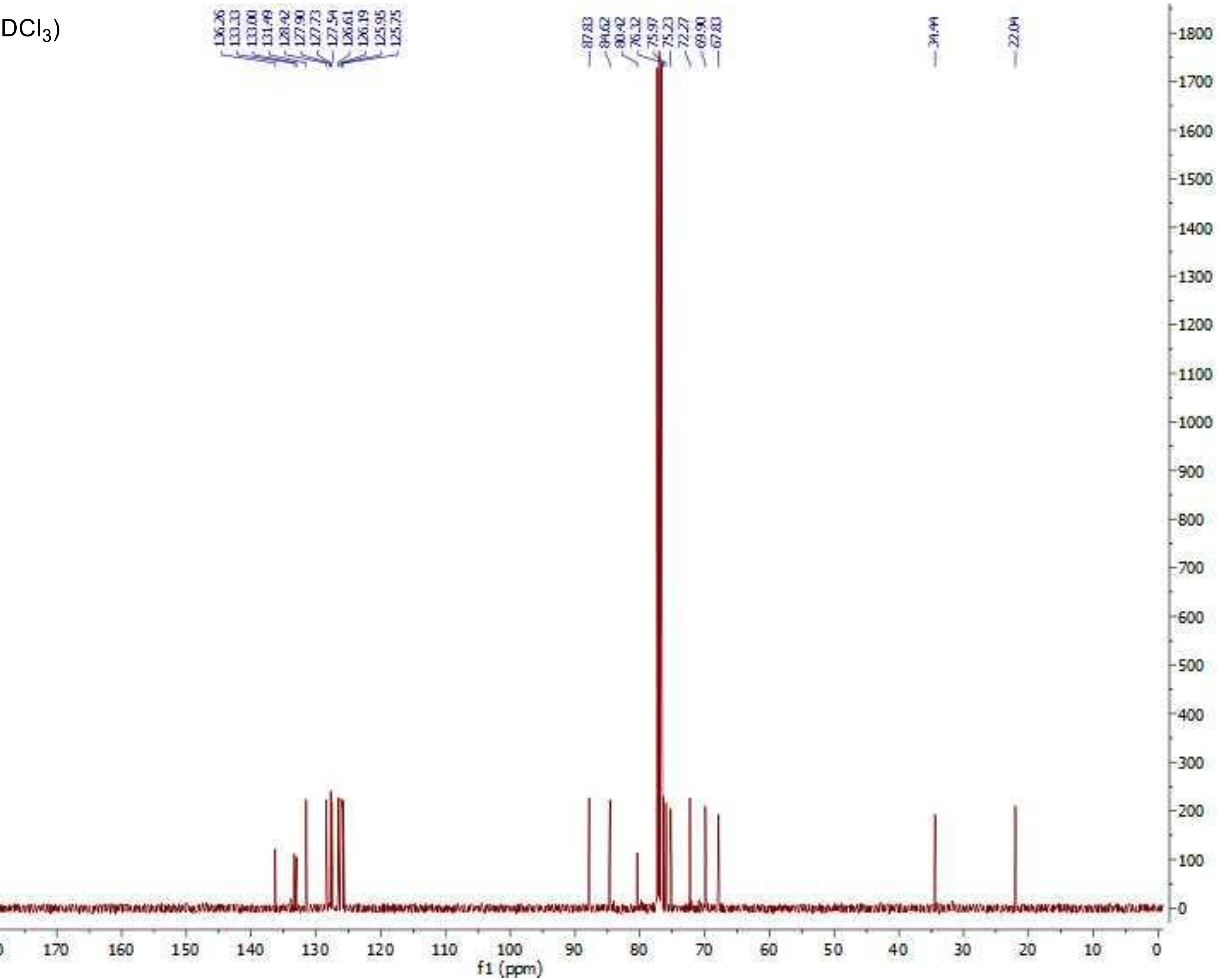

^{1}H NMR (400 MHz, $CDCl_3$)

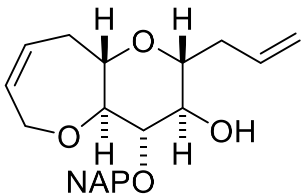

9

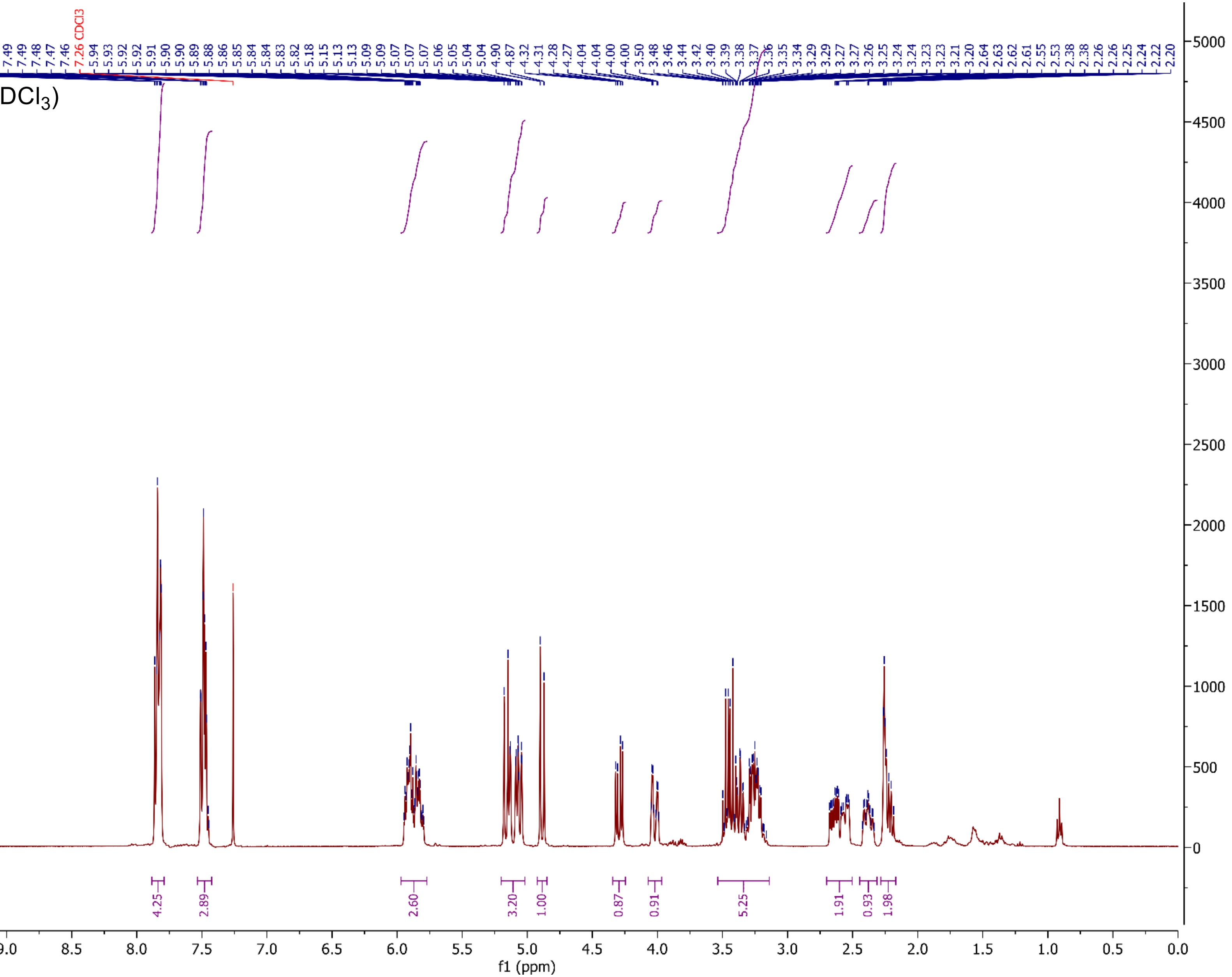

^{13}C NMR (101 MHz, $CDCl_3$)

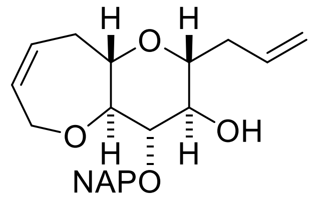

9

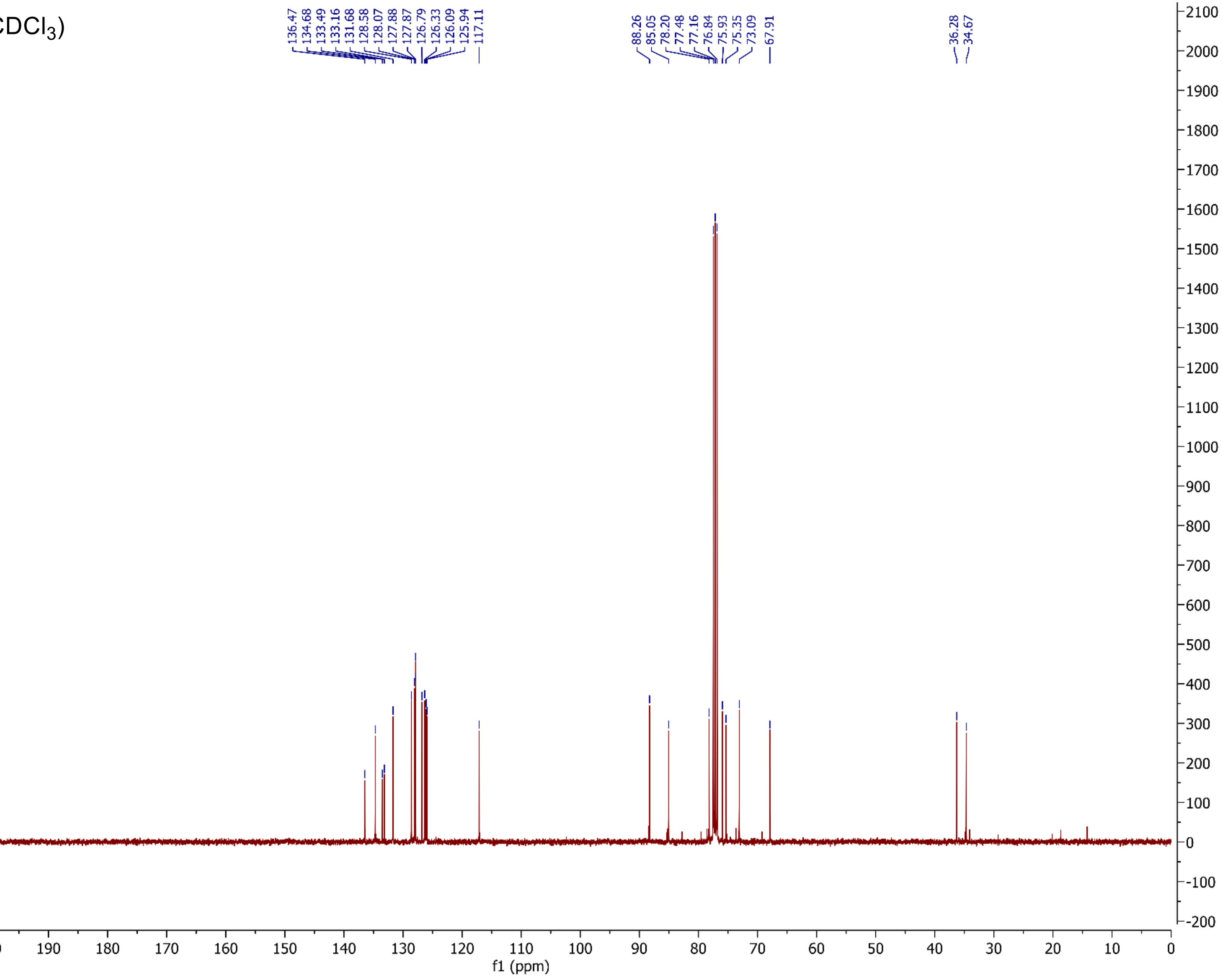

^1H NMR (400 MHz, $CDCl_3$)

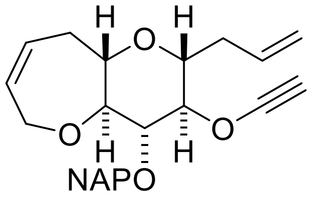

10

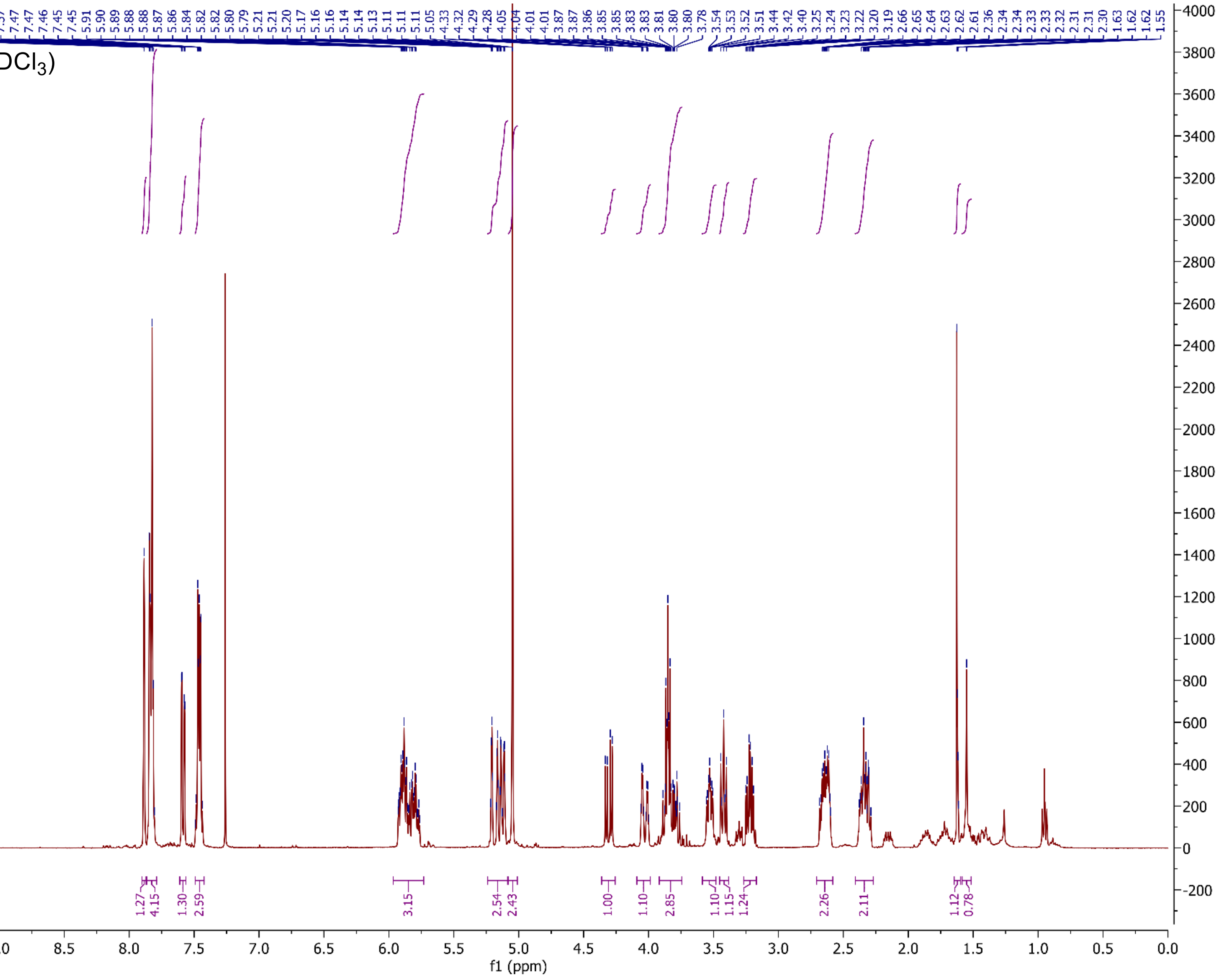

^{13}C NMR (101 MHz, $CDCl_3$)

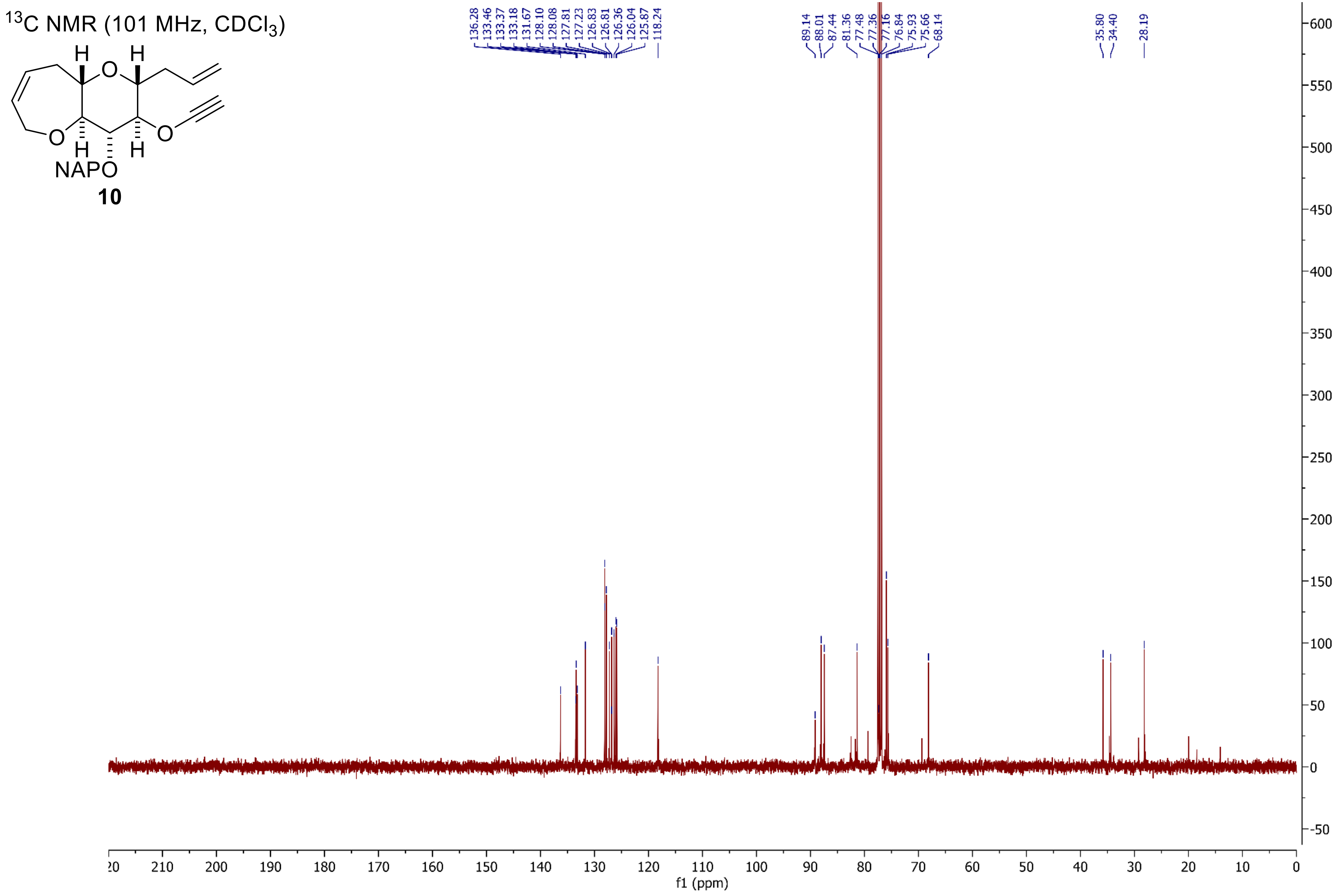

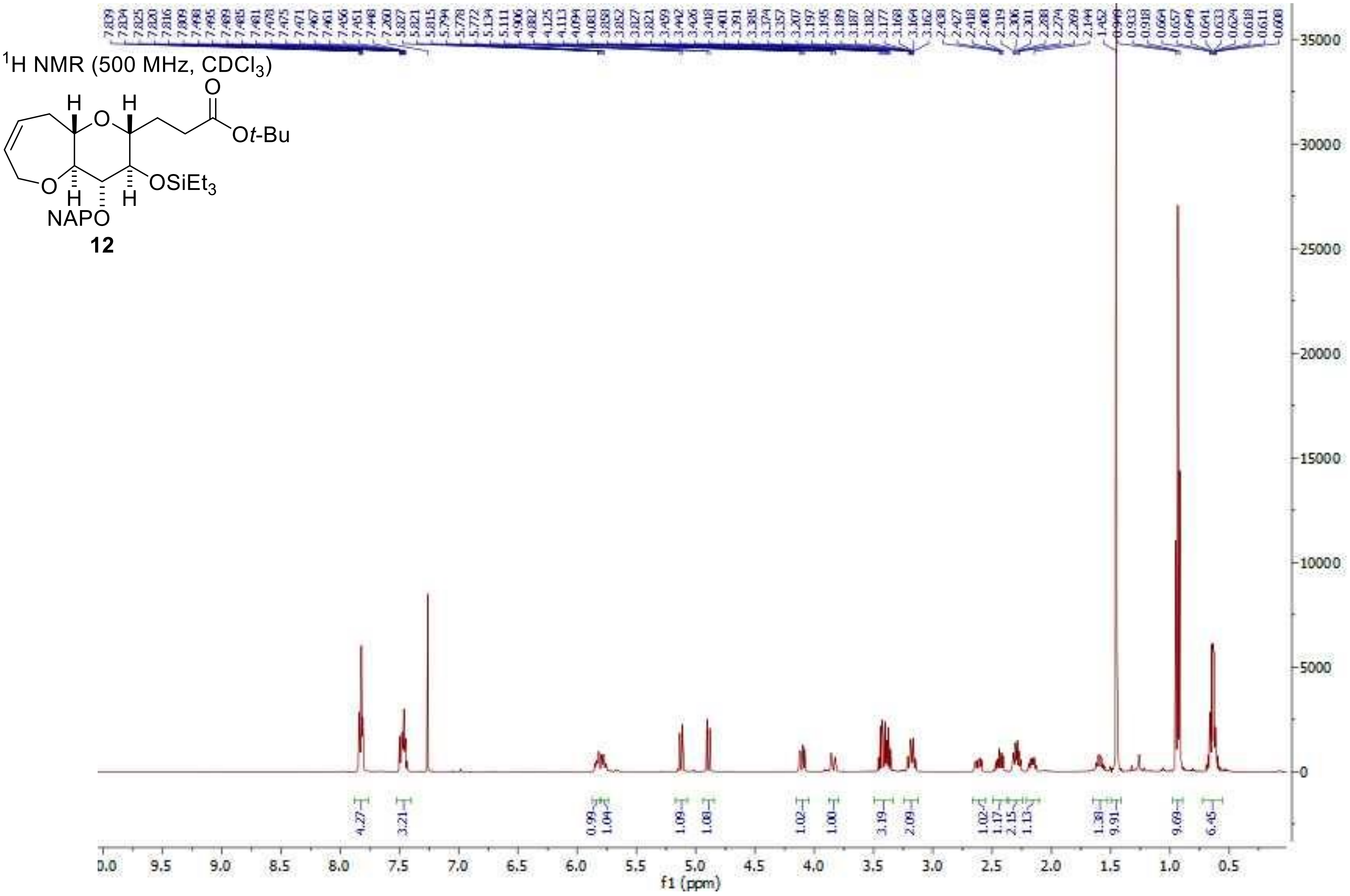

12

^{13}C NMR (126 MHz, $CDCl_3$)

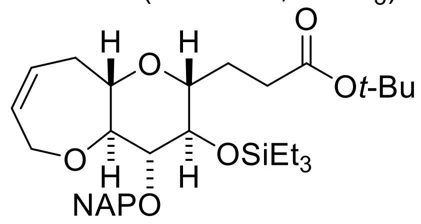

12

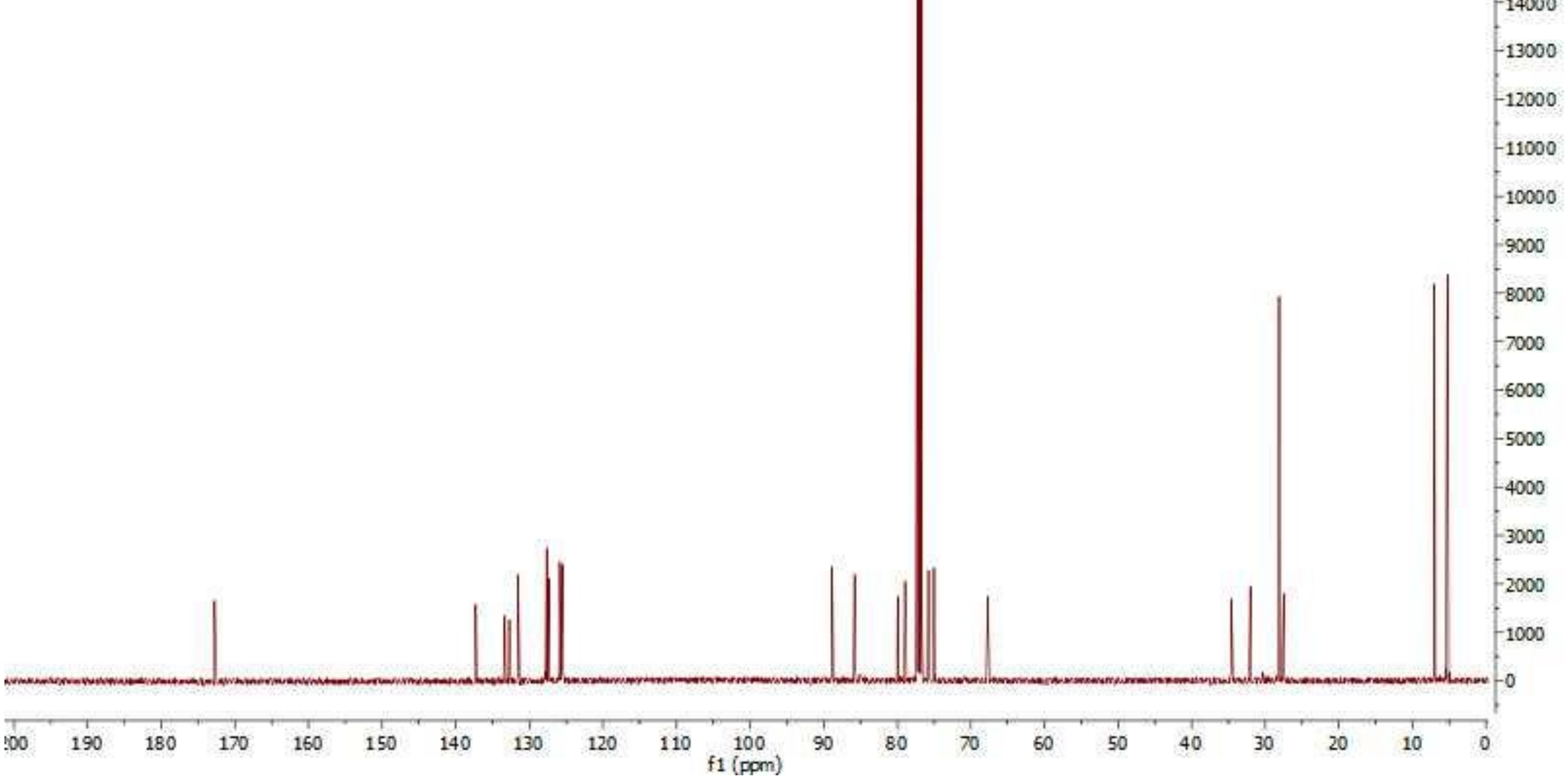

^1H NMR (500 MHz, $CDCl_3$)

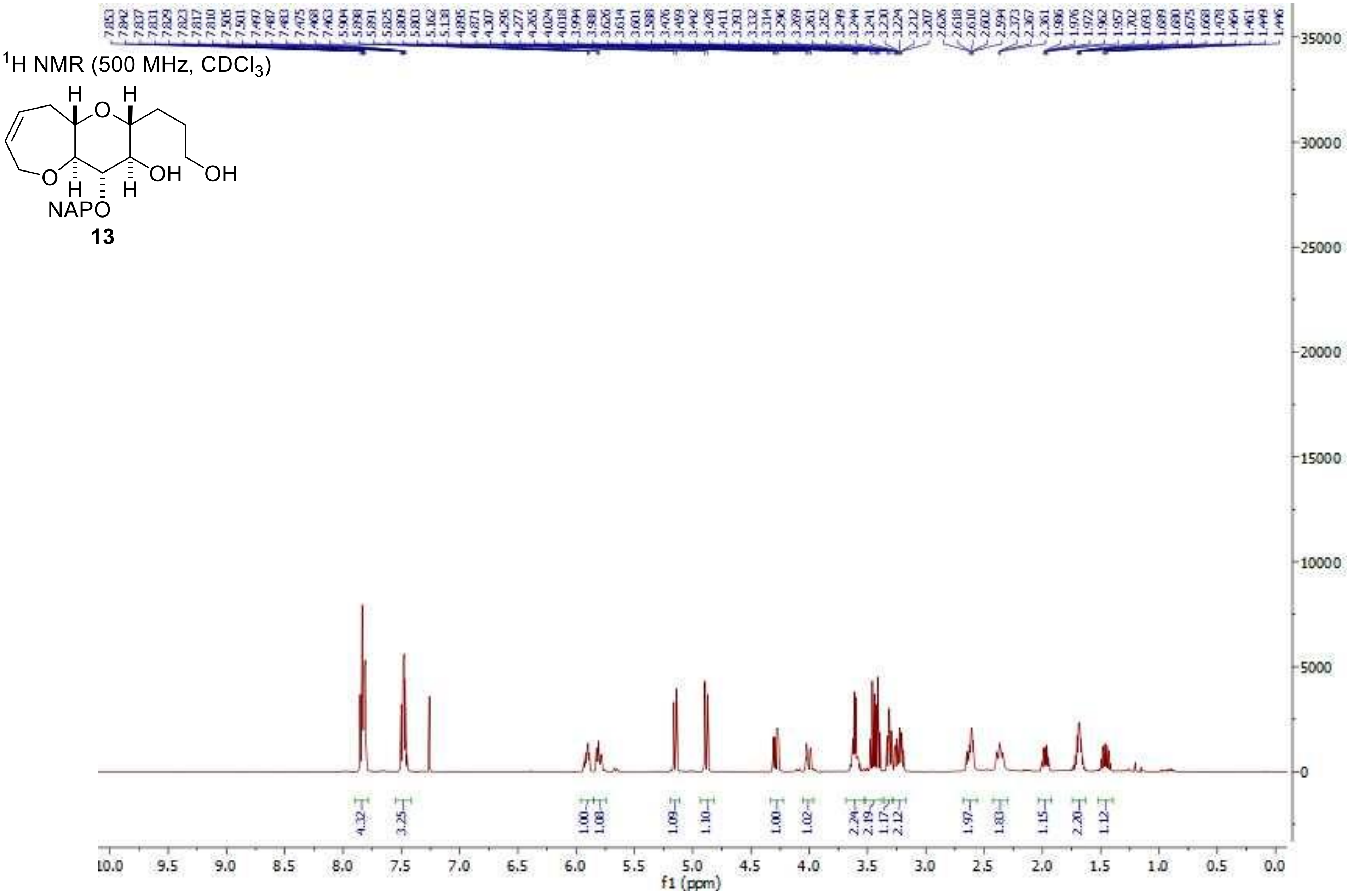

13

^{13}C NMR (126 MHz, $CDCl_3$)

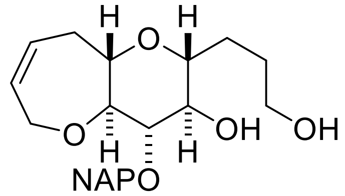

13

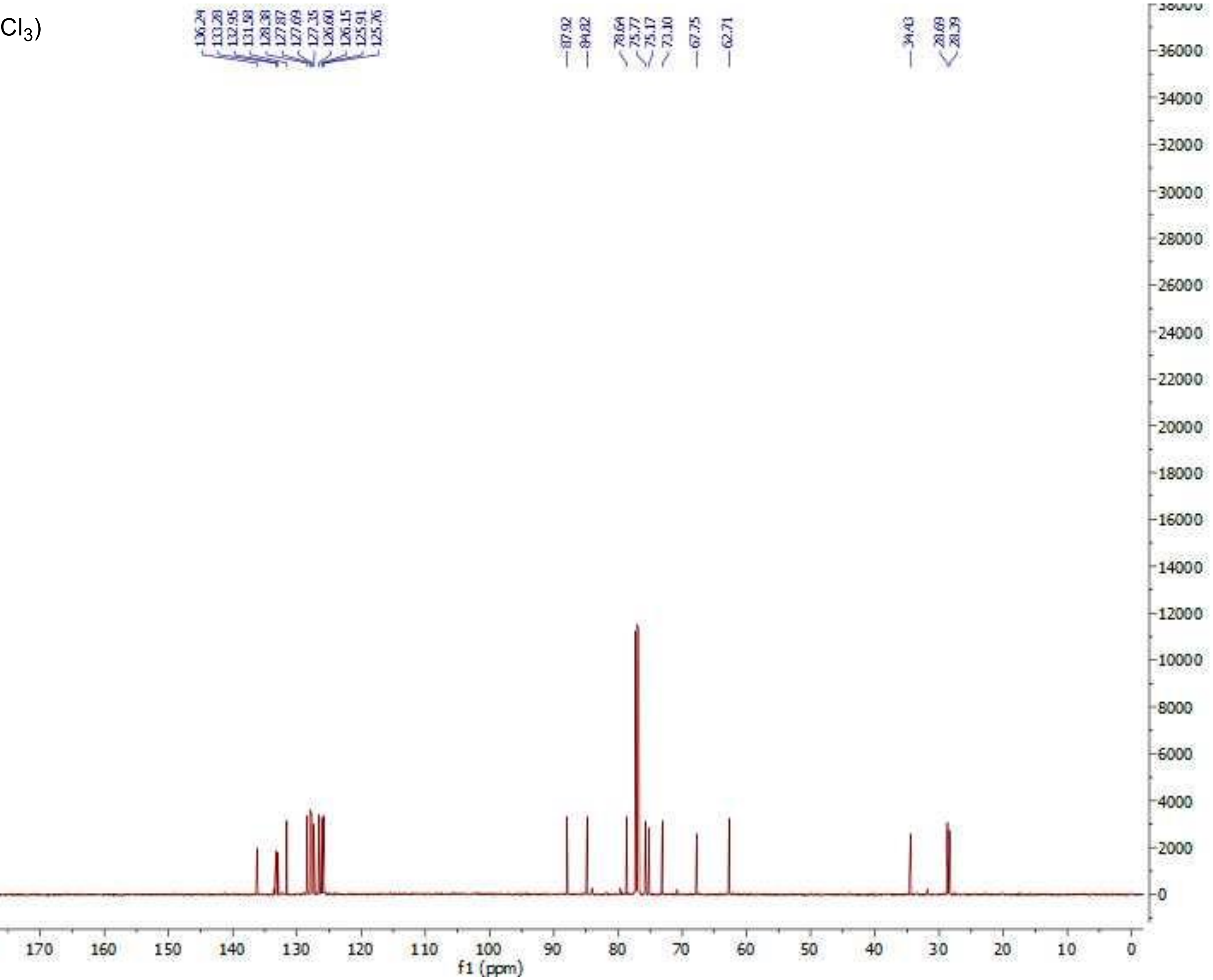

^1H NMR (400 MHz, $CDCl_3$)

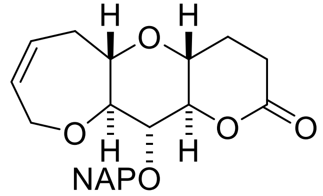

14

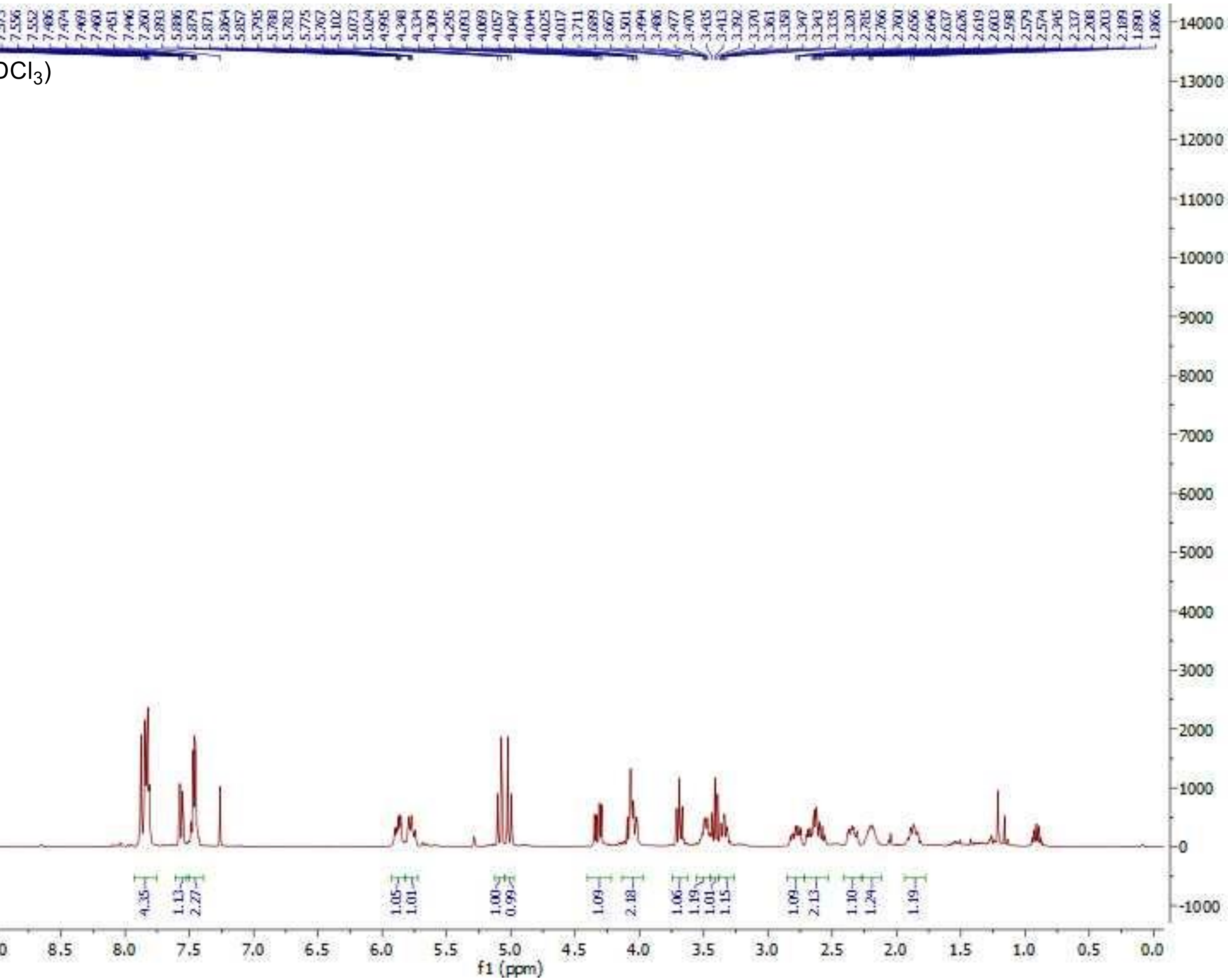

^{13}C NMR (101 MHz, $CDCl_3$)

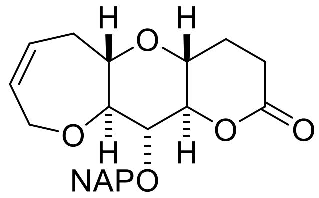

14

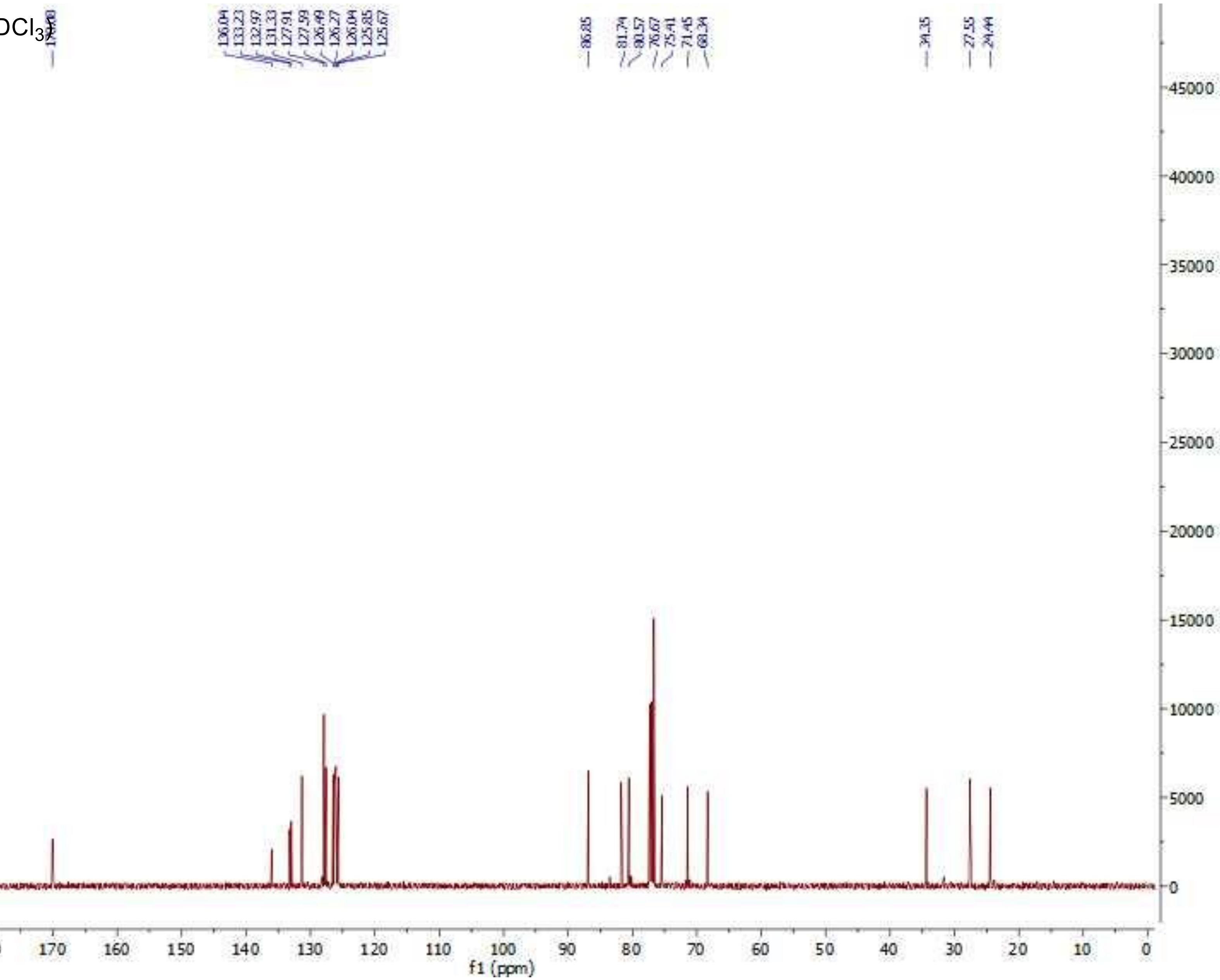

^{1}H NMR (500 MHz, $CDCl_3$)

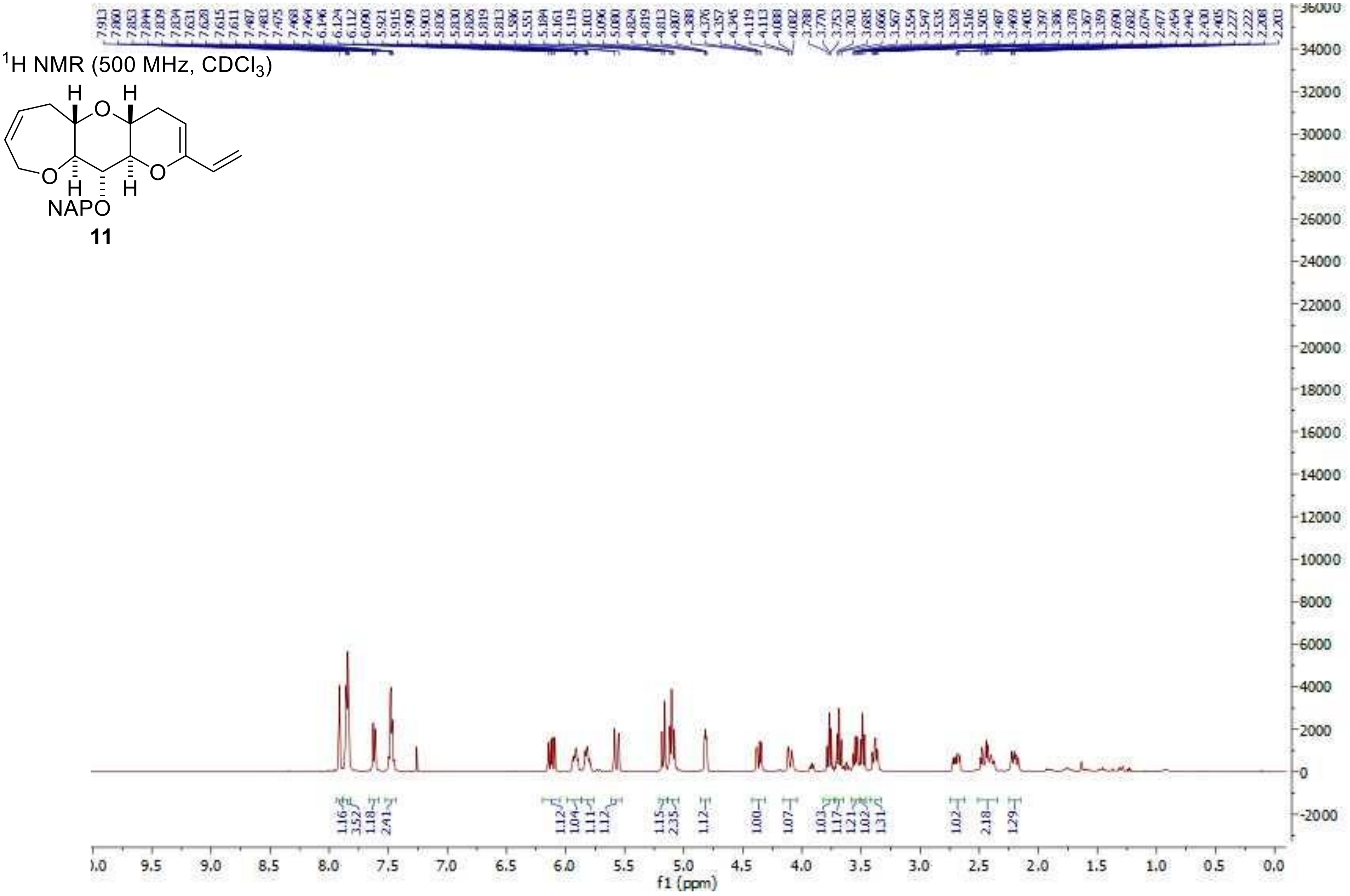

11

^{13}C NMR (126 MHz, $CDCl_3$)

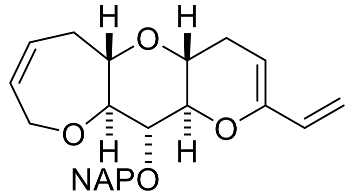

11

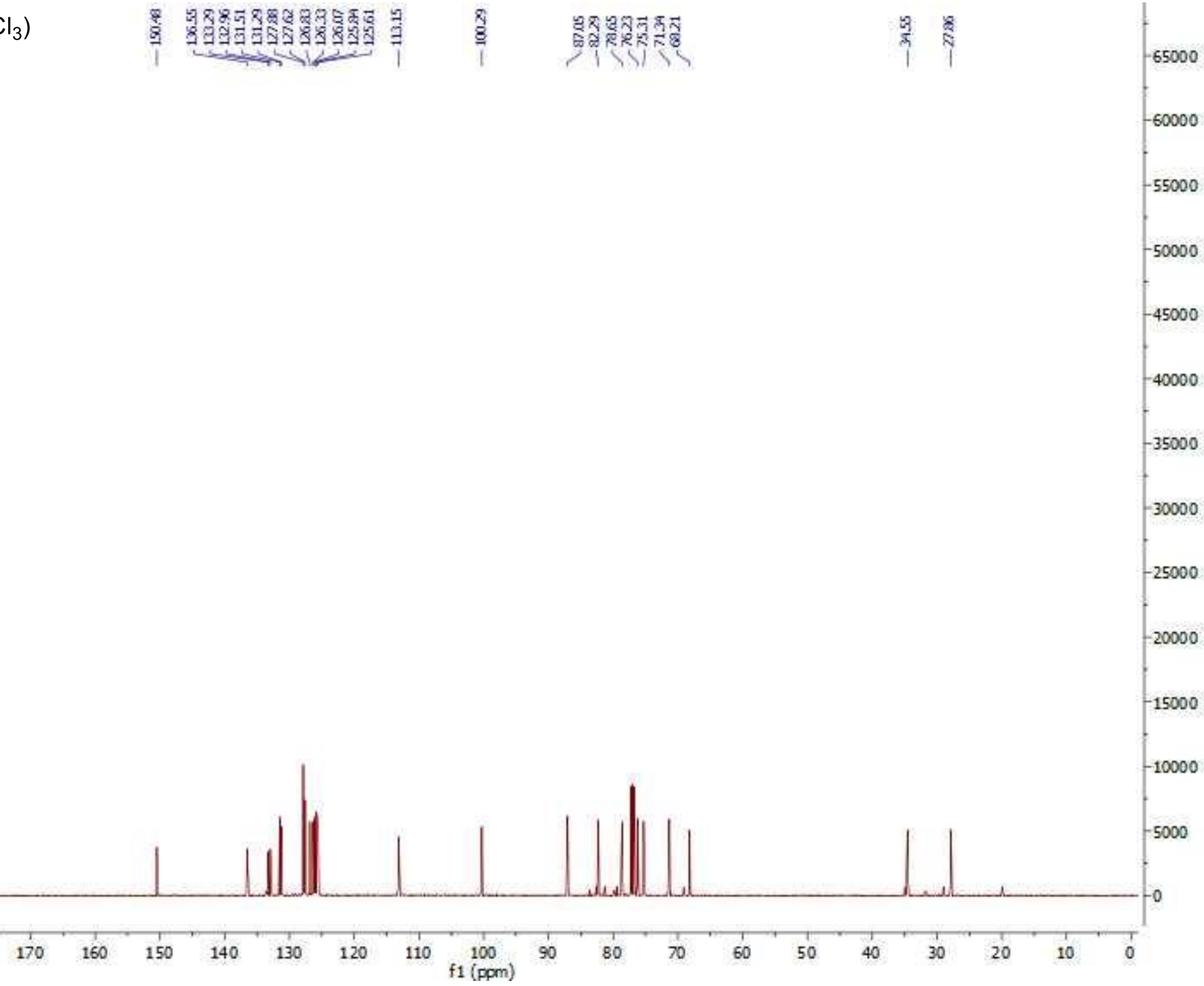

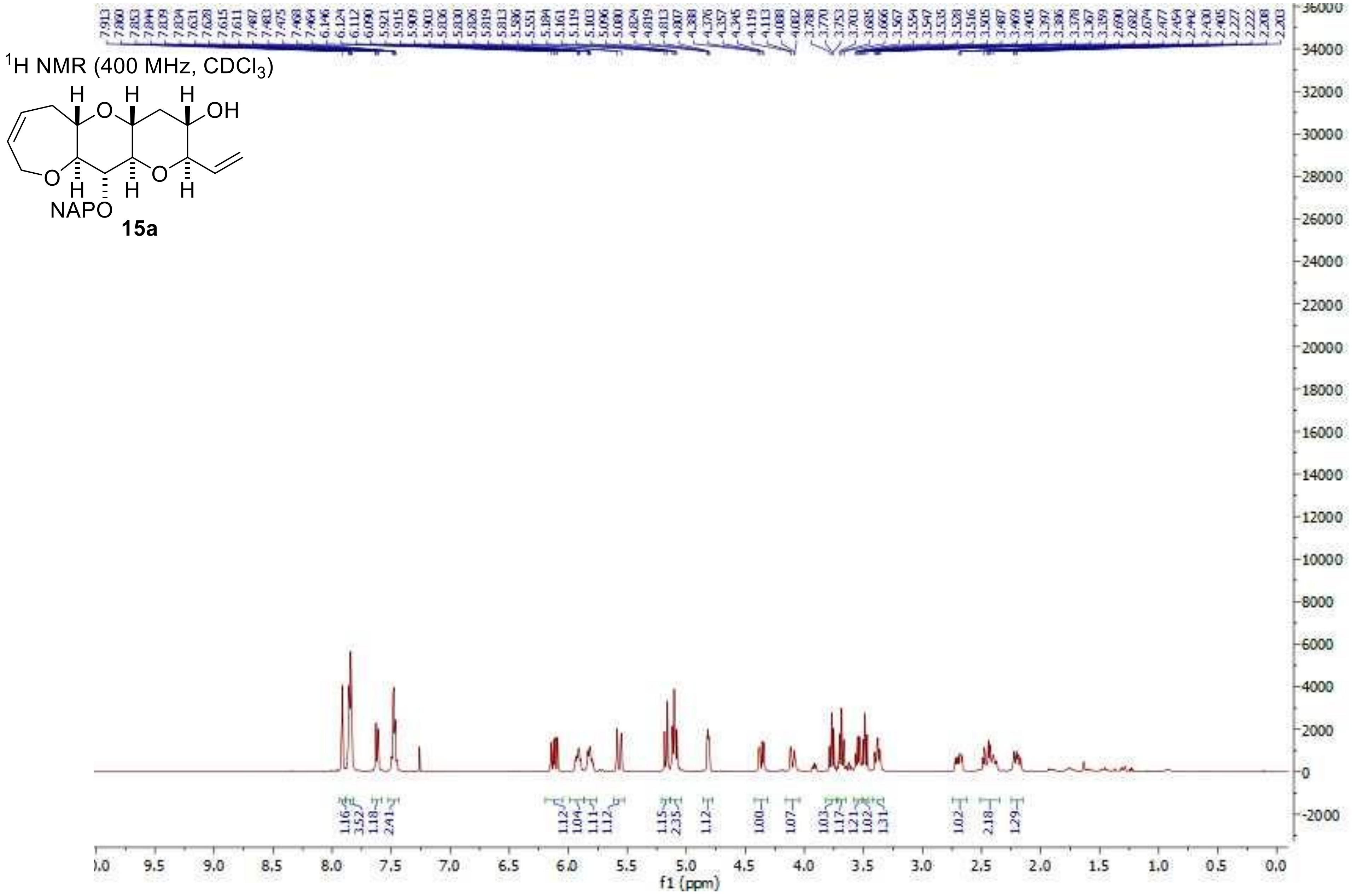

¹H NMR (400 MHz, CDCl3)
OH
NAPO
15a
f1 (ppm)

^{13}C NMR (101 MHz, $CDCl_3$)

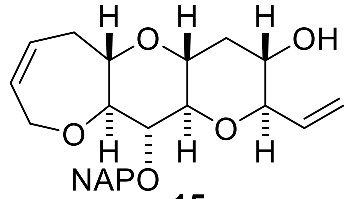

15a

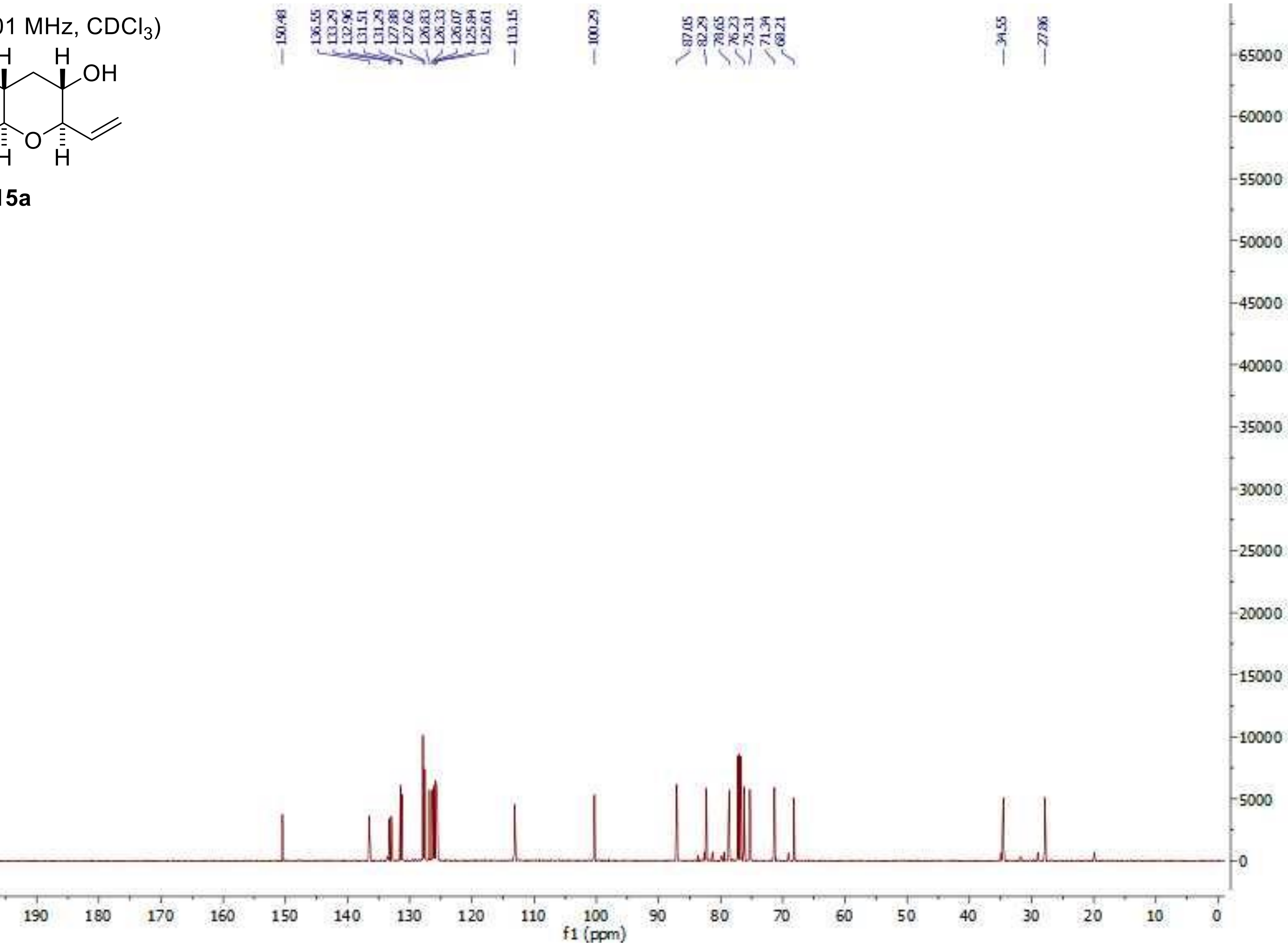

^{1}H NMR (400 MHz, $CDCl_3$)

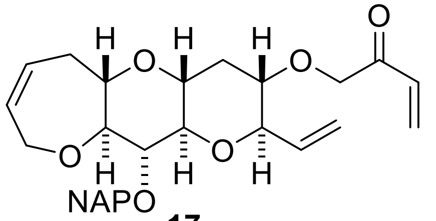

17

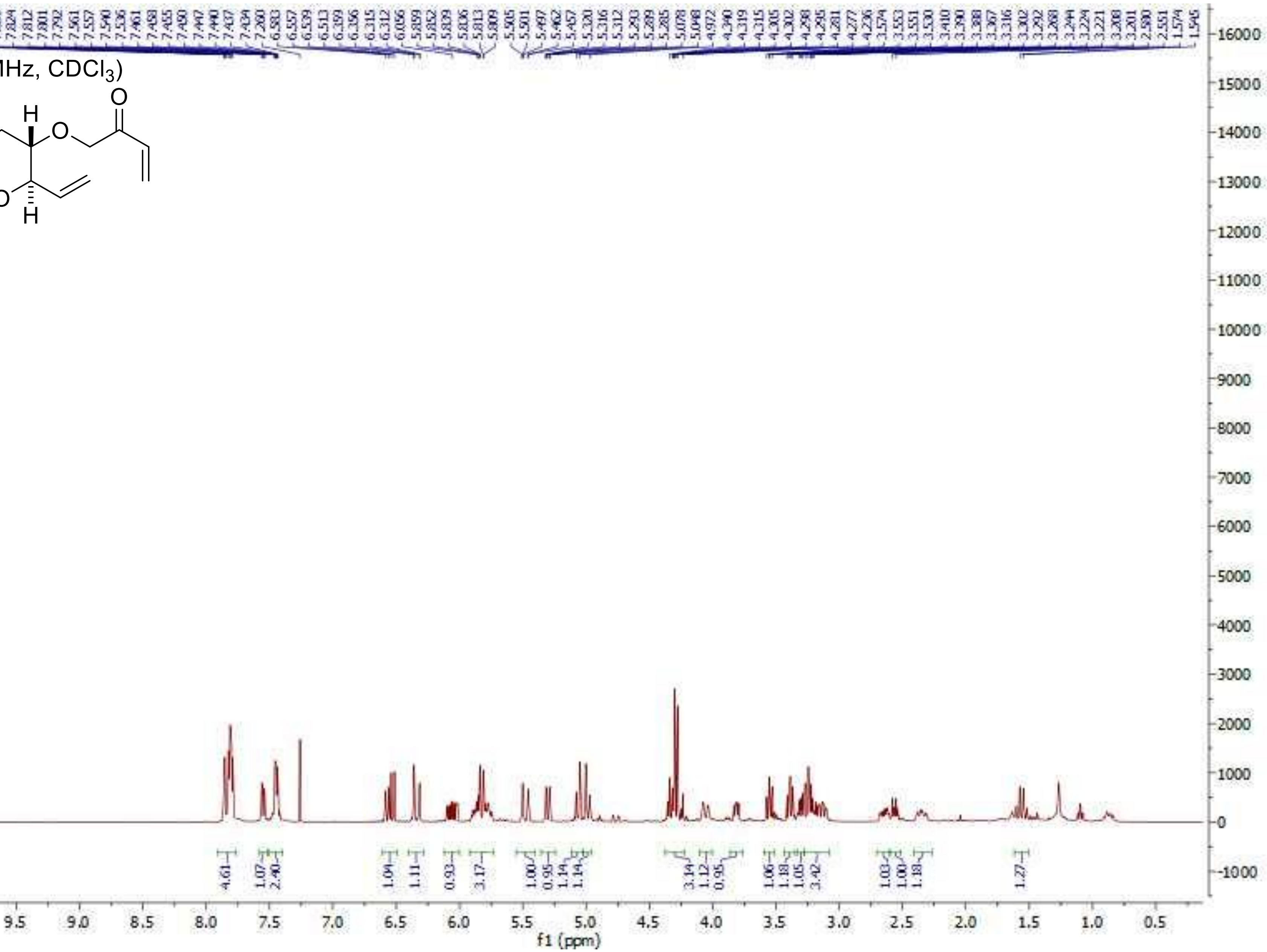

^{13}C NMR (101 MHz, $CDCl_3$)

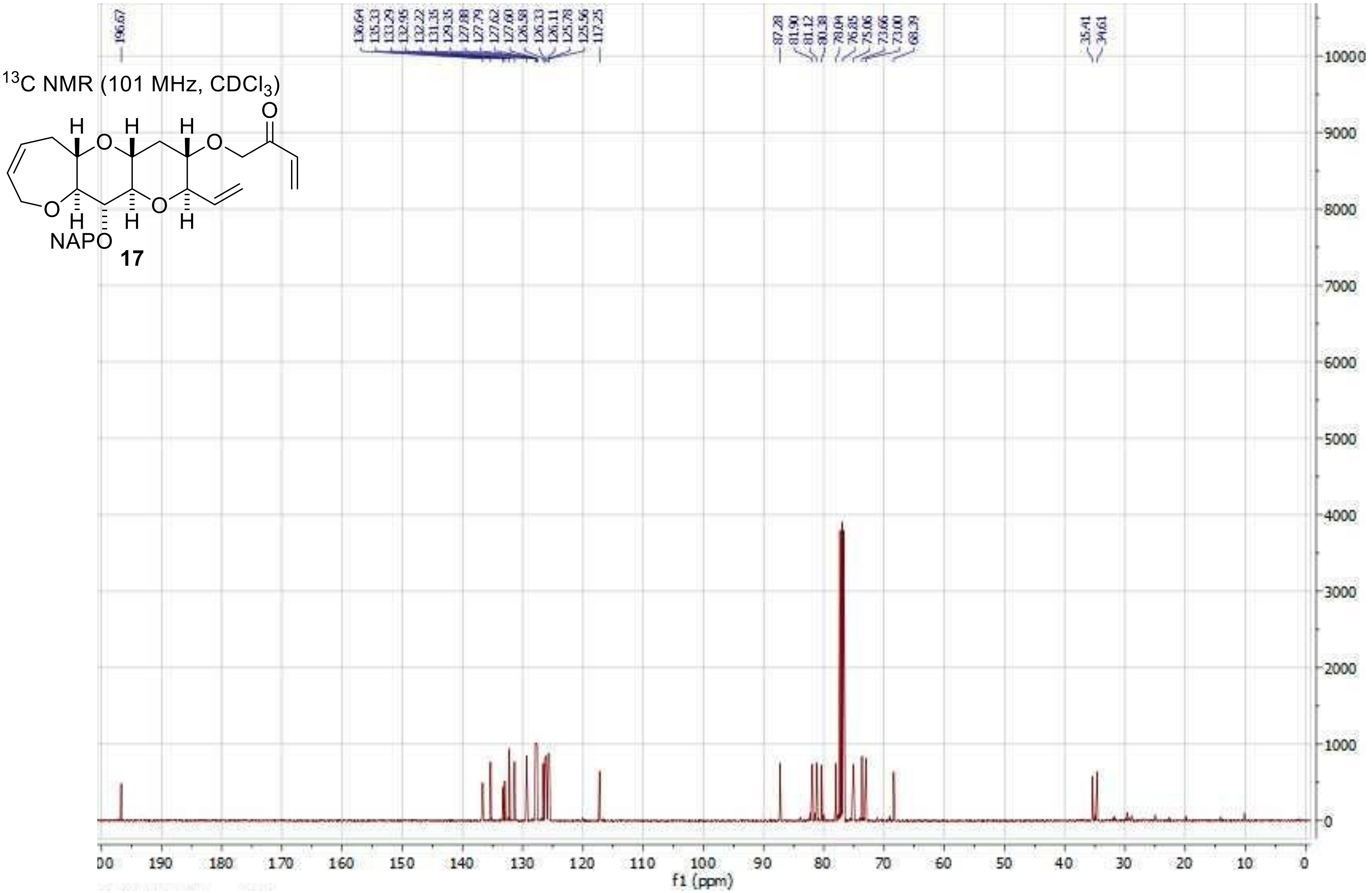

^{1}H NMR (400 MHz, $CDCl_3$)

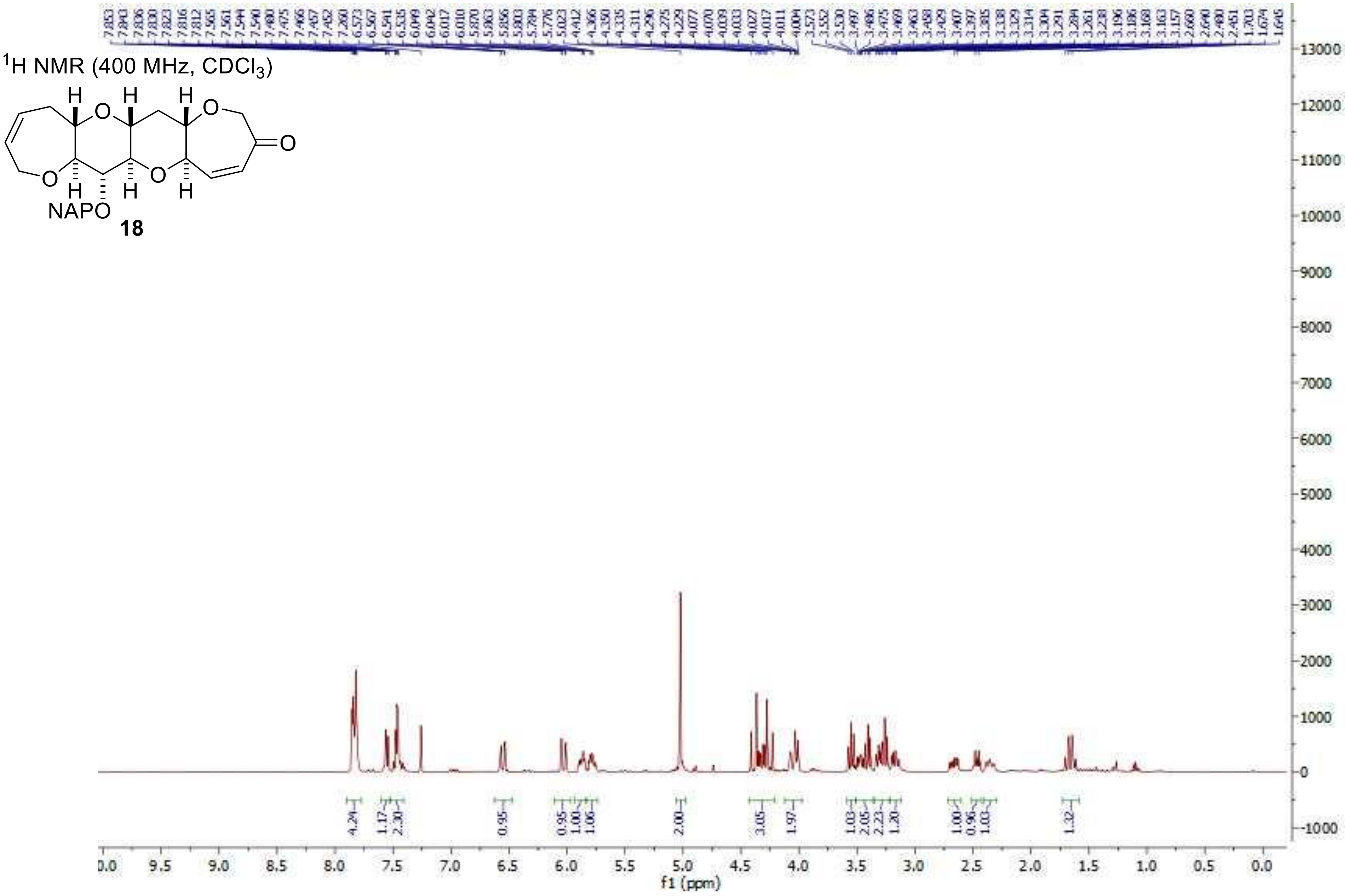

18

^{13}C NMR (101 MHz, $CDCl_3$)

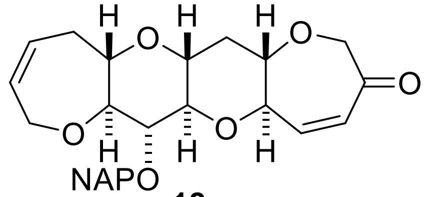

18

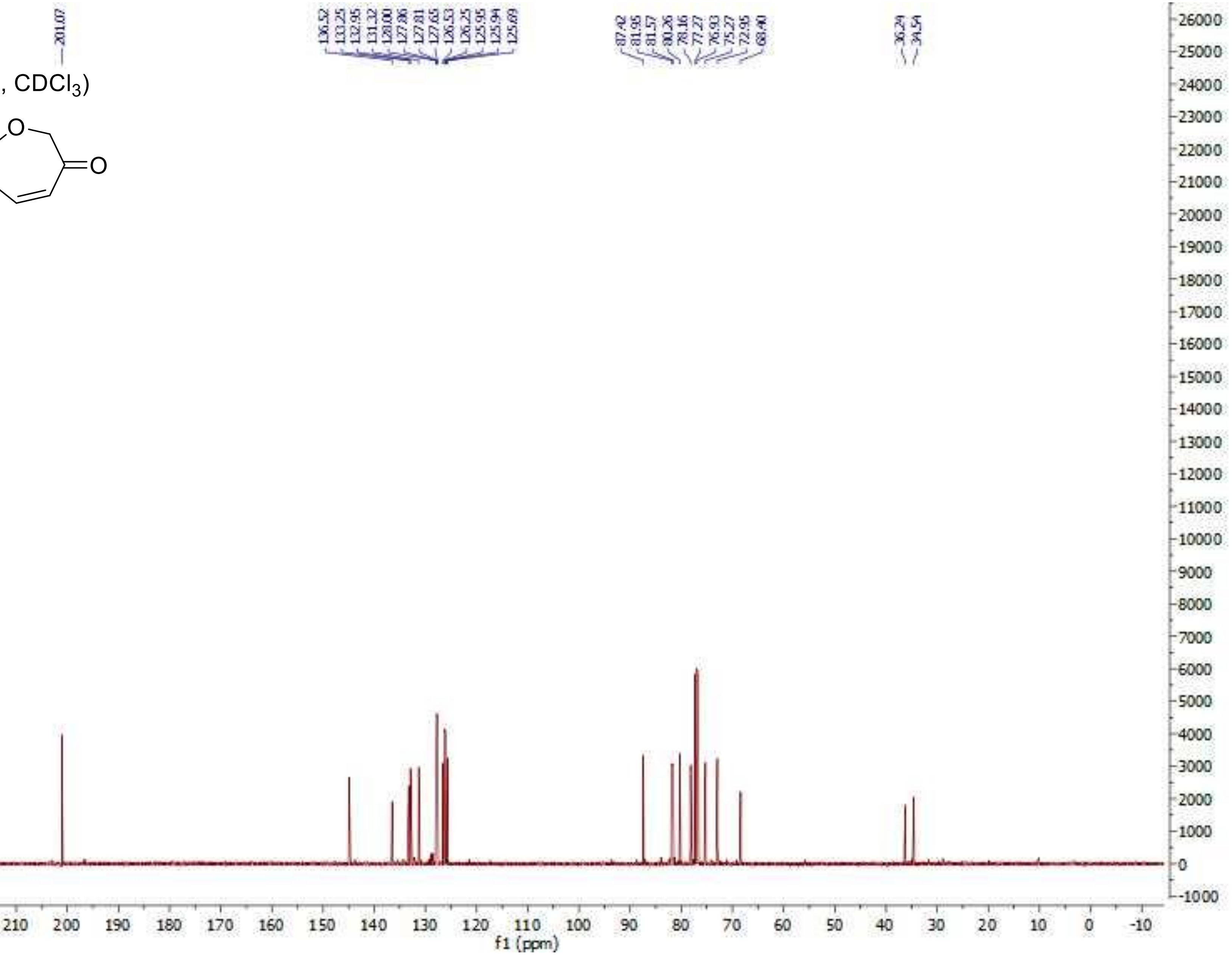

^{1}H NMR (500 MHz, $CDCl_3$)

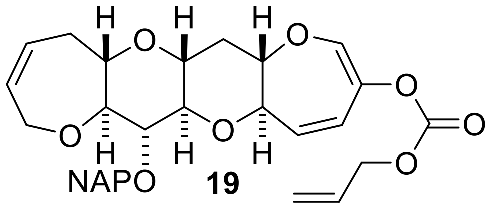

19

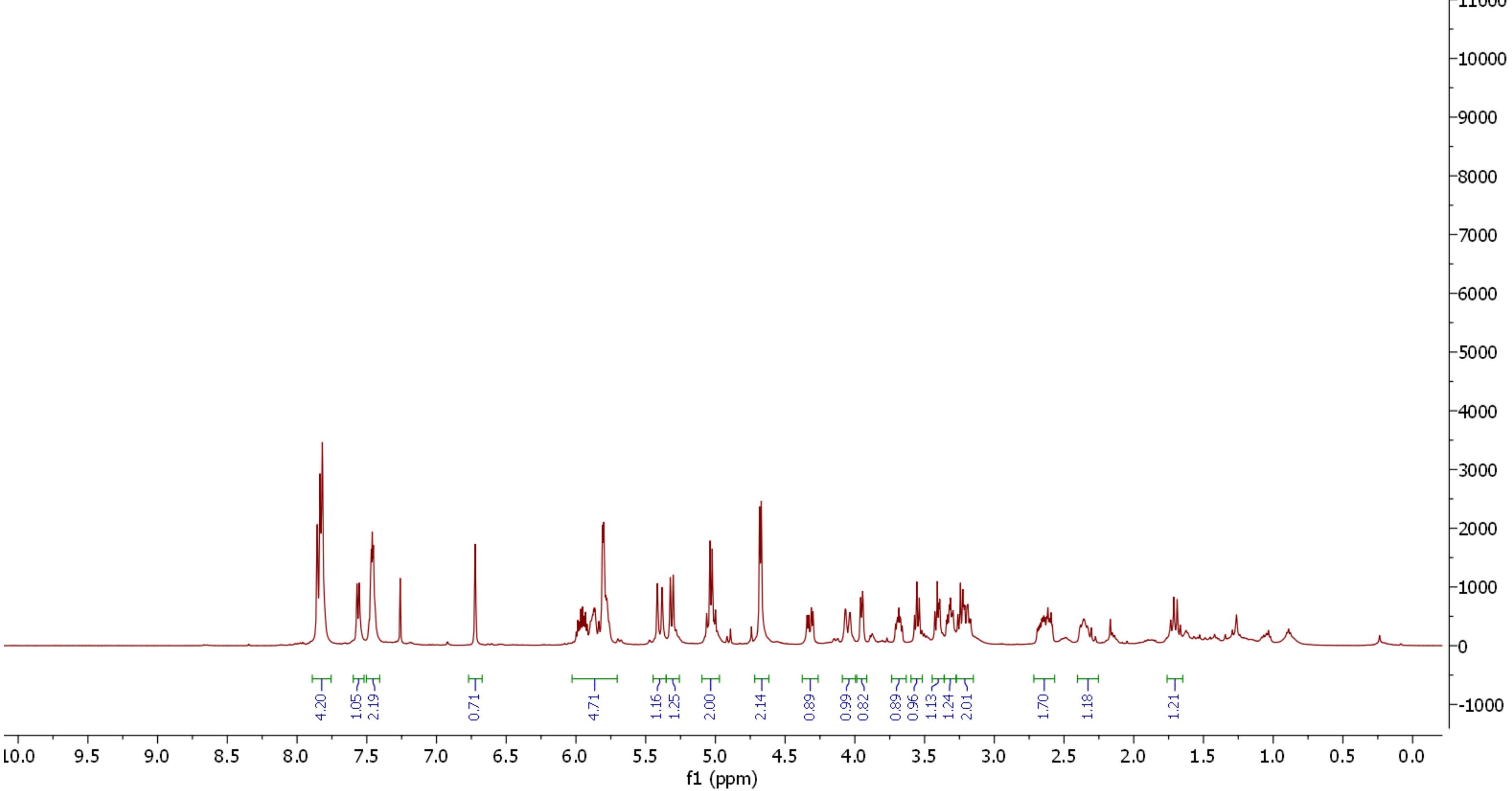

MT-VI-768.11.fid
user Myron Triantafyllakis
AD allyl carbonate
C13CPD1024.GLA CDCl3 /u myron 25

^{13}C NMR (126 MHz, $CDCl_3$)

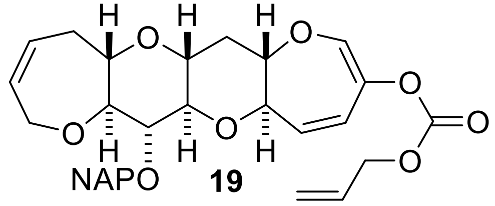

19

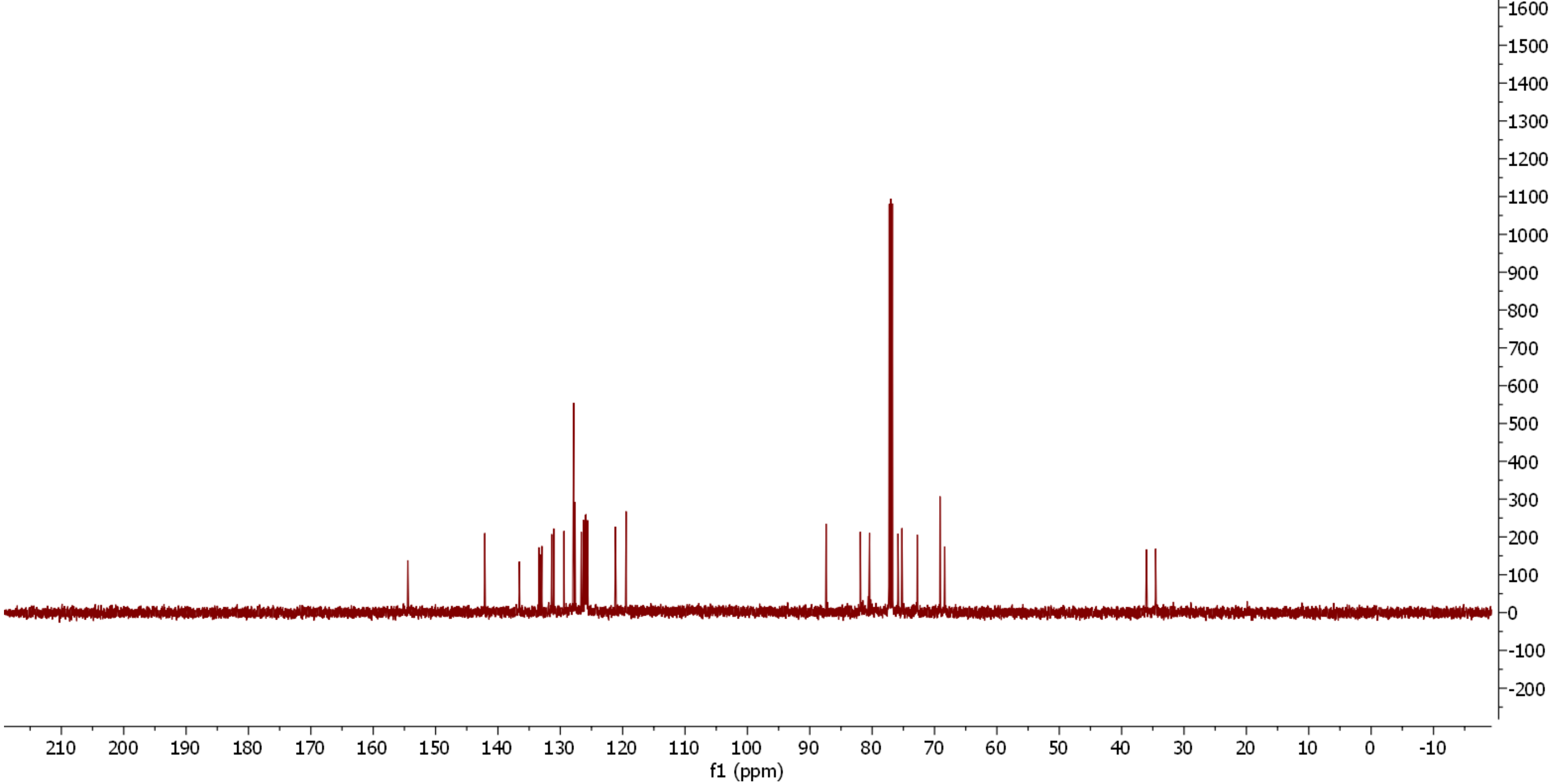

^{1}H NMR (400 MHz, $CDCl_3$)

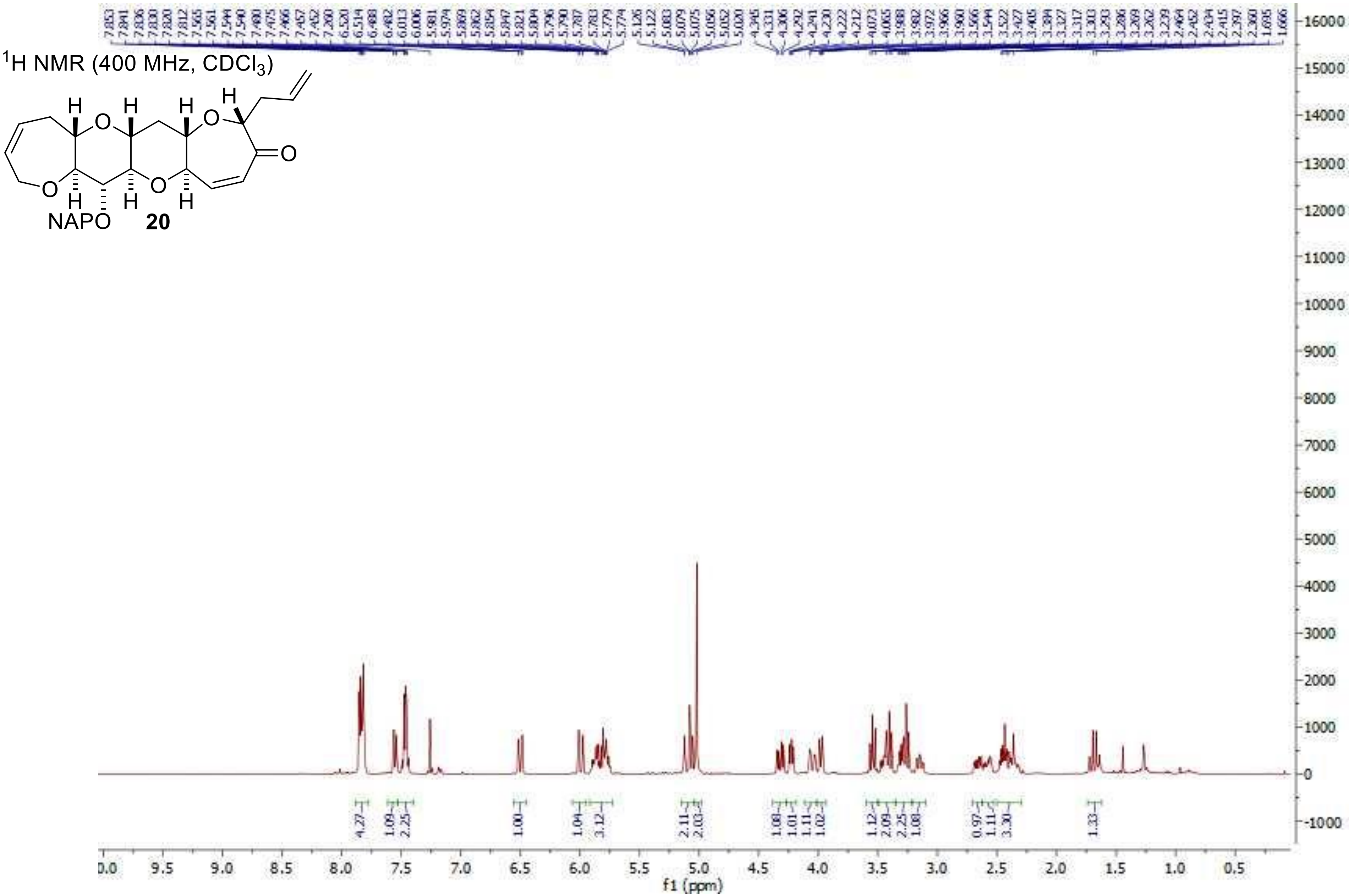

^{13}C NMR (101 MHz, $CDCl_3$)

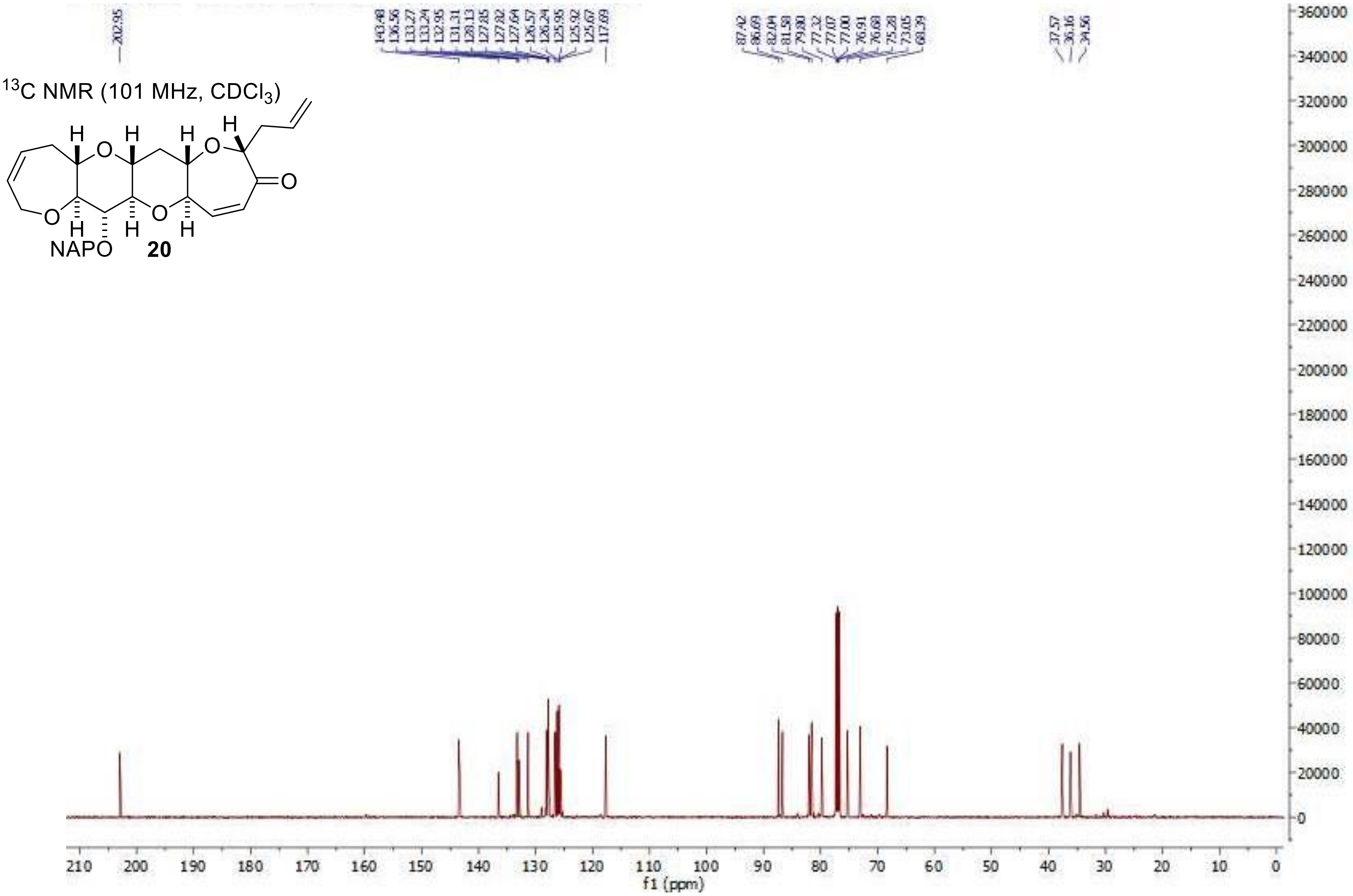

^{1}H NMR (500 MHz, $CDCl_3$)

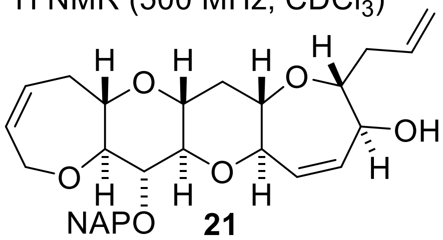

21

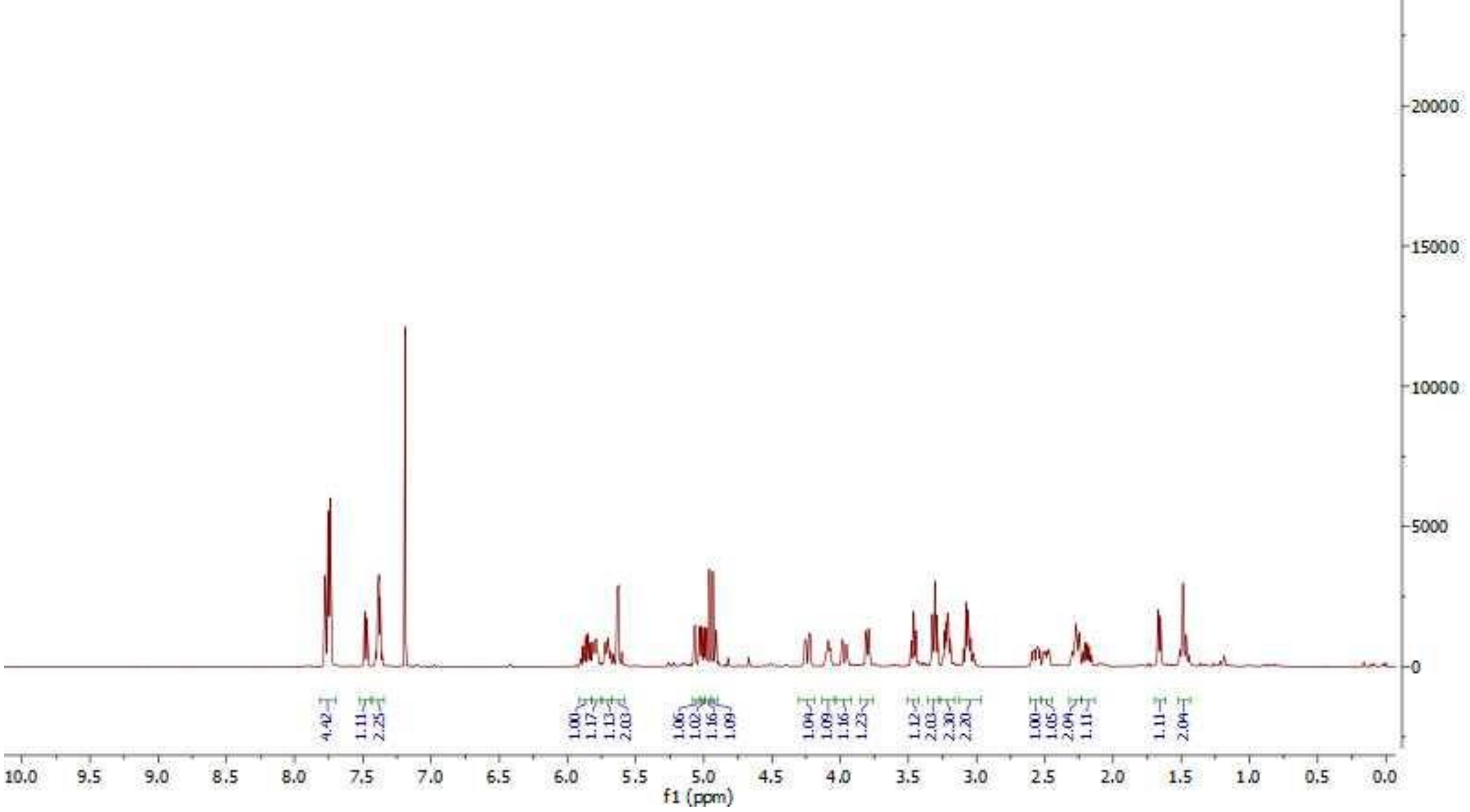

^{13}C NMR (126 MHz, $CDCl_3$)

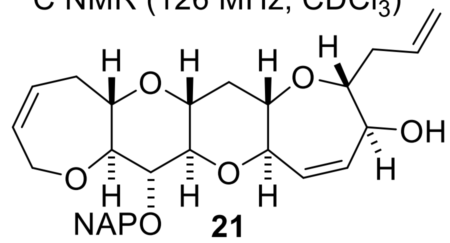

21

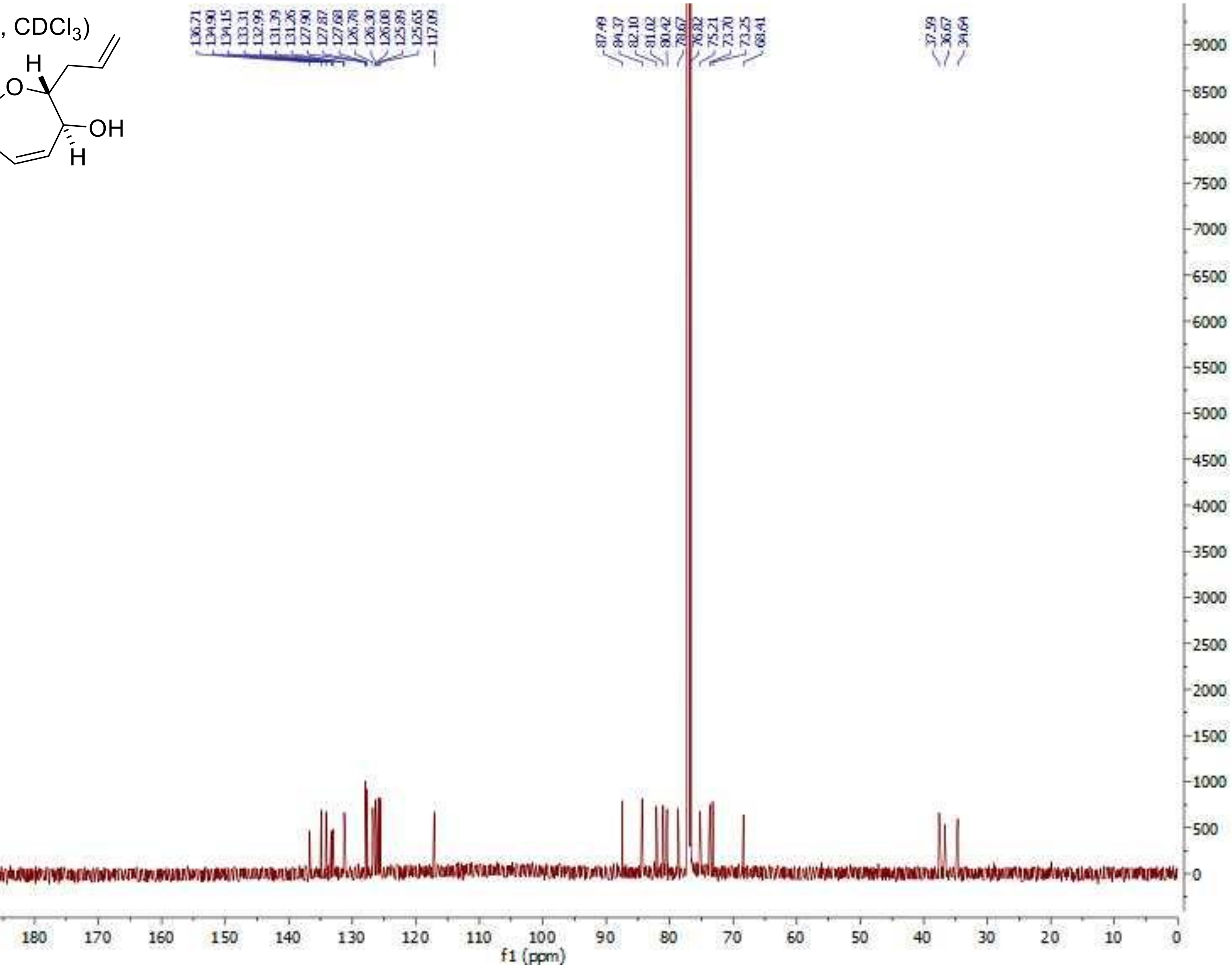

^{1}H NMR (400 MHz, $CDCl_3$)

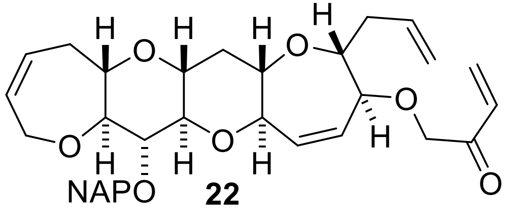

22

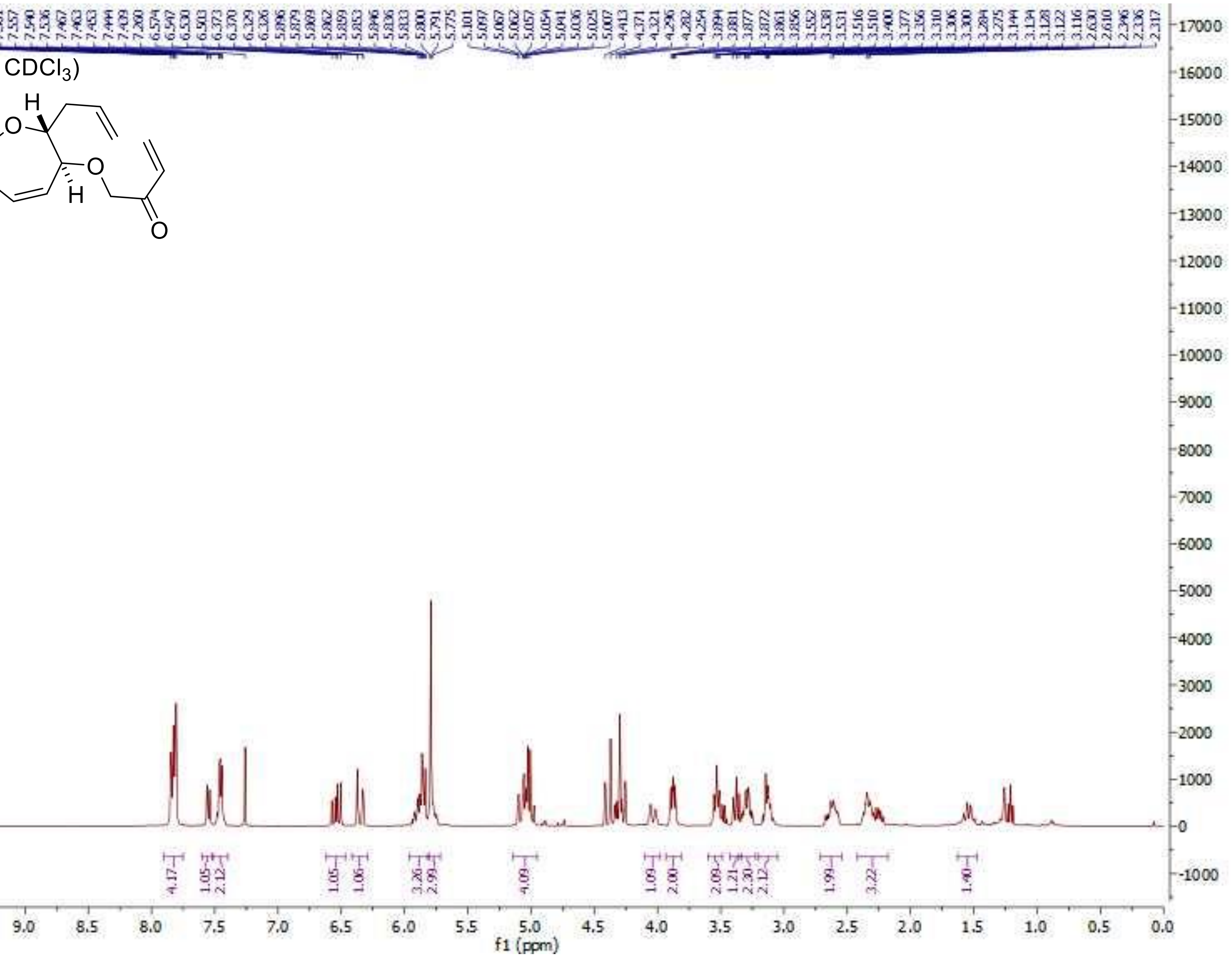

^{13}C NMR (101 MHz, $CDCl_3$)

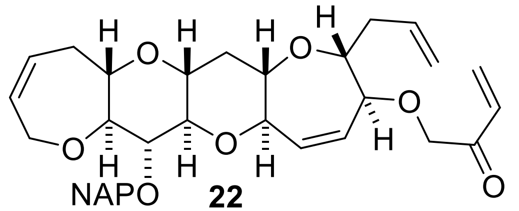

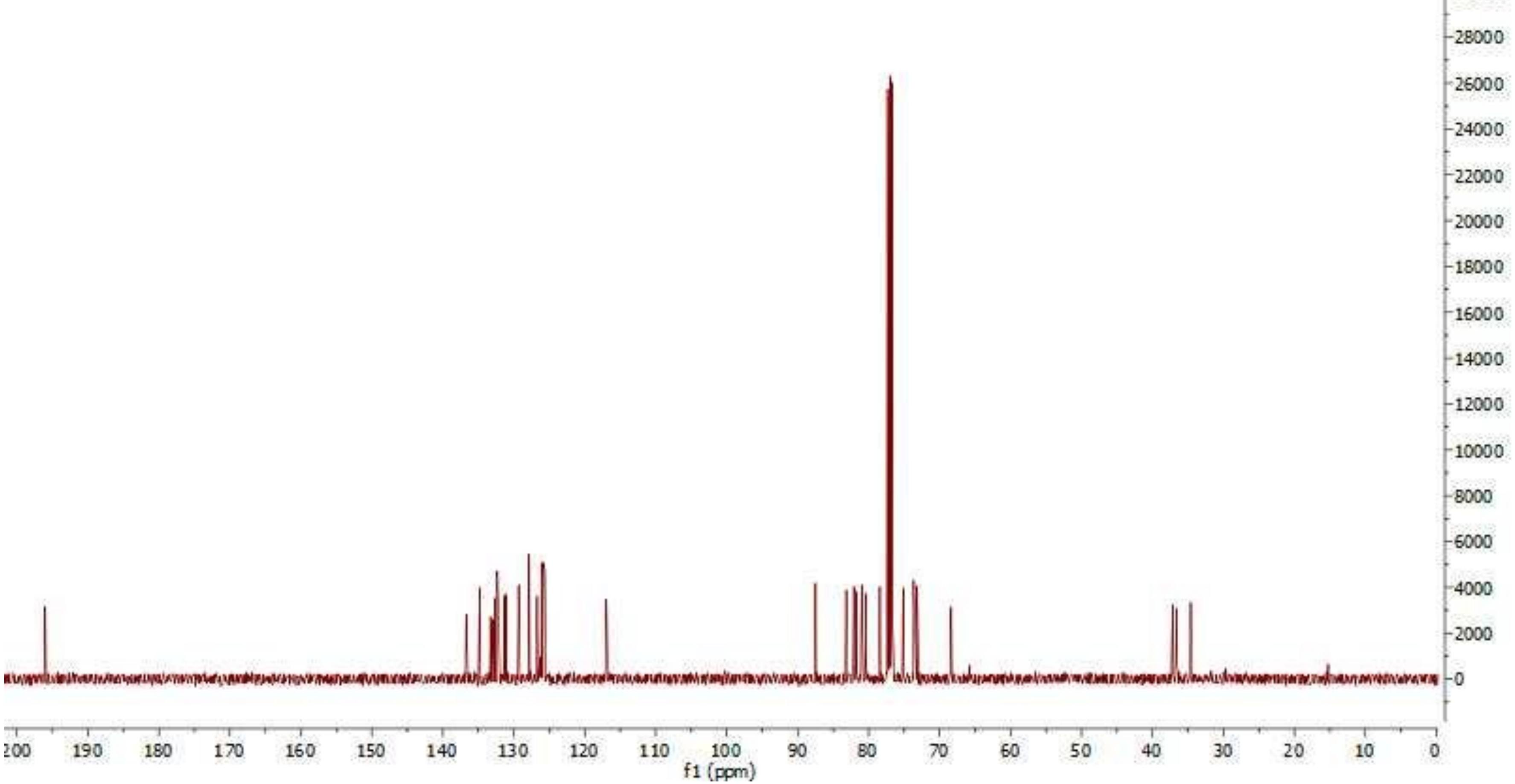

^{1}H NMR (400 MHz, $CDCl_3$)

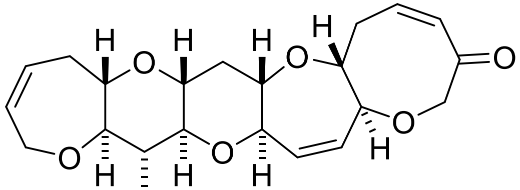

23

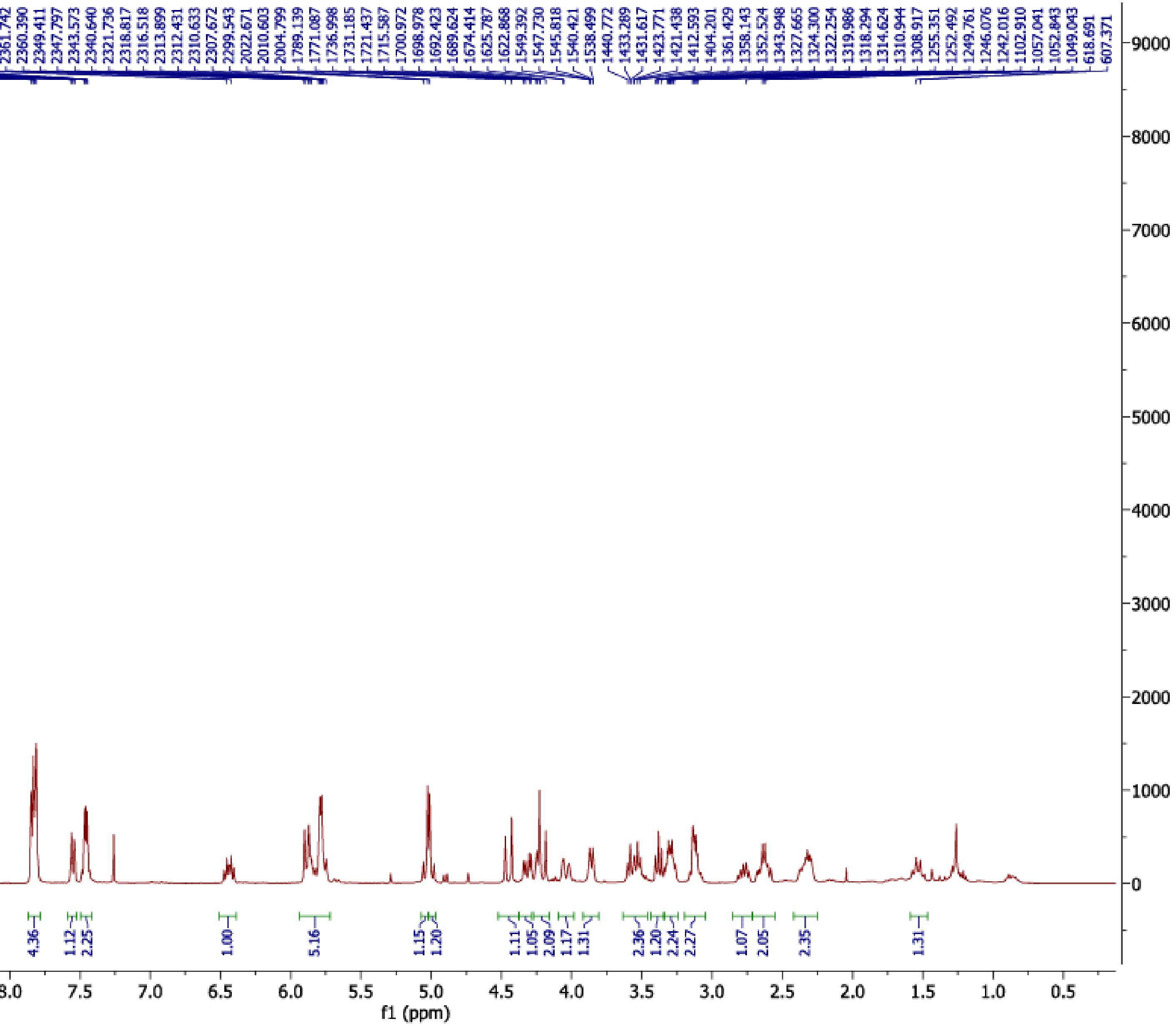

^{13}C NMR (101 MHz, $CDCl_3$)

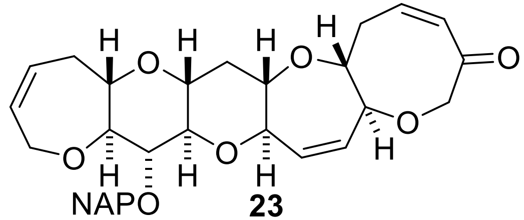

23

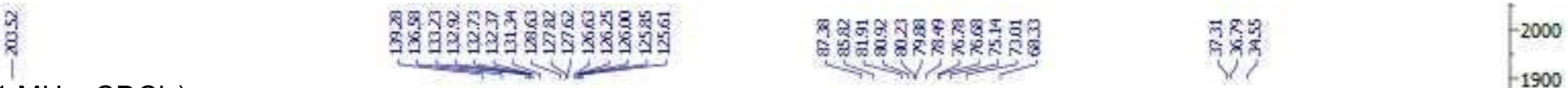

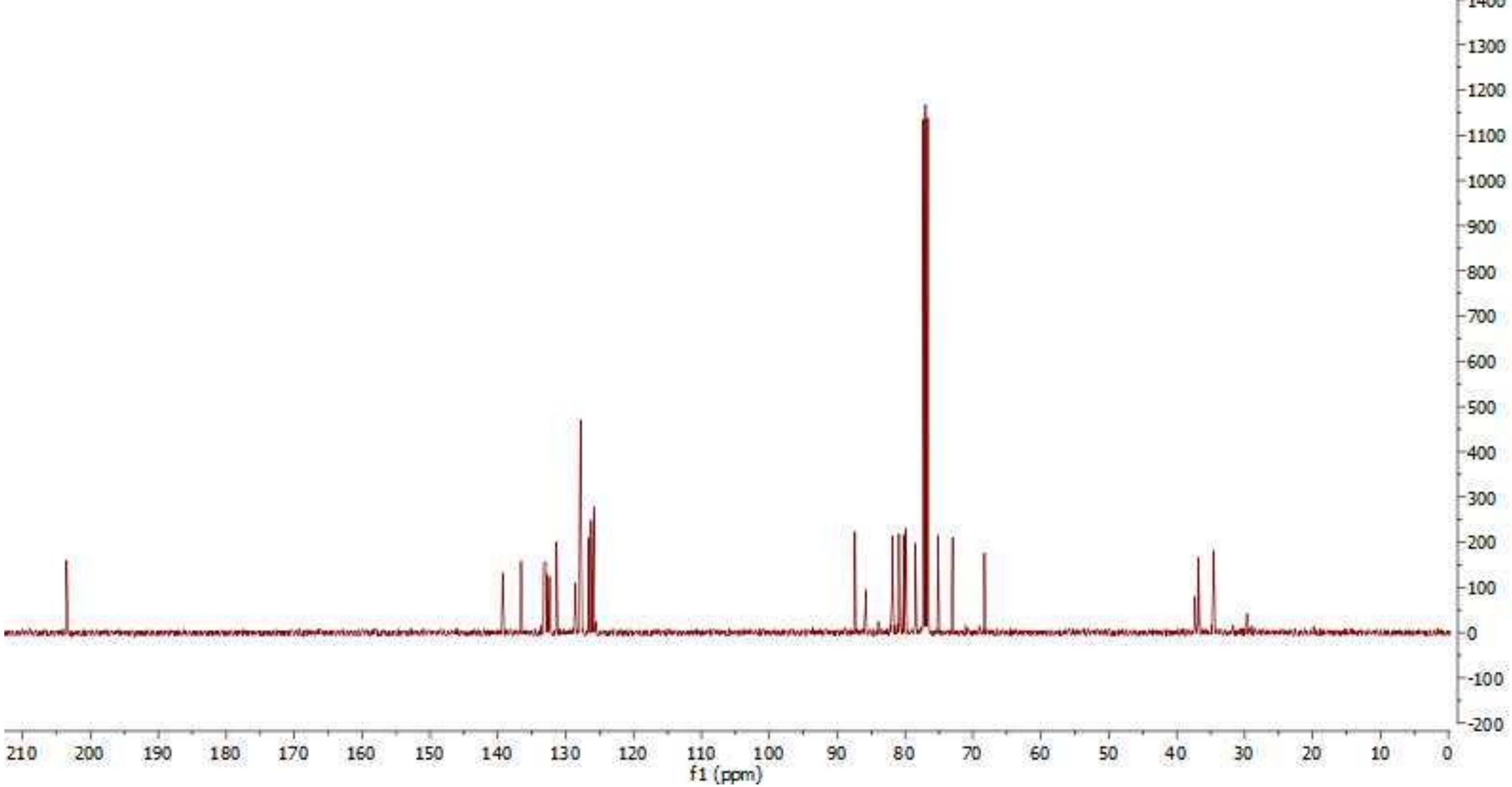

^{1}H NMR (500 MHz, $CDCl_3$)

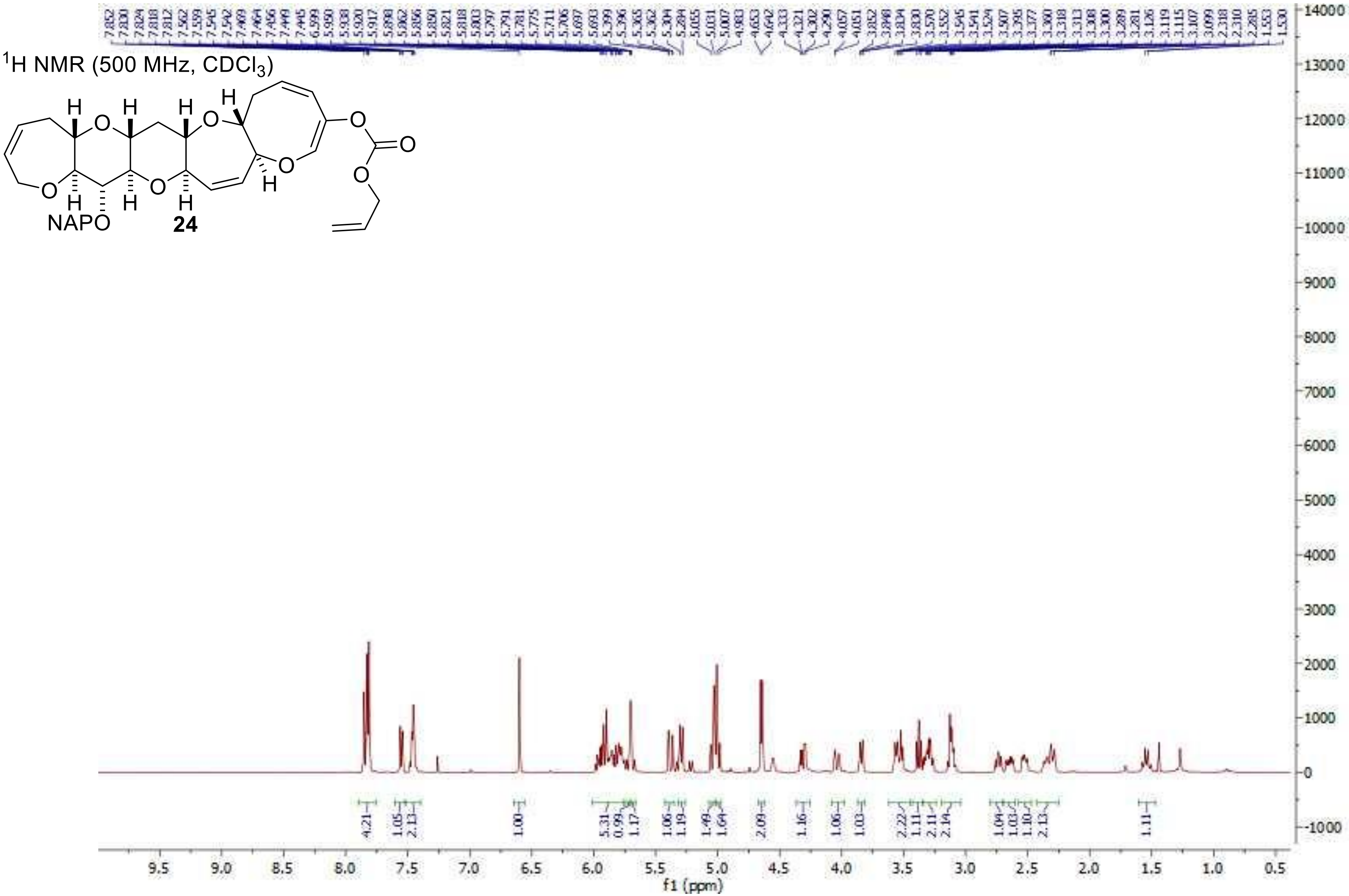

^{13}C NMR (126 MHz, $CDCl_3$)

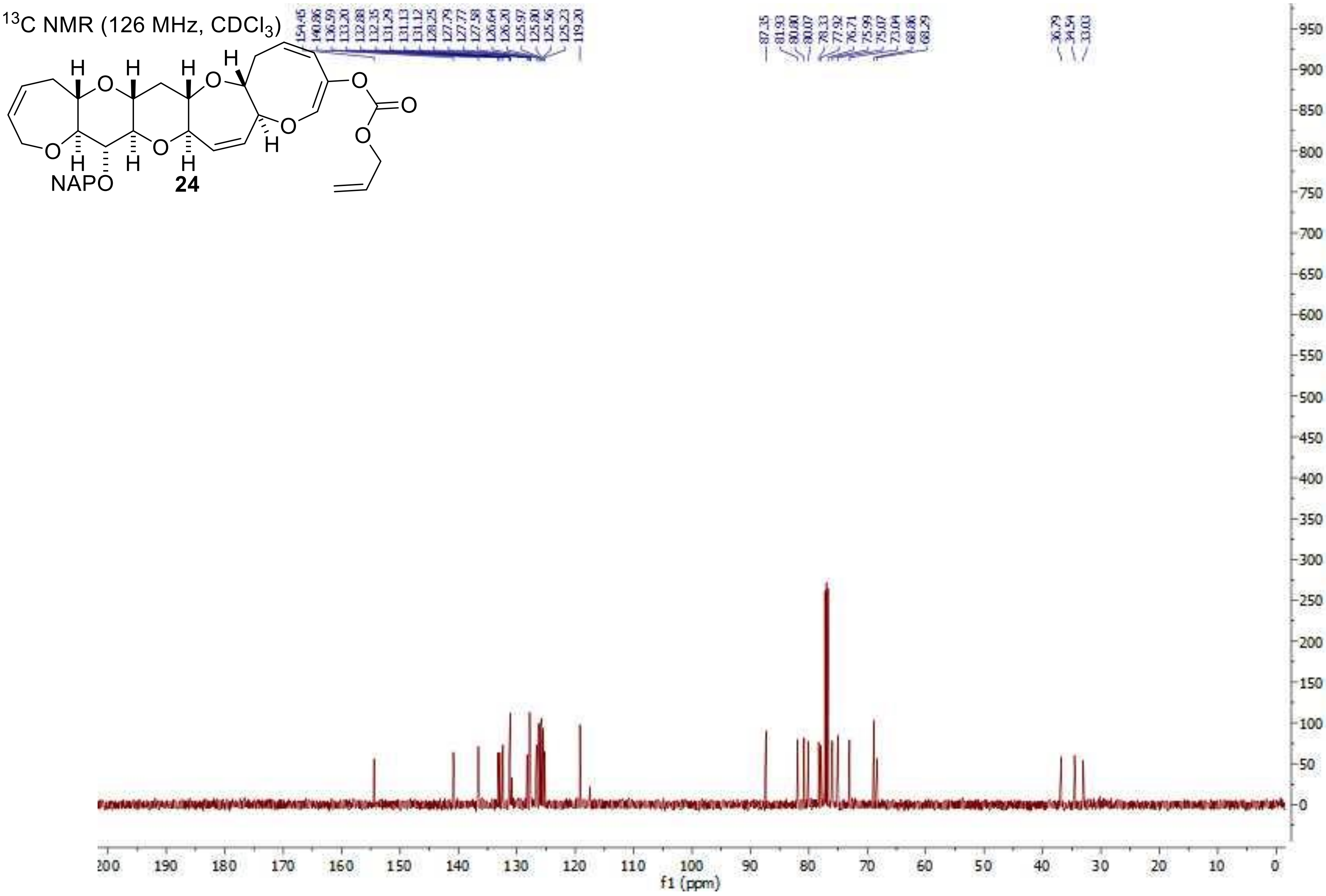

^{1}H NMR (400 MHz, $CDCl_3$)

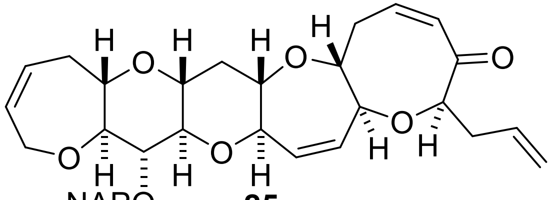

25

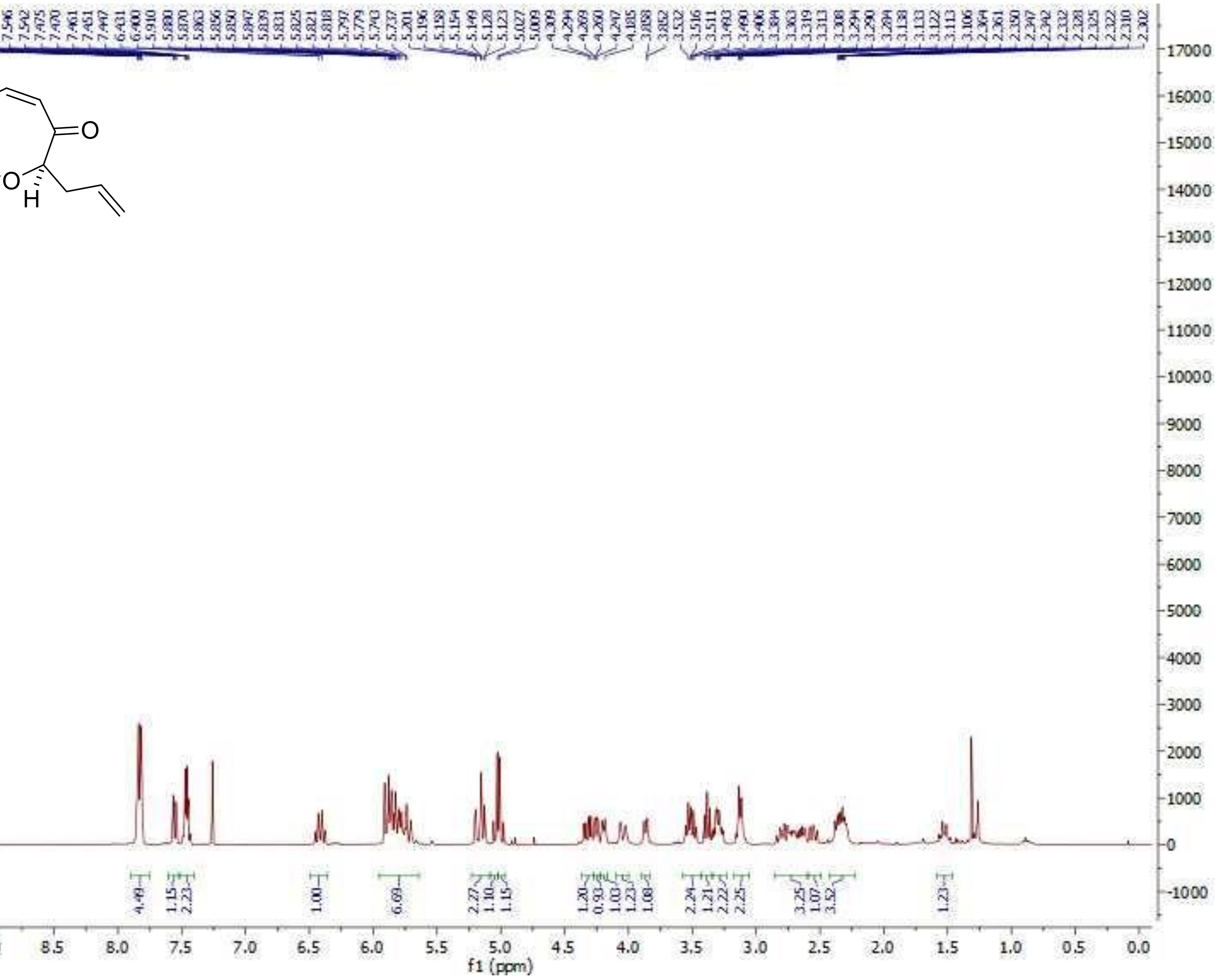

^{13}C NMR (101 MHz, $CDCl_3$)

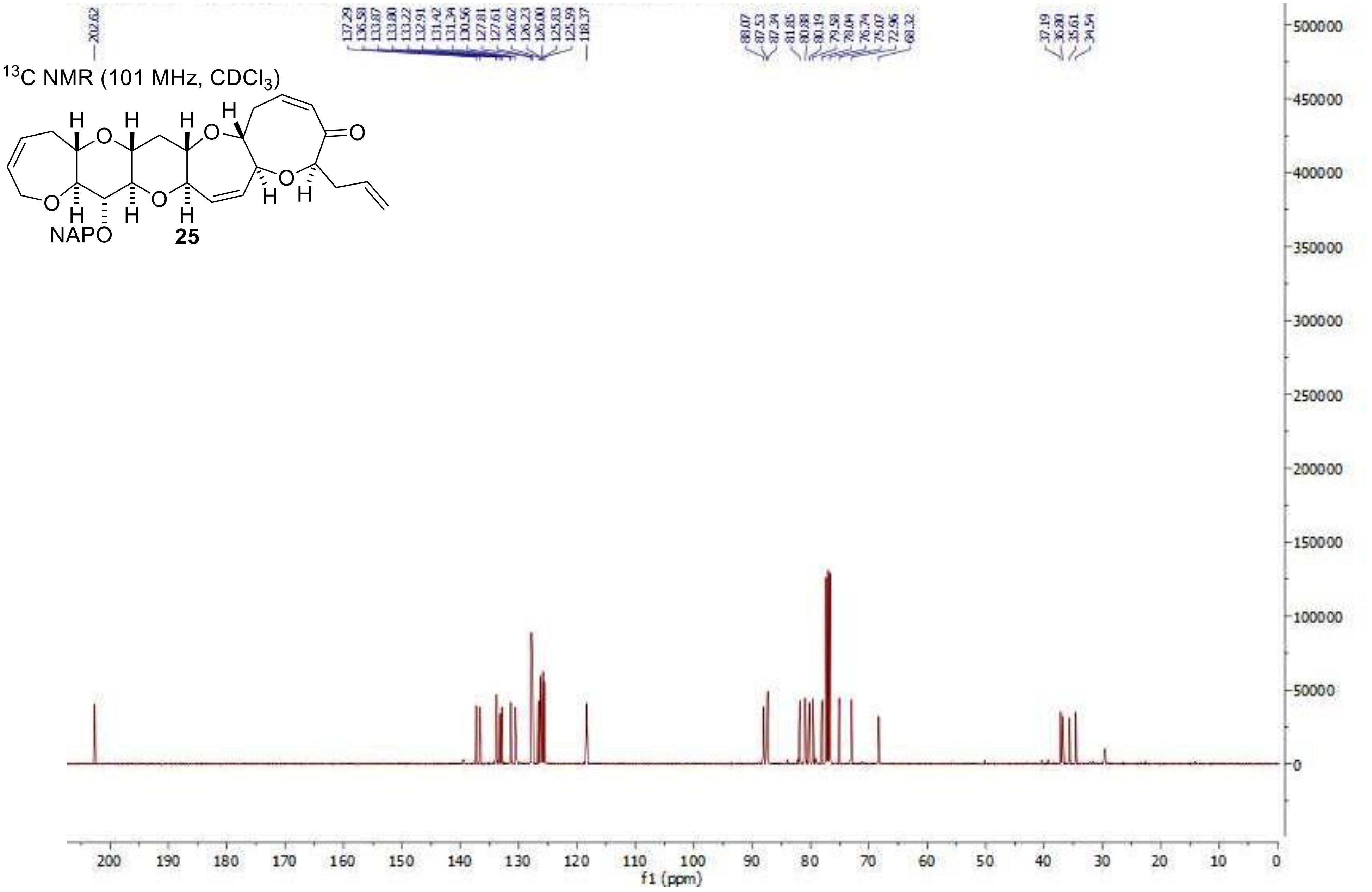

^1H NMR (400 MHz, $CDCl_3$)

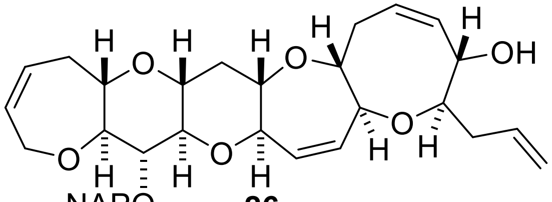

26

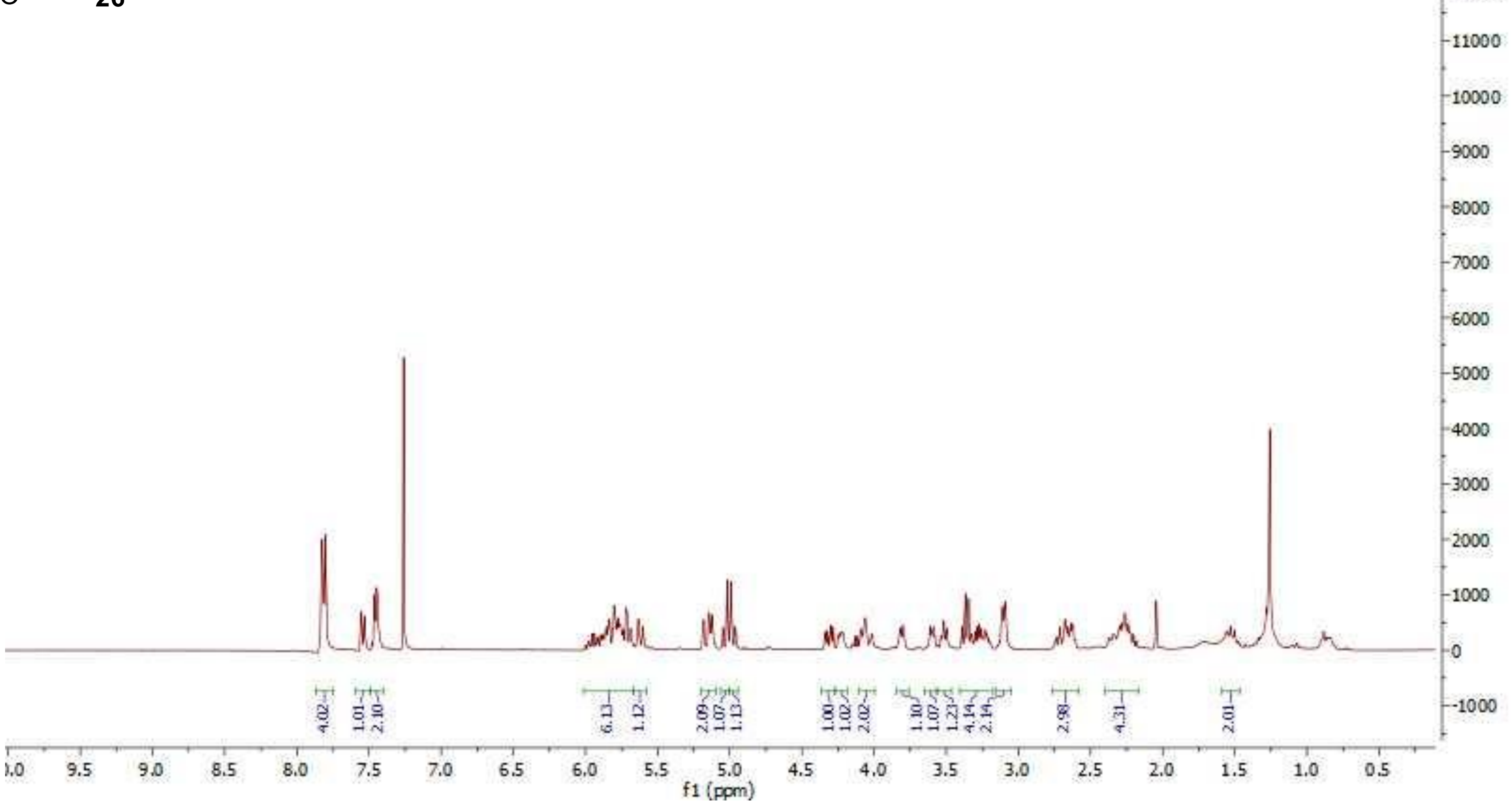

^{13}C NMR (101 MHz, $CDCl_3$)

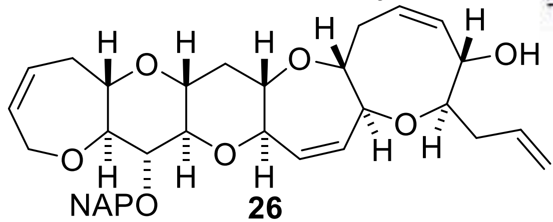

26

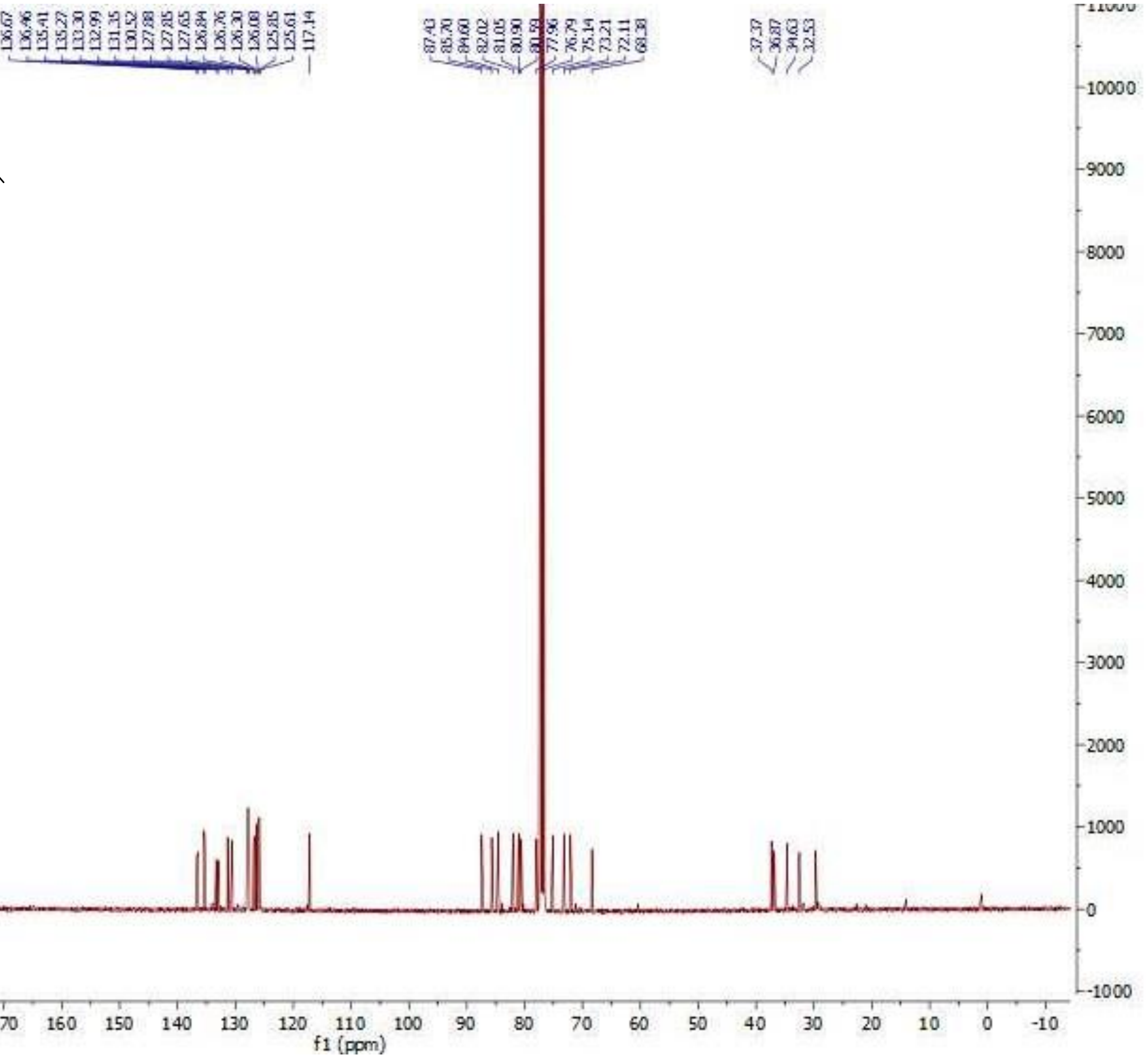

^{1}H NMR (500 MHz, $CDCl_3$)

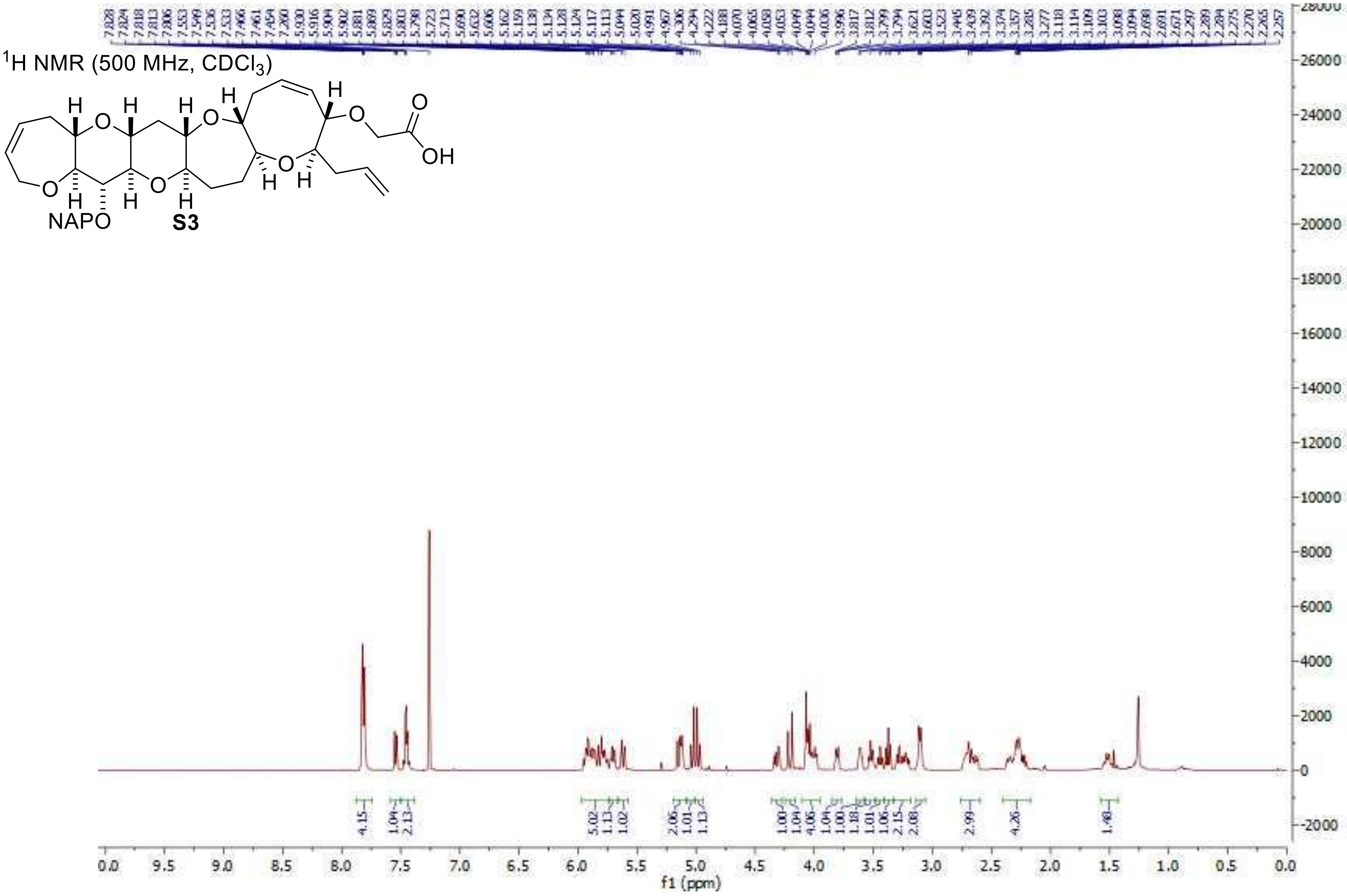

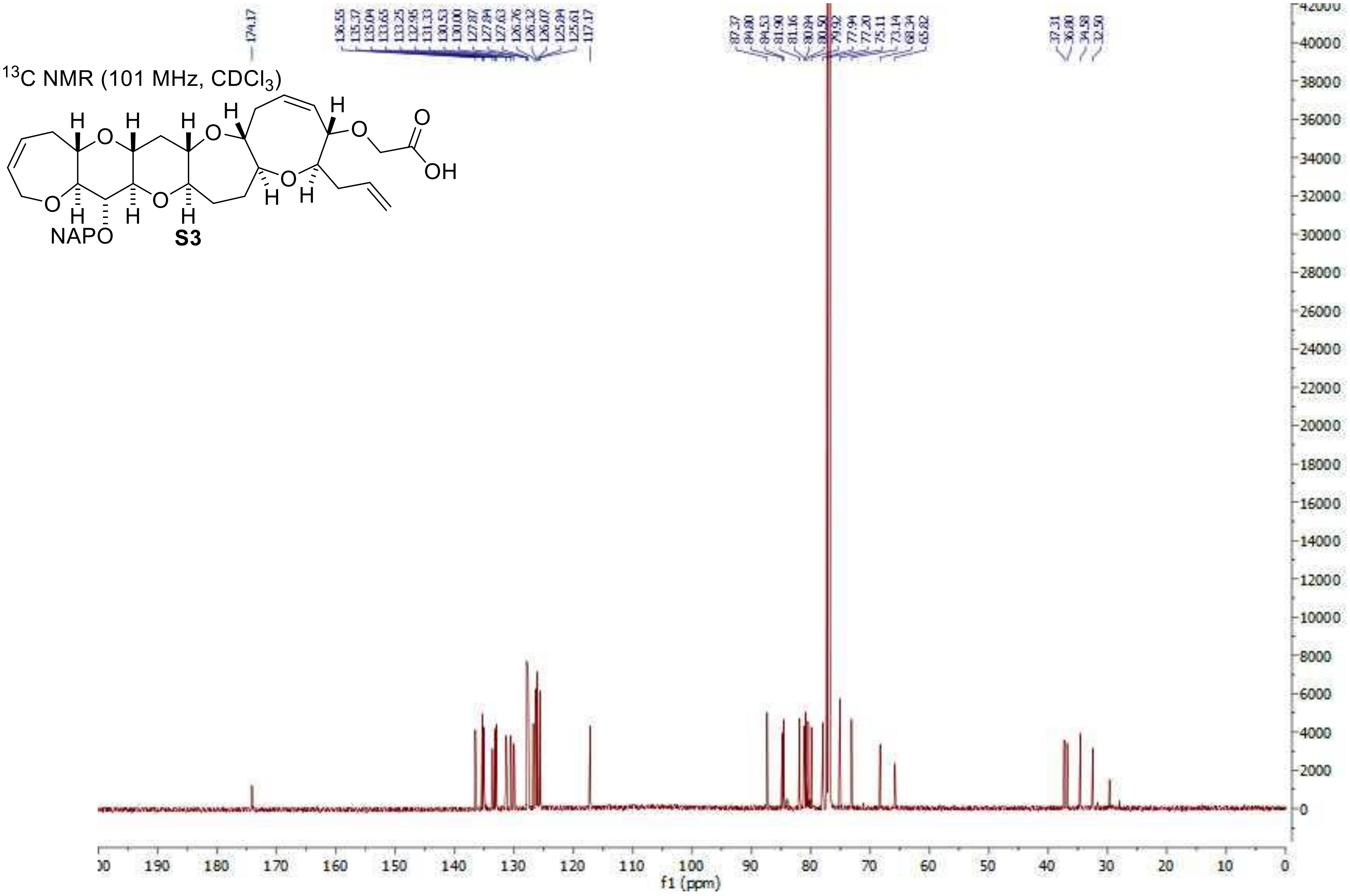

^{13}C NMR (101 MHz, CDCl$_3$)
174.17
136.55
135.37
135.04
133.65
133.25
132.95
131.33
130.53
130.00
127.87
127.84
127.63
126.76
126.32
126.07
125.94
125.61
117.17
87.37
84.80
84.53
81.90
81.16
80.84
80.50
79.92
77.94
77.20
75.11
73.14
68.34
65.82
37.31
36.80
34.58
32.50
NAPO
OH
S3
f1 (ppm)

^{1}H NMR (500 MHz, $CDCl_3$)

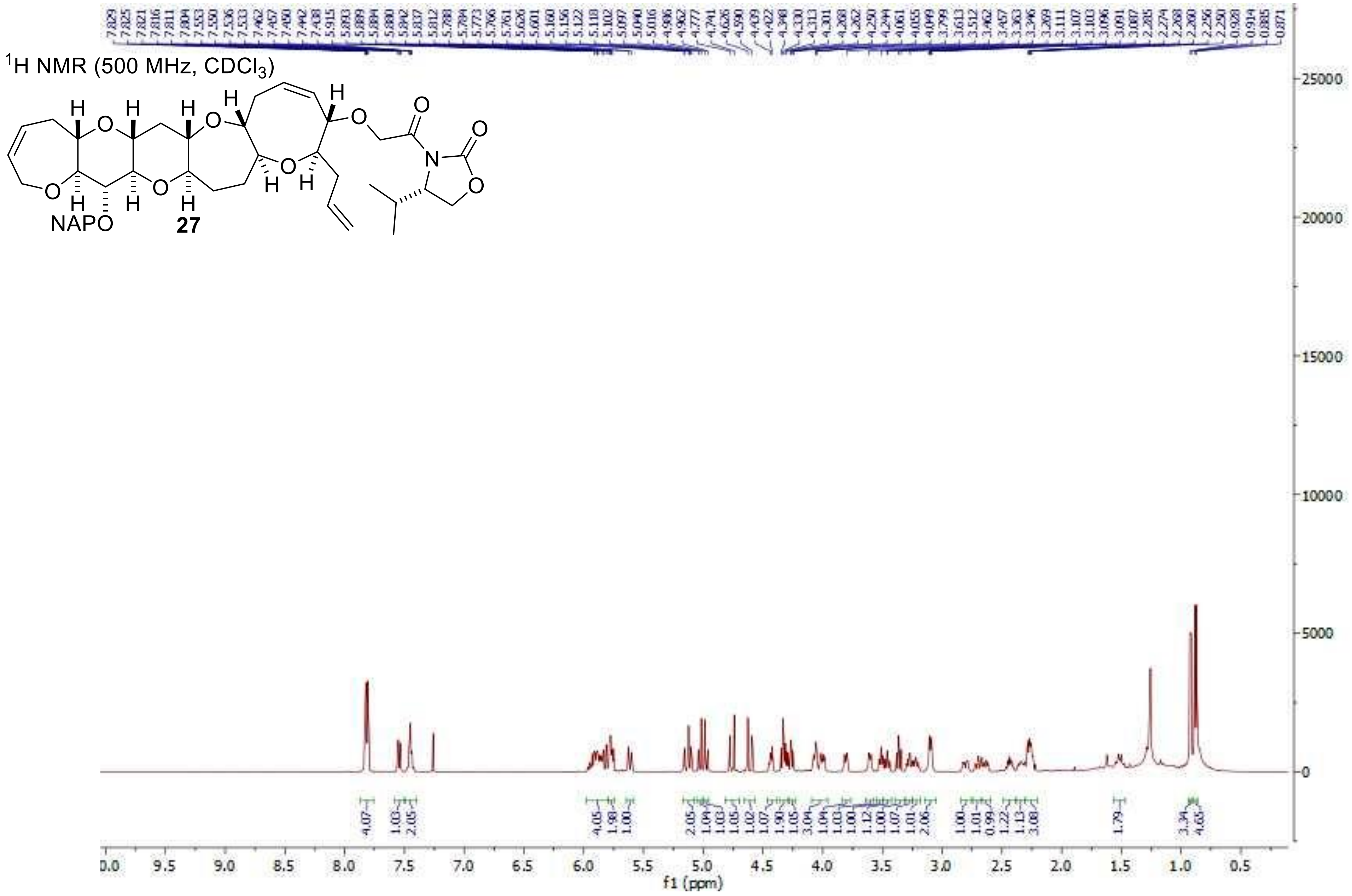

^{13}C NMR (126 MHz, $CDCl_3$)

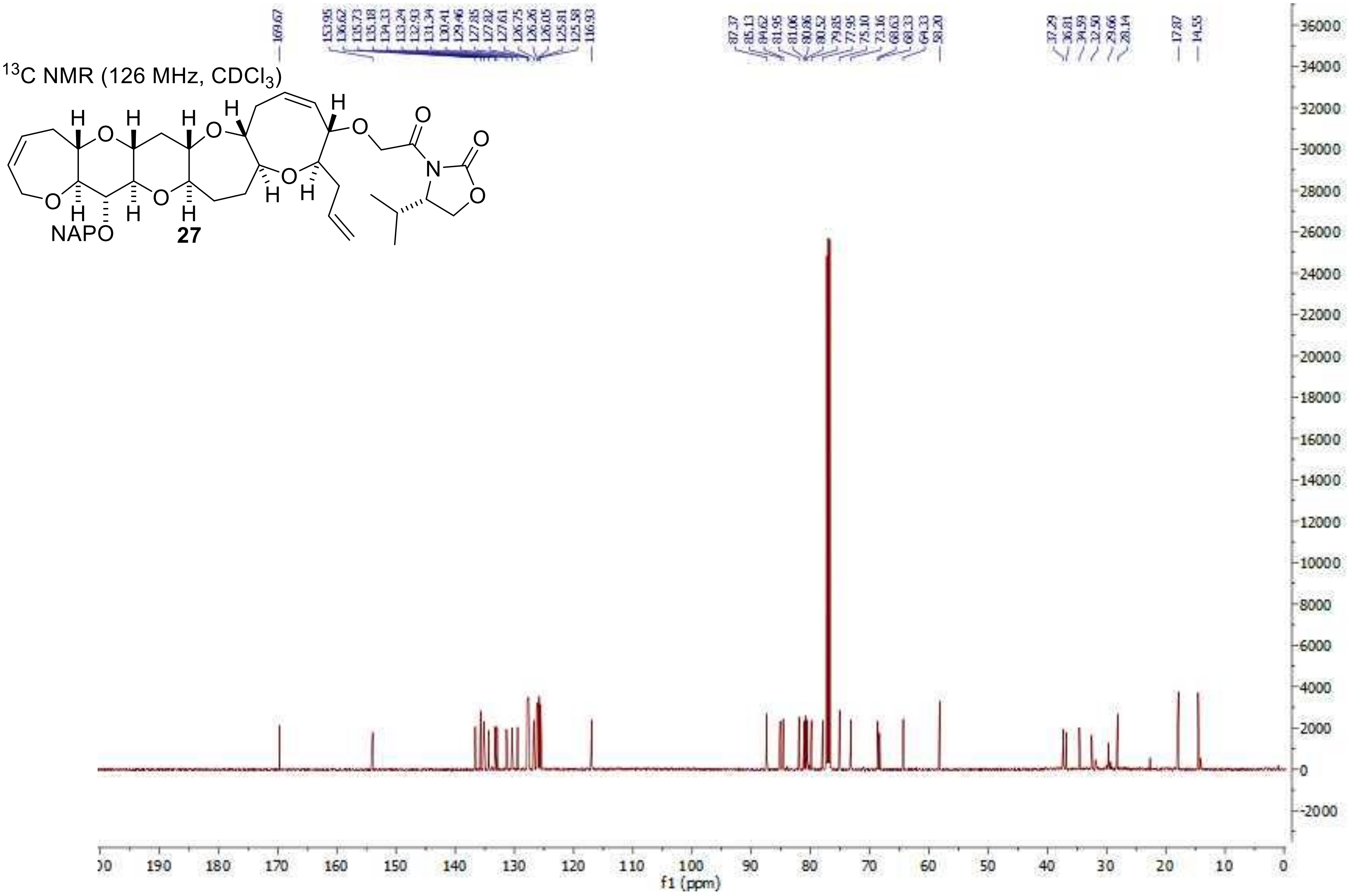

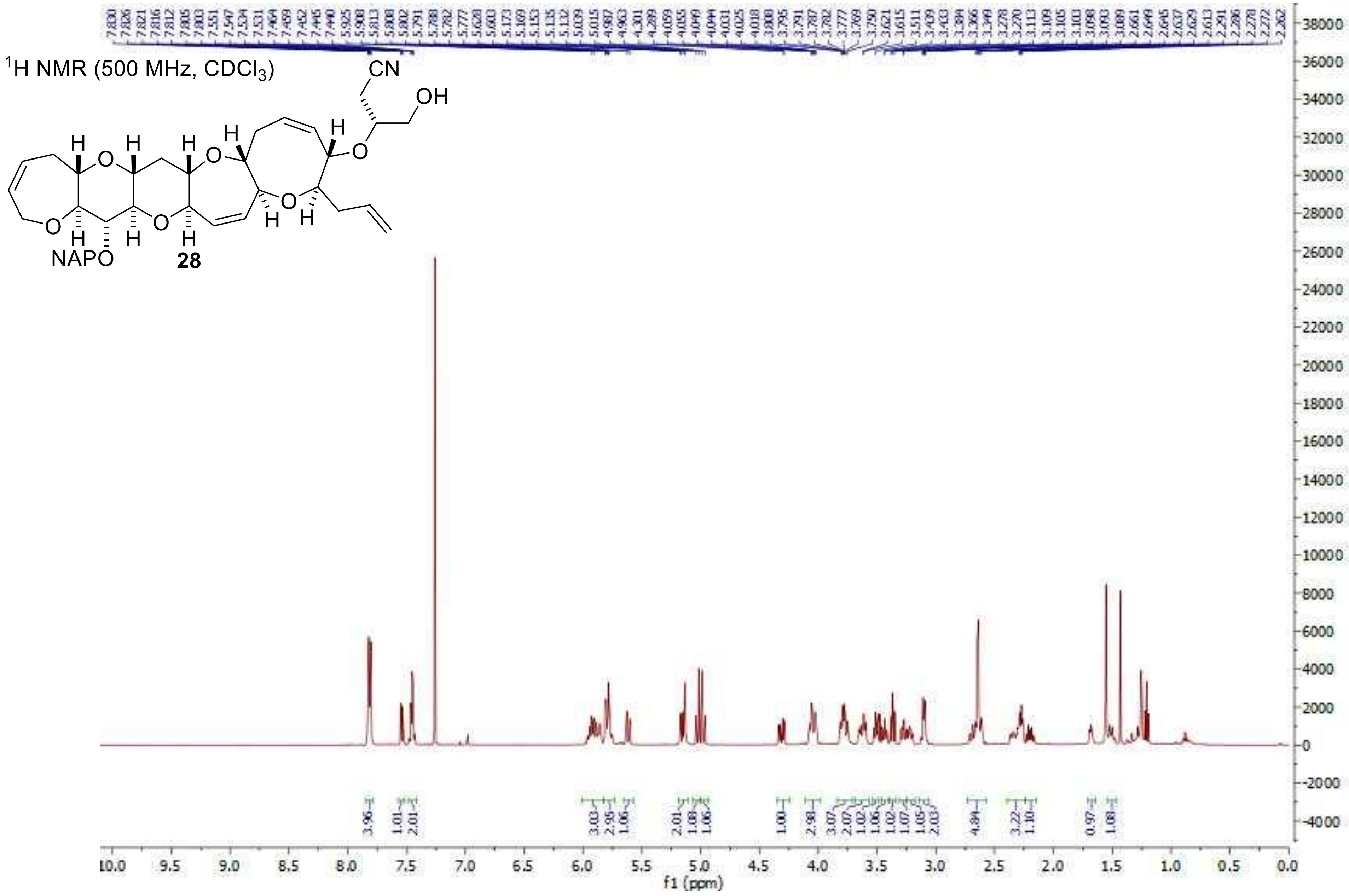

^{1}H NMR (500 MHz, CDCl$_3$)
CN
OH
NAPO
28
f1 (ppm)

^{13}C NMR (126 MHz, $CDCl_3$)

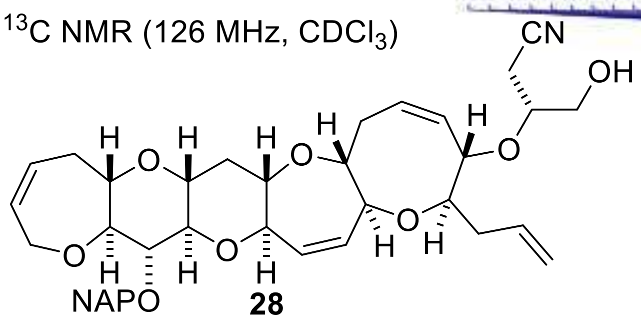

28

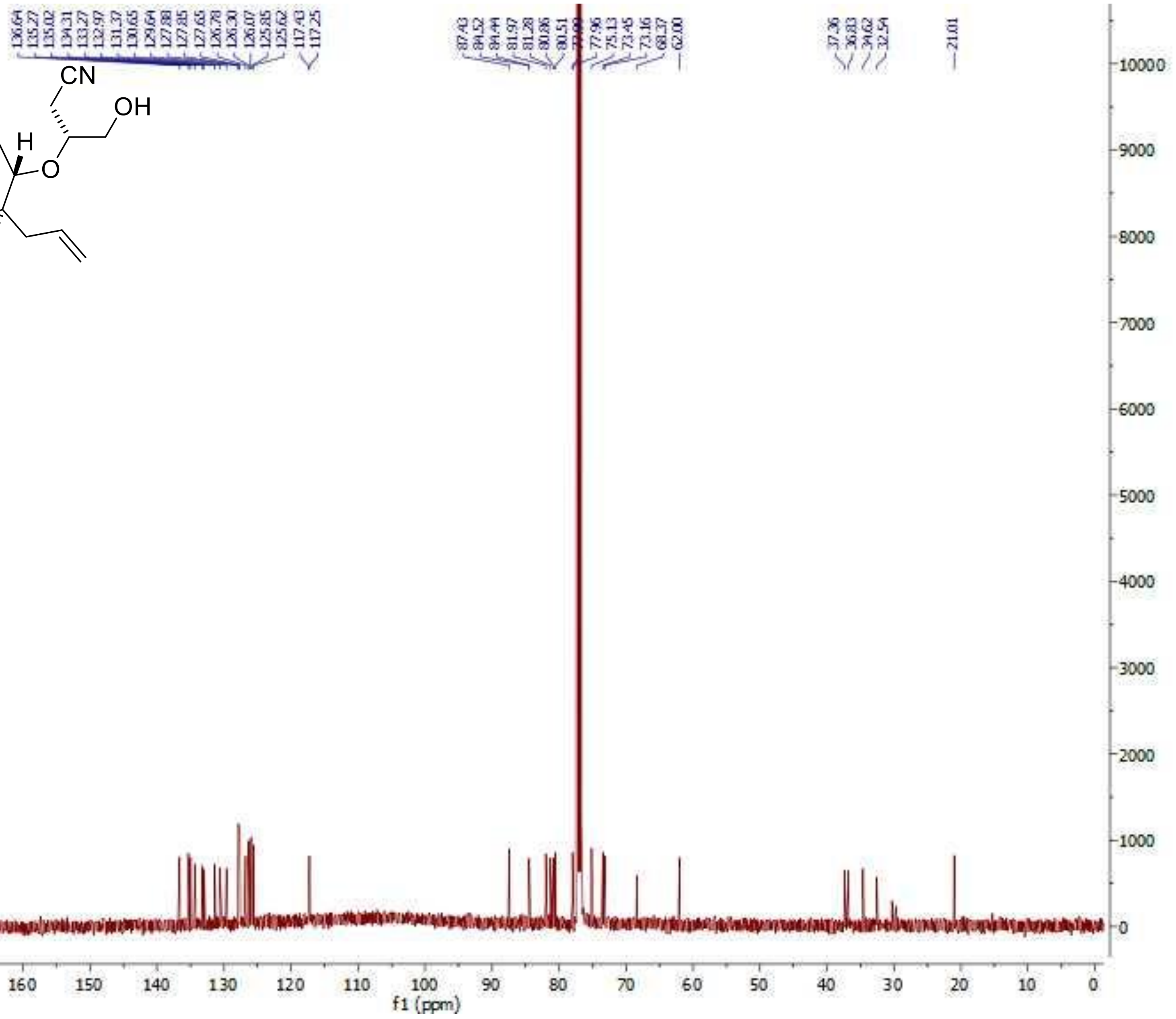

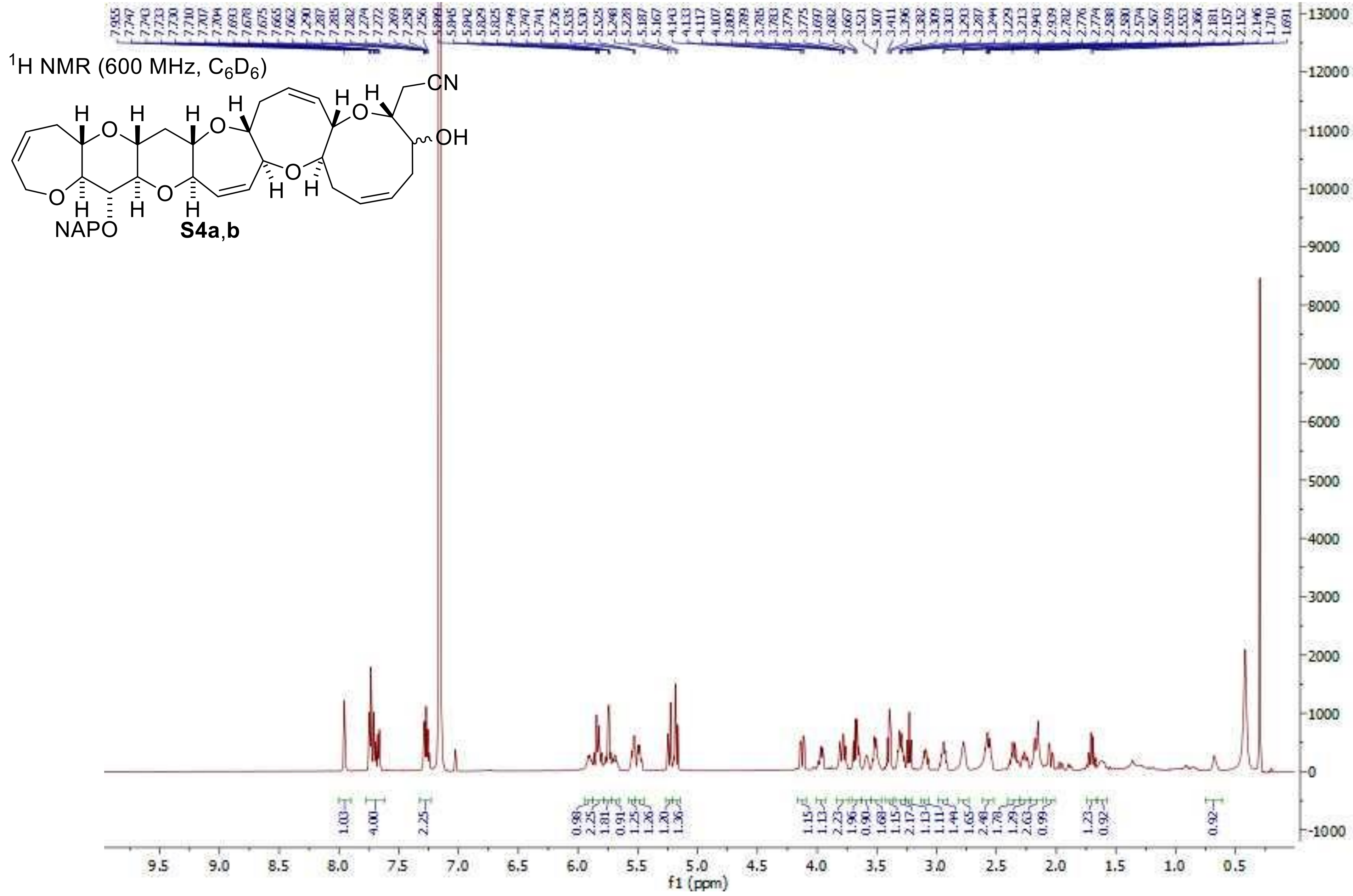

1H NMR (600 MHz, C6D6)
NAPO
S4a,b
CN
OH
f1 (ppm)

^{13}C NMR (151 MHz, C_6D_6)

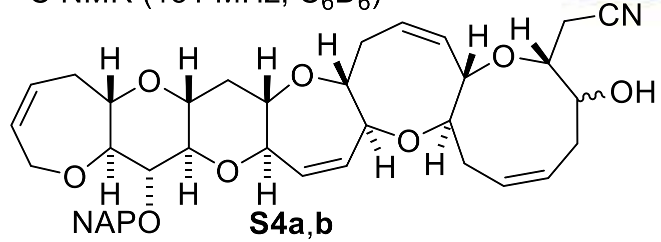

S4a,**b**

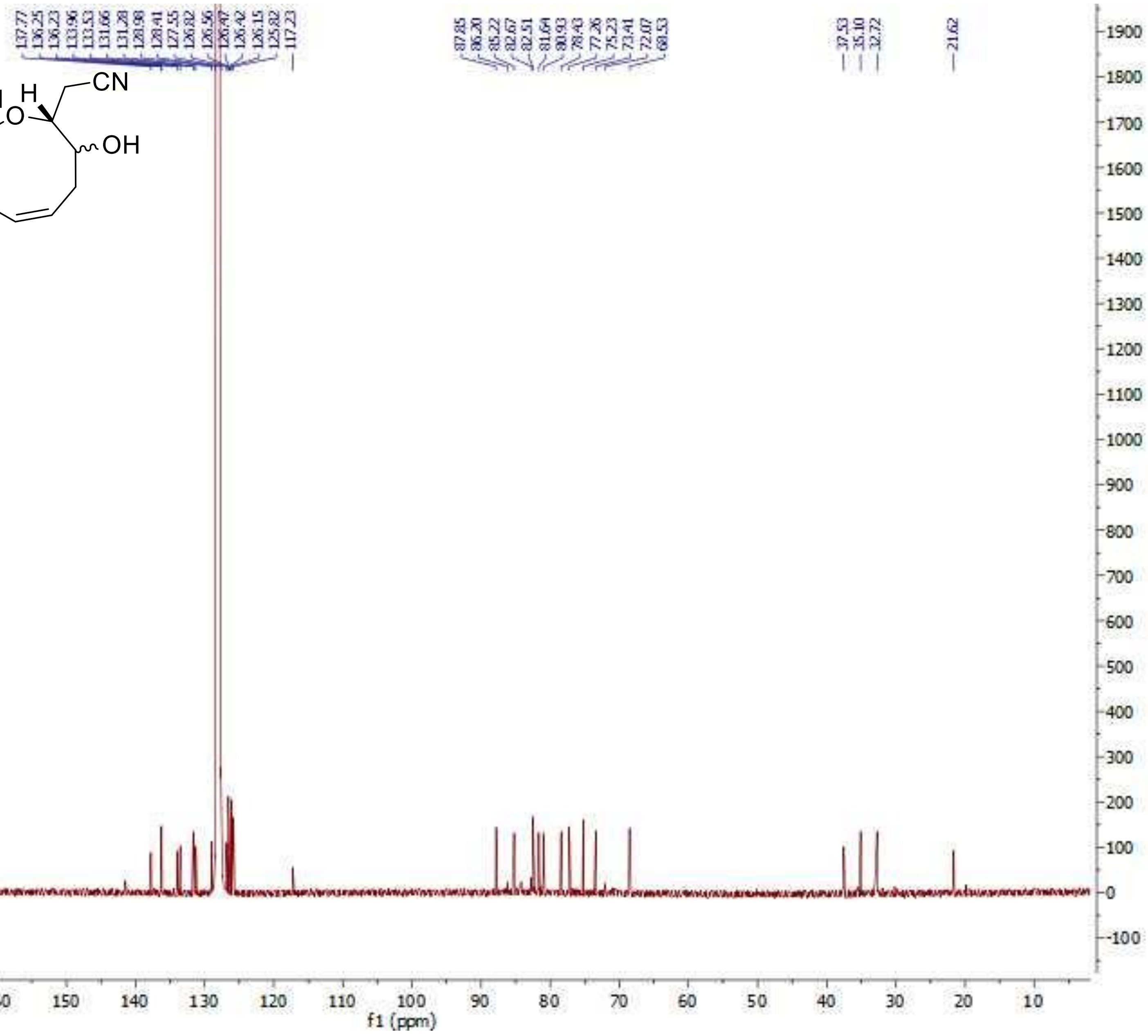

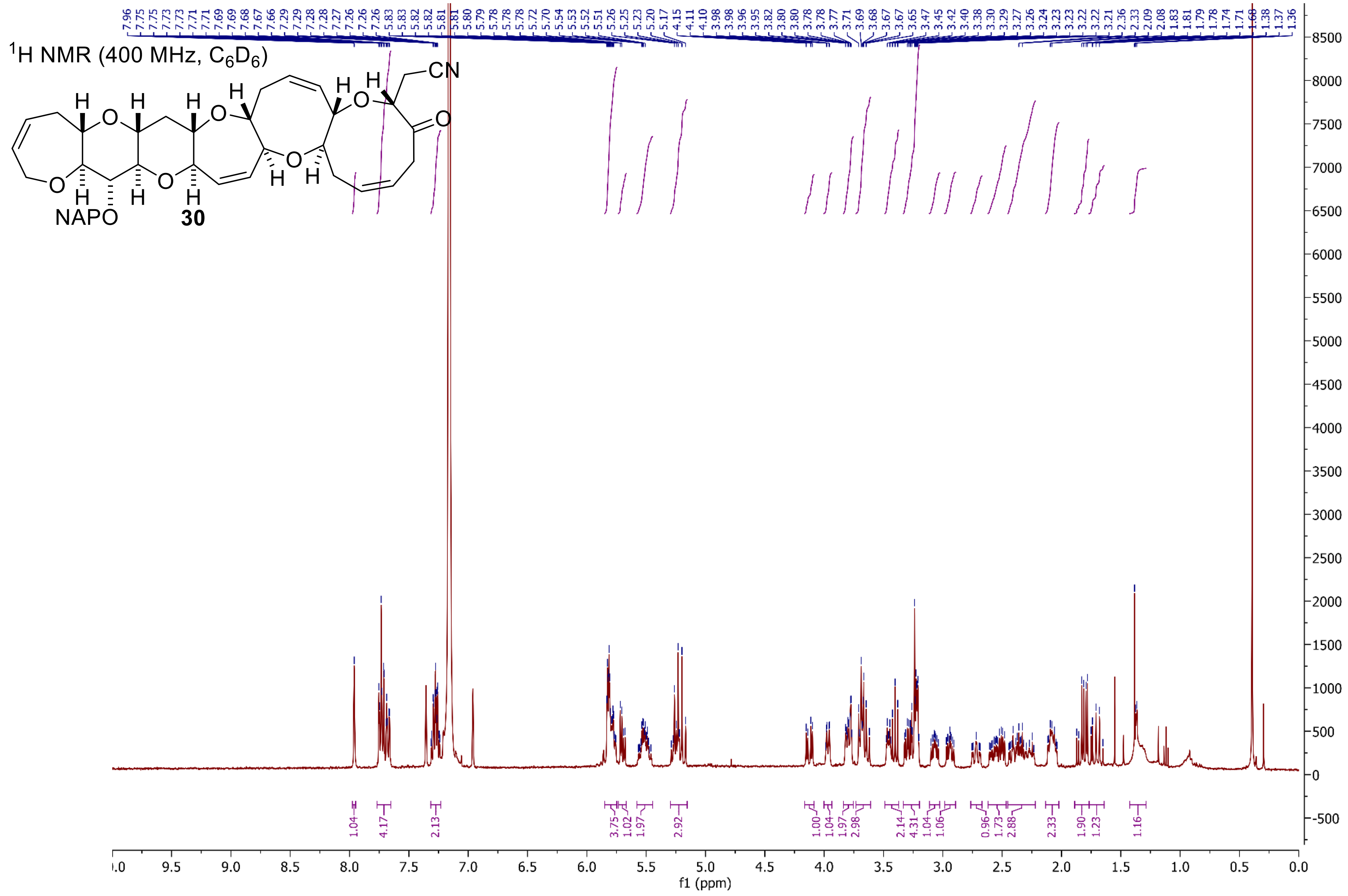

^{1}H NMR (400 MHz, C_6D_6)
30
NAPO
f1 (ppm)

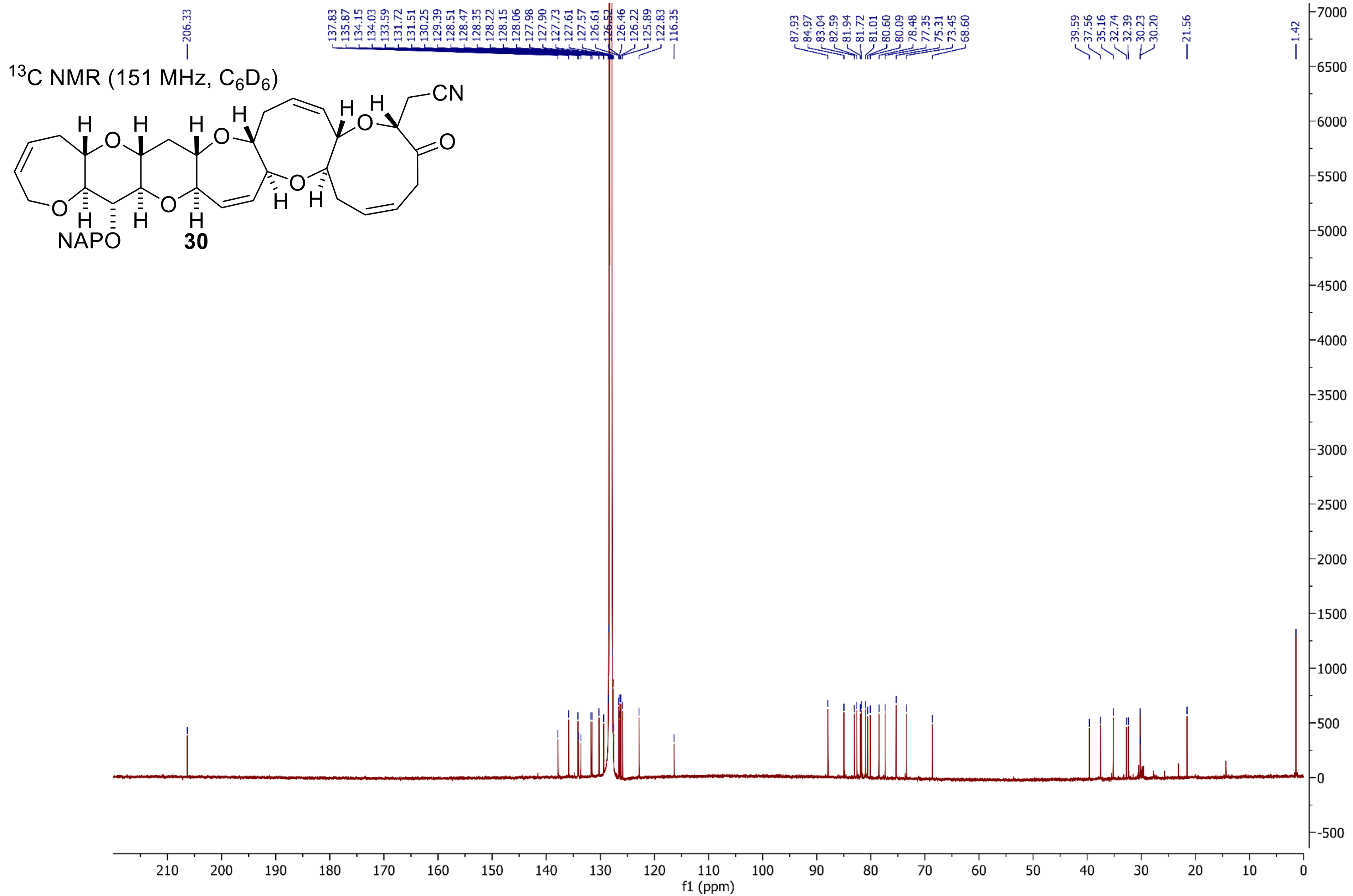

13C NMR (151 MHz, C6D6)
NAPO
30
CN
206.33
137.83
135.87
134.15
134.03
133.59
131.72
131.51
130.25
129.39
128.51
128.47
128.35
128.22
128.15
128.06
127.98
127.90
127.73
127.61
127.57
126.61
126.52
126.46
126.22
125.89
122.83
116.35
87.93
84.97
83.04
82.59
81.94
81.72
81.01
80.60
80.09
78.48
77.35
75.31
73.45
68.60
39.59
37.56
35.16
32.74
32.39
30.23
30.20
21.56
1.42
f1 (ppm)
